# Stimulation of an entorhinal-hippocampal extinction circuit facilitates fear extinction in a post-traumatic stress disorder model

**DOI:** 10.1172/JCI181095

**Published:** 2024-09-24

**Authors:** Ze-Jie Lin, Xue Gu, Wan-Kun Gong, Mo Wang, Yan-Jiao Wu, Qi Wang, Xin-Rong Wu, Xin-Yu Zhao, Michael X. Zhu, Lu-Yang Wang, Quanying Liu, Ti-Fei Yuan, Wei-Guang Li, Tian-Le Xu

**Affiliations:** 1 Department of Anesthesiology, Songjiang Research Institute, Shanghai Key Laboratory of Emotions and Affective Disorders (LEAD),; 2 Department of Anatomy and Physiology,; 3 Department of Anesthesiology, Shanghai General Hospital, and; 4 Shanghai Key Laboratory of Psychotic Disorders, Shanghai Mental Health Center, Shanghai Jiao Tong University School of Medicine, Shanghai, China.; 5 Department of Biomedical Engineering, Southern University of Science and Technology, Shenzhen, China.; 6 Department of Integrative Biology and Pharmacology, McGovern Medical School, University of Texas Health Science Center at Houston, Houston, Texas, USA.; 7 Program in Neuroscience and Mental Health, SickKids Research Institute, Toronto, Ontario, Canada.; 8 Department of Physiology, University of Toronto, Toronto, Ontario, Canada.; 9 Department of Rehabilitation Medicine, Huashan Hospital, Institute for Translational Brain Research, State Key Laboratory of Medical Neurobiology and Ministry of Education Frontiers Center for Brain Science, Fudan University, Shanghai, China.; 10 Ministry of Education–Shanghai Key Laboratory for Children’s Environmental Health, Xinhua Hospital Affiliated to Shanghai Jiao Tong University School of Medicine, Shanghai, China.; 11 Shanghai Research Center for Brain Science and Brain-Inspired Intelligence, Shanghai, China.

**Keywords:** Neuroscience, Therapeutics, Mouse models, Psychiatric diseases

## Abstract

Effective psychotherapy of post-traumatic stress disorder (PTSD) remains challenging owing to the fragile nature of fear extinction, for which the ventral hippocampal CA1 (vCA1) region is considered as a central hub. However, neither the core pathway nor the cellular mechanisms involved in implementing extinction are known. Here, we unveil a direct pathway, where layer 2a fan cells in the lateral entorhinal cortex (LEC) target parvalbumin-expressing interneurons (PV-INs) in the vCA1 region to propel low-gamma-band synchronization of the LEC-vCA1 activity during extinction learning. Bidirectional manipulations of either hippocampal PV-INs or LEC fan cells sufficed for fear extinction. Gamma entrainment of vCA1 by deep brain stimulation (DBS) or noninvasive transcranial alternating current stimulation (tACS) of LEC persistently enhanced the PV-IN activity in vCA1, thereby promoting fear extinction. These results demonstrate that the LEC-vCA1 pathway forms a top-down motif to empower low-gamma-band oscillations that facilitate fear extinction. Finally, application of low-gamma DBS and tACS to a mouse model with persistent PTSD showed potent efficacy, suggesting that the dedicated LEC-vCA1 pathway can be stimulated for therapy to remove traumatic memory trace.

## Abstract 2

Low gamma entrainment of LEC-vCA1 pathway as a mechanism for augmenting fear extinction to remove traumatic memories, providing a promising therapy for persistent PTSD.

## Graphical abstract

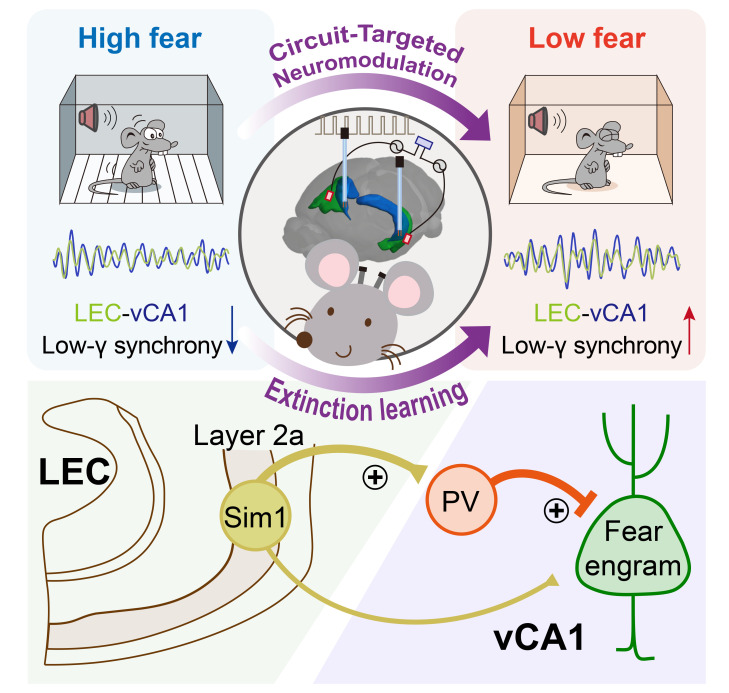

## Introduction

Fear extinction plays a pivotal role in mitigating traumatic memory, facilitating adaptive responses to dynamic environments, and is crucial in psychotherapy for anxiety disorders and post-traumatic stress disorder (PTSD) (1–3). However, current therapeutic approaches, including drugs and electromagnetic brain stimulations, often lack precision in targets and reliability in outcomes (2). This ambiguity may stem from a limited mechanistic understanding of fear extinction, hindering the development of circuit- and cell type–specific interventions. Fear extinction primarily relies on tripartite cortical-subcortical neural circuits, including medial prefrontal cortex (mPFC), basolateral amygdala (BLA), and hippocampus (4–8). Yet the core pathway and cellular mechanisms governing this tripartite circuitry that drives fear extinction remain elusive. Identification and harnessing of key top-down circuit motifs and cellular ensembles inherent in the natural extinction process hold promise for the development of neuromodulation strategies to target pathway- and cell type–specific circuits for PTSD treatment.

The hippocampus (HPC), crucial for declarative memory, receives diverse inputs from the neocortex through parahippocampal structures (9, 10), notably the entorhinal cortex (EC) (11). Structurally and functionally, the HPC is divided into the dorsal and ventral regions, associated with spatial memory and emotional processing, respectively (12). The EC comprises the lateral entorhinal cortex (LEC) and the medial entorhinal cortex (MEC), linked to object recognition and spatial learning, respectively (13–15). As a major memory hub, the entorhinal-hippocampal system coordinates projections and synchronizes neural oscillations between brain regions. Despite the well-studied entorhinal–dorsal hippocampal network supporting spatial navigation and associative memory (13–20), the connectivity, activity, and behavioral implications of the ventral hippocampal–entorhinal network remain enigmatic.

Circuit oscillations, arising from synchronized or cooperative activities among different neuronal populations, enable fast transitions between large-scale network states (21, 22). The interplay between circuit oscillations, long-term synaptic plasticity, and recruitment of memory engrams shapes the encoding and retrieval of memories (23, 24). Retrieval of fear memory correlates with amygdalar and hippocampal theta rhythm synchronization (25). Additionally, expression of fear memory involves oscillatory activity in the 3–6 Hz range within the BLA, along with coherence shifting toward the 3–6 Hz range between the BLA and mPFC (26, 27). Conversely, fear extinction remodels the network of inhibitory interneurons in the BLA, allowing a competition between a 6- to 12-Hz oscillation and the fear-associated 3- to 6-Hz oscillation (26, 28). This underscores the significance of local and inter-regional experience-dependent resonance in governing dynamic expression of fear memory. In parallel, gamma oscillations in the HPC enhance sensory processing, attention, and memory (29–32). Pathway-specific gamma oscillations facilitate task-relevant information routing between distinct neuronal subpopulations within the entorhinal-hippocampal circuit (15). These findings suggest that oscillatory activity within the entorhinal-hippocampal circuit may be related to fear extinction, representing a form of inhibitory learning. The circuit organization, along with oscillatory dynamics concerning cell type–specific connectivity between EC and ventral HPC involved in fear extinction and its potential for therapeutic neuromodulation of PTSD, remains unexplored.

Our study reveals a direct projection from LEC layer 2a fan cells to ventral hippocampal CA1 (vCA1) parvalbumin-expressing interneurons (PV-INs), distinct from established indirect trisynaptic pathways observed from LEC layer 2a to the dorsal HPC (14, 20, 33, 34). Further exploration of neural oscillations within the EC-vCA1 network reveals that extinction training is associated with heightened low-gamma rhythms and synchronization between LEC and vCA1 regions. This oscillation is mediated by vCA1 PV-INs directly innervated by LEC layer 2a fan cells. Importantly, entraining the identified LEC-vCA1 pathway with clinically available interventions, such as deep brain stimulation (DBS) and transcranial alternating current stimulation (tACS) (35–39), results in a robust attenuation of fear memory. This provides a proof of principle for alleviating traumatic memories using readily available strategies.

## Results

### Fear extinction induces low-gamma rhythm synchronization between LEC and vCA1.

**Figure 1:**
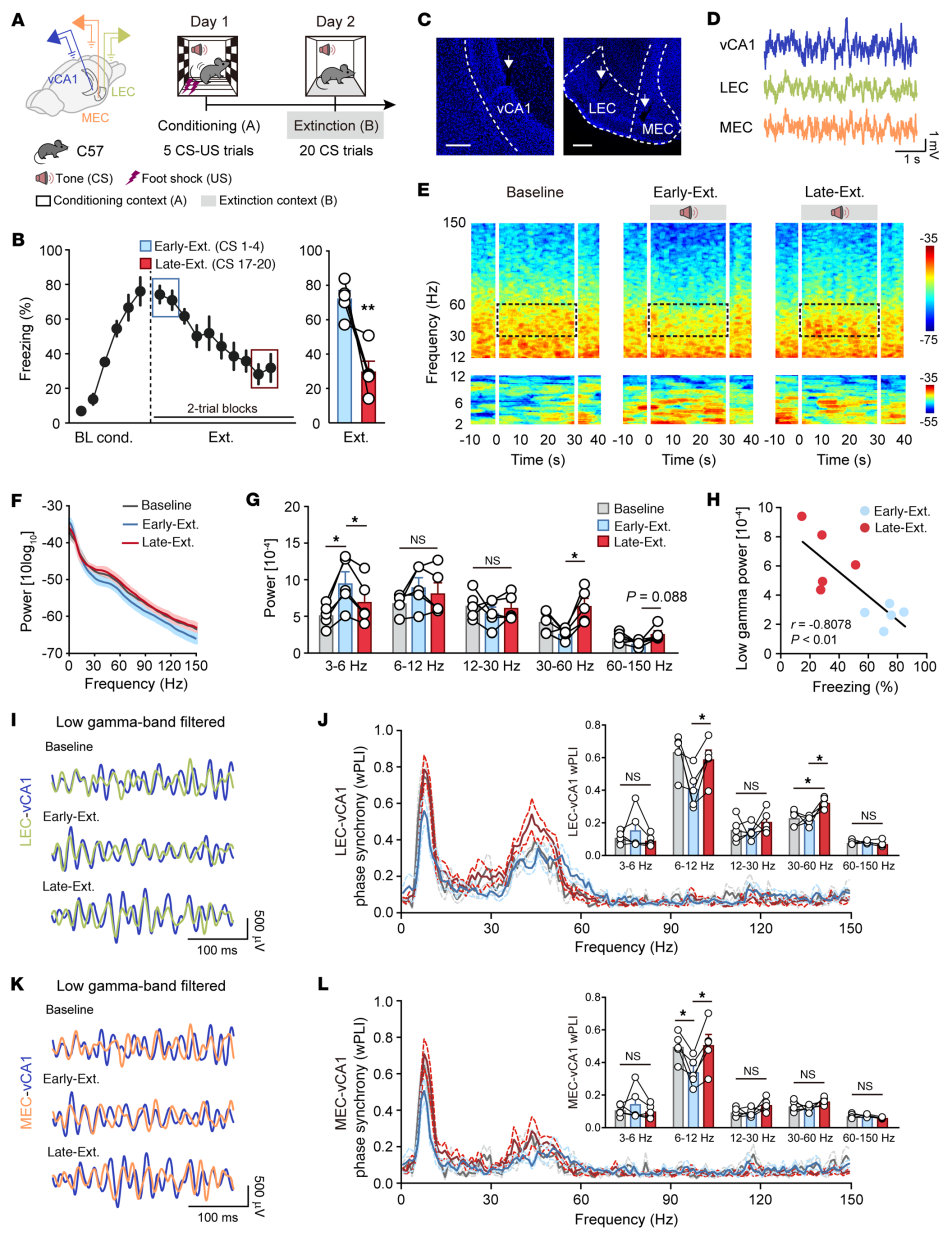
Fear extinction recruits low-gamma oscillatory synchrony between the LEC and vCA1. (**A**) Schematics of electrode implantation and experimental design for mice subject to fear conditioning (context A) and extinction training (context B). (**B**) Left: Time courses of freezing responses to the CS during fear conditioning and extinction training. Right: Freezing responses to the CS during early extinction training (CS1–4, referred to as Early-Ext.) and late extinction training (CS17–20, referred to as Late-Ext.). Data are mean ± SEM. *n* = 5 mice. **P < 0.01. (**C**) Representative images showing electrode placements. Scale bars: 200 μm. (**D**) Representative traces of LFP recordings. (**E**) Representative spectrograms of LFP recorded in vCA1 during Baseline (left), Early-Ext. (middle), and Late-Ext. (right) sessions. Zero to thirty seconds represents the tone given during extinction training. (**F**) Power spectrum of vCA1 LFP during Baseline, Early-Ext., and Late Ext. Solid lines represent averages and shaded areas indicate SEM. (**G**) Average power of vCA1 LFP during Baseline, Early-Ext., and Late Ext. Data are mean ± SEM. *n* = 5. *P < 0.05. (**H**) Linear regression of freezing responses versus vCA1 low-gamma power during Early-Ext. and Late Ext. sessions. (**I**) Examples of low-gamma-frequency filtered LEC and vCA1 LFP recordings recorded during Baseline, Early-Ext., and Late Ext. sessions. (**J**) Phase synchrony for LEC-vCA1 LFPs in the Baseline, Early-Ext., and Late-Ext. sessions, respectively. Inset shows different phase synchrony quantified using the weighted phase lag index (wPLI) between LEC and vCA1 LFPs. Data are mean ± SEM. *n* = 5. *P < 0.05. (**K** and **L**) The same as **I** and **J** for MEC-vCA1 LFPs and wPLI. *n* = 5. *P < 0.05. Paired Student’s *t* test (**B**) and repeated-measures 1-way ANOVA with Tukey’s multiple-comparison test (**G**, **J**, and **L**).

To explore the functional connectivity between the EC and vCA1 during fear extinction, we implanted electrodes in the vCA1, LEC, and MEC to record local field potentials (LFPs). Mice underwent auditory fear conditioning followed by extinction training, which resulted in a gradual reduction in freezing responses (Figure 1, A–D, and Supplemental Figure 1, A and B; supplemental material available online with this article; https://doi.org/10.1172/JCI181095DS1). LFP analysis revealed an increase in low-theta (3–6 Hz) oscillations during fear conditioning and contextual fear retrieval (conditioning context re-exposure), but not during exposure to a control auditory tone (unpaired conditioned stimulus, CS–), compared with baseline data at habituation, in the vCA1, LEC, and MEC (Supplemental Figure 1), paralleling previous findings in the BLA (26, 27). During early extinction (Early-Ext., CS1–4), both vCA1 (Figure 1, E–G) and LEC, as well as MEC (Supplemental Figure 2), exhibited increased low-theta oscillations, while high-theta (6–12 Hz) oscillations did not show a similar increase. However, during late extinction (Late-Ext., CS17–20), there was an increase in low-gamma (30–60 Hz) oscillation power in these regions. Notably, there were negative correlations between cue-induced conditioned freezing and low-gamma power across all recorded regions (Figure 1H and Supplemental Figure 2, D and G). Phase synchronization analysis using the weighted phase lag index demonstrated higher synchrony between LEC-vCA1 low-gamma oscillations during late extinction (Figure 1, I–L) and extinction retrieval (Supplemental Figure 1, I and J), underscoring their substantial role in the fear extinction process compared with MEC-vCA1 synchronization.

### Low-gamma synchronization between LEC and vCA1 during fear extinction requires the vCA1 PV-INs.

**Figure 2:**
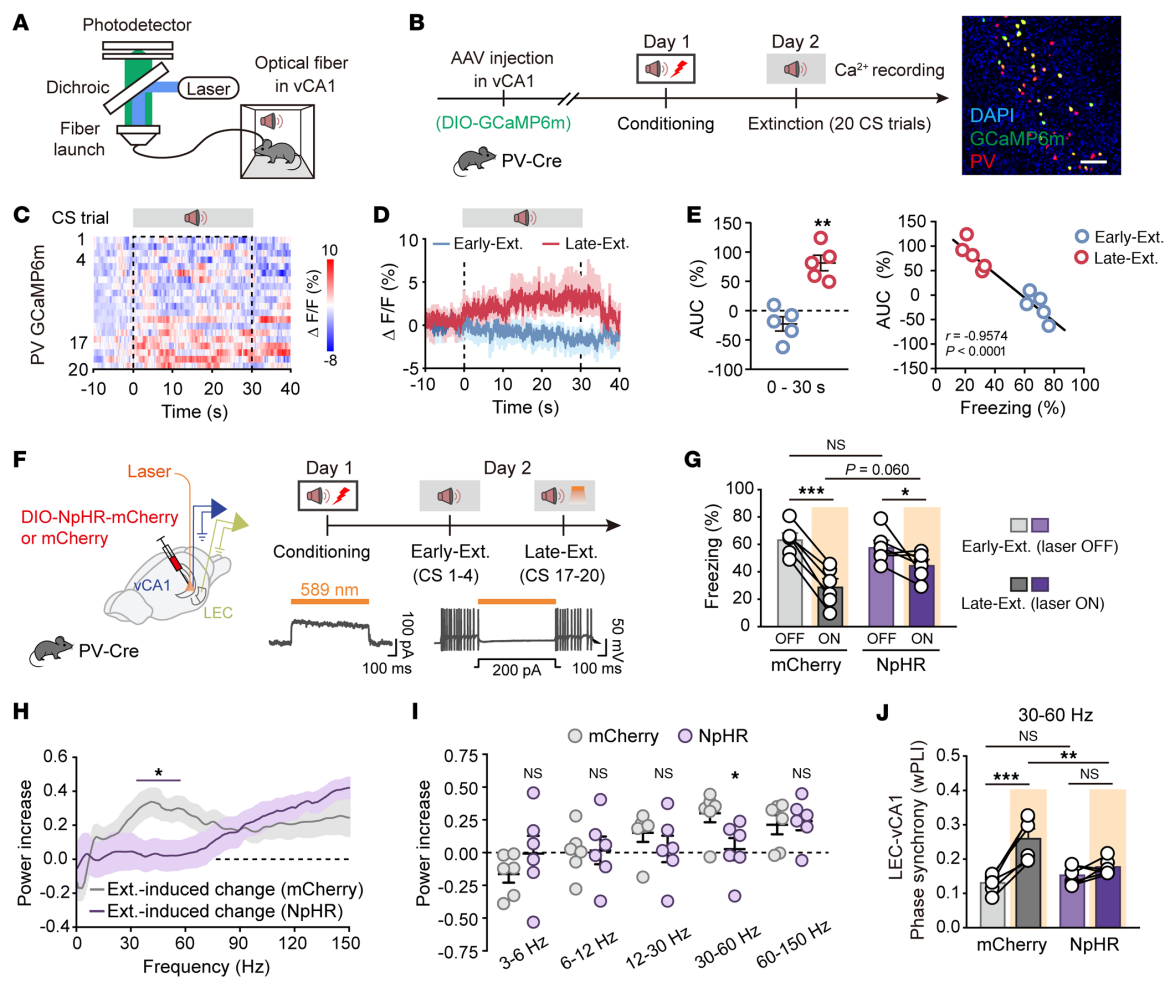
Activation of vCA1 PV-INs is required for LEC-vCA1 low-gamma synchronization during late extinction. (**A**) Schematic illustration. (**B**) Schematic of AAV injections and experimental design, as well as immunostaining confirming specificity of GCaMP6m expression in the PV-INs. Scale bar: 100 μm. (**C**) Heatmap of calcium signals in the PV-INs during extinction training. (**D**) Average PV-IN GCaMP signals. Data are mean ± SEM. *n* = 5 mice. (**E**) Activity of the PV-INs (area under the curve [AUC]) and correlation of freezing responses with the Ca^2+^ signals. Data are mean ± SEM. **P < 0.01. (**F**) Schematics of stereotaxic surgery and experimental design. (**G**) Freezing responses to the CS during Early-Ext. and Late-Ext. *n* = 6 mice per group. Data are mean ± SEM. *P < 0.05, ***P < 0.001, light × group interaction, *F*_1,10_ = 9.356, *P* = 0.0121. (**H**) Extinction-induced changes in power spectrum of vCA1 LFP. Shown is mean ± SEM of power (Late-Ext. – Early-Ext.)/(Late-Ext. + Early-Ext.). *n* = 6 mice per group. Purple line indicates frequencies with a significant effect (*P < 0.05 with Bonferroni’s correction for multiple comparisons). (**I**) Average power increase of vCA1 LFP. Data are mean ± SEM. *n* = 6 mice per group. Main effect of AAV, *F*_1,10_ = 0.122, *P* = 0.7341. *P < 0.05. (**J**) Low-gamma phase synchrony quantified using the wPLI between LEC and vCA1 LFPs. Data are mean ± SEM. *n* = 6 mice per group. **P < 0.01, ***P < 0.001, light × group interaction, *F*_1,10_ = 15.80, *P* = 0.0026. Paired Student’s *t* test (**E**), repeated-measures 2-way ANOVA with Šidák’s multiple-comparison test (**G** and **J**), Wilcoxon’s signed-rank test with Bonferroni correction for multiple comparisons (**H**), and repeated-measures 2-way ANOVA and unpaired Student’s *t* test (**I**).

Neuronal oscillations result from the dynamic interplay between excitation and inhibition (21, 22, 40), with inhibitory interneurons (41), including PV-INs, somatostatin-expressing interneurons (SST-INs), and vasoactive intestinal peptide–expressing interneurons (VIP-INs), orchestrating synchronized activity in the HPC. To identify the specific interneuron subtype responsible for network reorganization during fear extinction, we selectively labeled GABAergic neurons in vCA1 by using AAV-DIO-mCherry in PV-Cre, SST-Cre, and VIP-Cre mice (Supplemental Figure 3A). Fear extinction selectively activated PV-INs, as indicated by increased PV-mCherry^+^c-Fos^+^ cells compared with SST-INs or VIP-INs (Supplemental Figure 3, B and C). This was further corroborated by in vivo Ca^2+^ recordings using fiber photometry, which detected cell type–specific GCaMP6m fluorescence changes and confirmed the specific activation of PV-INs in vCA1 during fear extinction (Figure 2, A and B, and Supplemental Figure 3D). Real-time Ca^2+^ signals showed increased PV-IN activity during the Late-Ext. phase (Figure 2, C–E), while SST-INs and VIP-INs did not exhibit significant changes (Supplemental Figure 3, E–L), reinforcing the unique role of PV-INs in the process. Notably, PV-INs displayed much higher Ca^2+^ signals in response to footshock as the unconditioned stimulus (US), but not to the auditory tone as the CS during fear conditioning. There was no significant Ca^2+^ signal during contextual fear retrieval or exposure to a control auditory tone (CS–), but there was a prominent signal during extinction retrieval compared with baseline (Supplemental Figure 4).

To assess the role of PV-INs in neural oscillations during fear extinction, we bilaterally injected AAV-DIO-NpHR-mCherry or control virus into the vCA1 of PV-Cre mice, implanted optical fibers targeting vCA1, and placed LFP electrodes in both vCA1 and LEC (Figure 2F and Supplemental Figure 5). Optical inhibition of vCA1 PV-INs during the Late-Ext. phase resulted in a tendency to increase the cue-induced freezing in comparison with the control group (Figure 2G). In the control group, there was an observed increase in low-gamma oscillations in vCA1 during the Late-Ext. phase (Figure 2H). However, this increase, along with LEC-vCA1 synchronization, was disrupted by the inhibition of PV-INs (Figure 2, I and J). These findings highlight the critical role of vCA1 PV-INs in facilitating fear extinction, possibly through promoting LEC-vCA1 low-gamma synchronization.

### LEC Sim1^+^ layer 2a fan cells are the main projection neurons to vCA1 PV-INs.

**Figure 3:**
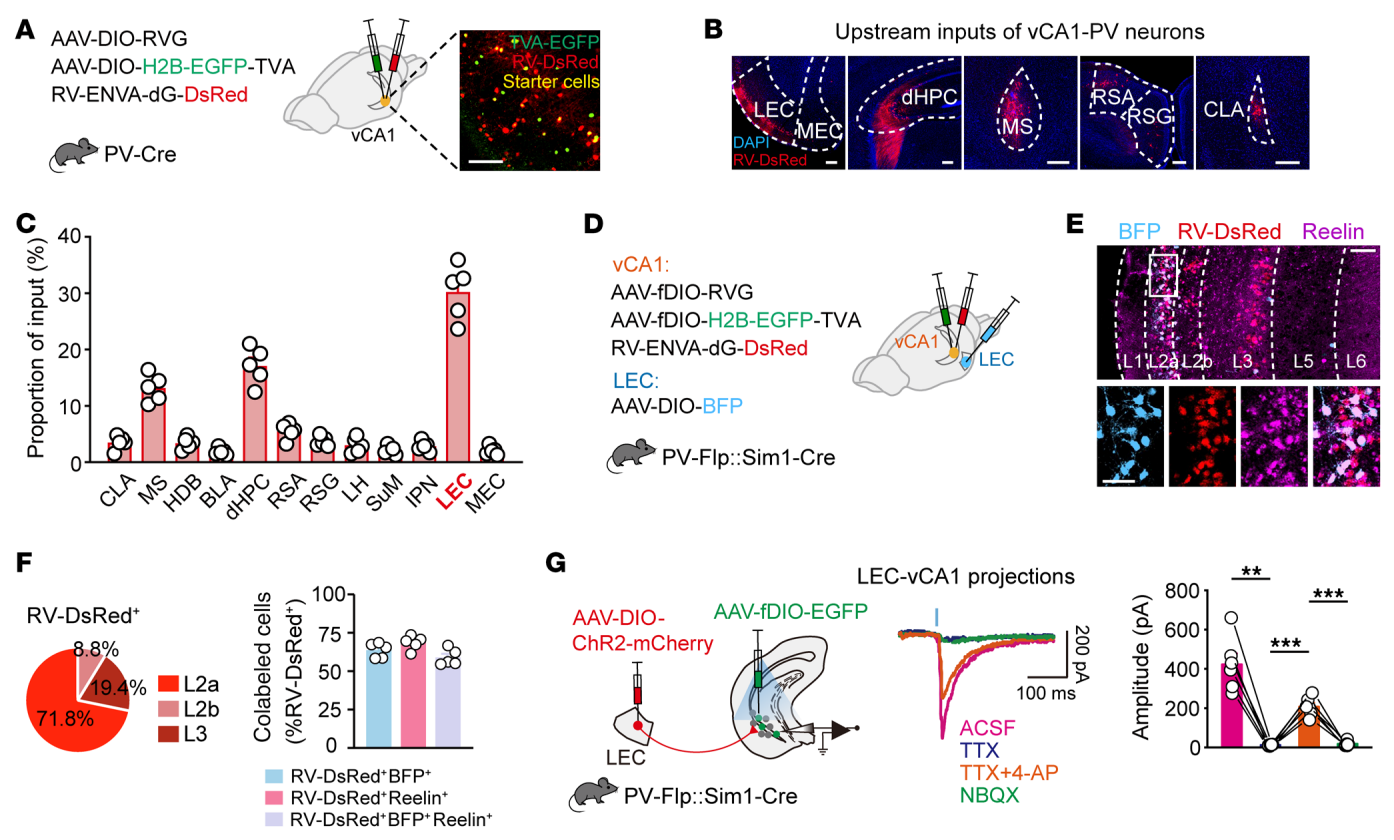
vCA1 PV-INs receive strong excitatory inputs from Sim1^+^ fan cells in LEC layer 2a. (**A**) Schematic of AAV injections and experimental design (left) and a representative image of TVA-EGFP and RV-DsRed expression (right). Scale bar: 100 μm. (**B**) Representative images of the main upstream inputs. Scale bars: 200 μm. (**C**) Distribution of RV-DsRed–labeled neurons. *n* = 5 mice. CLA, claustrum; MS, medial septal nucleus; HDB, nucleus of the horizontal limb of the diagonal band; BLA, basolateral amygdalar nucleus; dHPC, dorsal hippocampus; RSA, retrosplenial agranular cortex; RSG, retrosplenial granular cortex; LH, lateral hypothalamic; SuM, supramammillary nucleus; IPN, interpeduncular nucleus; LEC, lateral entorhinal cortex; MEC, medial entorhinal cortex. (**D**–**F**) LEC layer 2a–vCA1 PV-IN projectors are Sim1^+^ fan cells. (**D**) Schematic of AAV injections. (**E**) Representative images of BFP^+^ (blue), RV-DsRed^+^ (red), and Reelin^+^ (purple) immunofluorescence in LEC. Scale bars: 100 μm (top), 50 μm (bottom). (**F**) LEC neurons projecting to vCA1 PV-INs are mainly located in layer 2a (left) and are characterized by the expression of Reelin (right). *n* = 5. (**G**) Patch clamp recordings of activity of vCA1 PV-INs in brain slices upon optogenetic stimulation of LEC layer 2a–vCA1 projection (left), showing example traces evoked by blue lights in the presence of ACSF, TTX (1 μM), TTX plus 4-AP (100 μM), and NBQX (10 μM). The blue vertical bar above traces indicates photostimulation. *n* = 6 neurons. **P < 0.01, ***P < 0.001, repeated-measures 1-way ANOVA with Tukey’s multiple-comparison test.

To map the monosynaptic inputs to vCA1 PV-INs, we used Cre-dependent rabies virus–mediated (RV-mediated) retrograde tracing in PV-Cre mice. We identified starter PV-INs (EGFP^+^ and DsRed^+^) in vCA1 (Supplemental Figure 6), and DsRed^+^ neurons outside vCA1 served as long-range presynaptic neurons (Figure 3A). These PV-INs received inputs primarily from LEC, dorsal hippocampus (dHPC), and medial septal nucleus (MS), with fewer inputs from MEC (Figure 3, B and C, and Supplemental Figure 7).

The LEC neurons projecting to vCA1 PV-INs were primarily located in the superficial sublayer 2a (Figure 3B and Supplemental Figure 7B), which is rich in Reelin-positive fan cells (14, 20, 33, 34). To identify these fan cells, we selectively labeled Sim^+^ layer 2a fan cells receiving retrograde signals from vCA1 PV-INs using an intersectional strategy in PV-Flp Sim1-Cre mice (Figure 3D). Flp-dependent trans-synaptically labeled presynaptic neurons (DsRed^+^) were mainly in layer 2a (Figure 3E), with the majority coexpressing DsRed and Cre-dependent blue fluorescent protein (BFP), confirming that LEC Sim1^+^ layer 2a fan cells, rather than layer 2b or layer 3 cells, are the principal projection neurons to vCA1 PV-INs (Figure 3F). Furthermore, using AAV with Cre-dependent expression of ChR2 in LEC Sim1^+^ layer 2a fan cells, we recorded light-induced excitatory postsynaptic currents in vCA1 PV-INs, confirming monosynaptic glutamatergic connections between LEC and vCA1 PV-INs (Figure 3G). These findings suggest that LEC Sim1^+^ layer 2a fan cells primarily mediate direct excitatory input to vCA1 PV-INs, thereby contributing to the neural circuitry responsible for fear extinction.

### Pathway from LEC layer 2a fan cells to vCA1 PV-INs orchestrates their synchronization and fear extinction.

**Figure 4:**
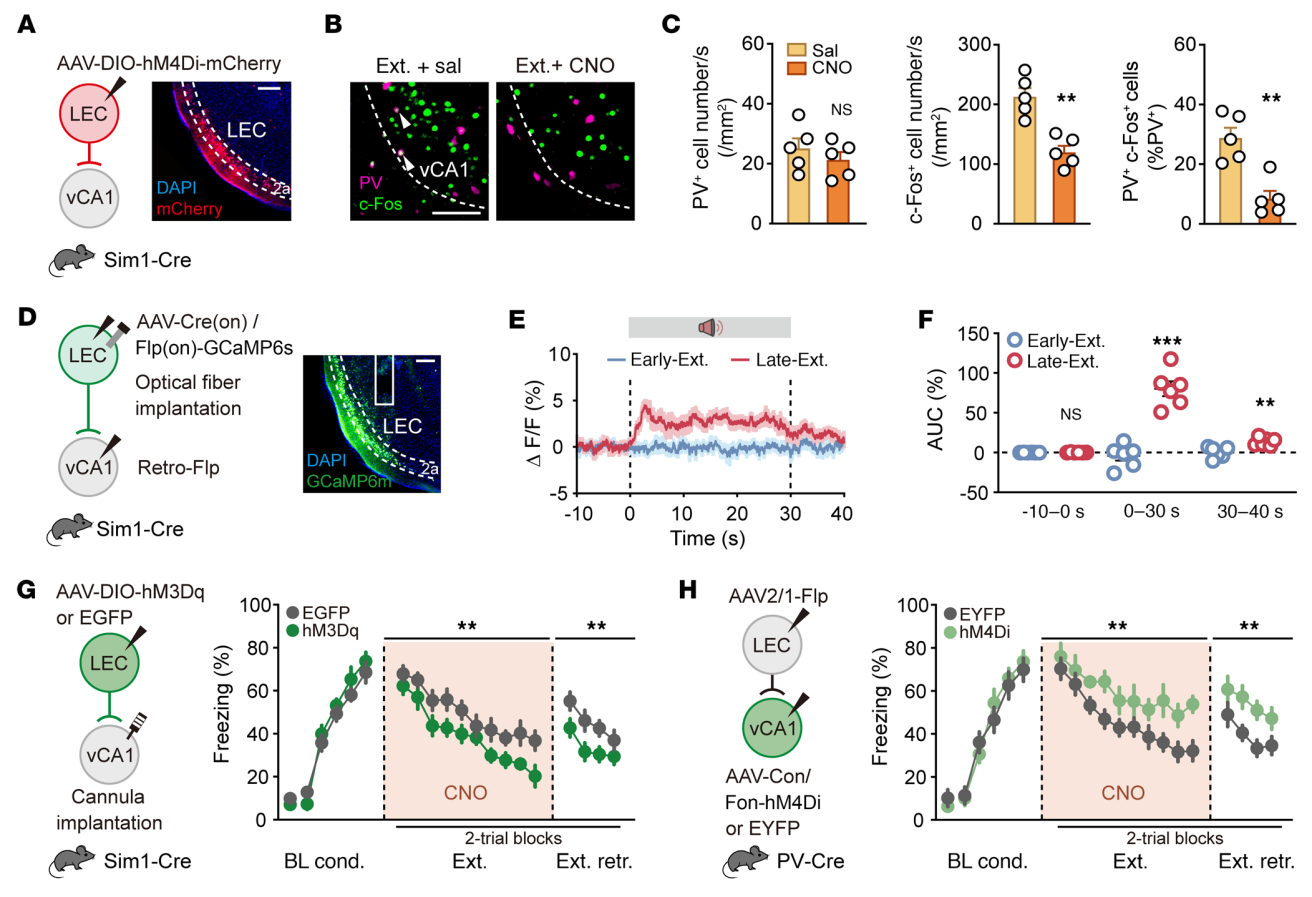
Direct projection from LEC Sim1^+^ layer 2a fan cells to vCA1 PV-INs mediates fear extinction. (**A**) Schematic of AAV injections and experimental design (left) and representative image of mCherry expression (right). CNO was administered (i.p.) 30 minutes before extinction training. Scale bar: 200 μm. (**B**) Representative images of PV^+^ (purple) and c-Fos^+^ (green) immunofluorescence. White arrowheads denote colabeled cells. Scale bar: 100 μm. (**C**) Quantification for **B**. *n* = 5 mice per group. (**D**–**F**) Ca^2+^ recording of the LEC-vCA1 pathway during extinction. (**D**) Schematic of AAV injections and fiber implantation (left), with representative images of GCaMP6s expression (right). Scale bar: 200 μm. (**E**) Average calcium signals during Early-Ext. and Late-Ext. (**F**) Activity of Ca^2+^ signals (AUC) during Early-Ext. and Late-Ext. Data are mean ± SEM. *n* = 6 mice. (**G** and **H**) Effects of stimulating LEC layer 2a→vCA1 projection (**G**) and inhibiting LEC→vCA1 PV-IN projection (**H**) on extinction. Left: Schematic of AAV injections. Right: Time courses of freezing responses to the CS. Statistics are as follows: Main effect of AAV: (**G**) Conditioning, *F*_1,17_ = 1.157, *P* = 0.2971; extinction training, *F*_1,17_ = 8.686, *P* = 0.0090; extinction retrieval, *F*_1,17_ = 9.781, *P* = 0.0061. EGFP group, *n* = 10 mice; hM3Dq group, *n* = 9 mice. (**H**) Conditioning, *F*_1,14_ = 0.1024, *P* = 0.7537; extinction training, *F*_1,14_ = 14.23, *P* = 0.0021; extinction retrieval, *F*_1,14_ = 12.46, *P* = 0.0033. EYFP group, *n* = 8 mice; hM4Di group, *n* = 8 mice. Data are mean ± SEM. **P < 0.01, ***P < 0.001. Unpaired Student’s *t* test (**C**), paired Student’s *t* test (**F**), and repeated-measures 2-way ANOVA (**G** and **H**).

To confirm the functional role of LEC layer 2a neurons in activating vCA1 PV-INs during fear extinction, we used chemogenetic inhibition with designer receptors activated only by designer drugs (DREADD) in Sim1-Cre mice (Figure 4A). Bilateral injections of AAV-DIO-hM4Di-mCherry into the LEC, followed by administration of clozapine-*N*-oxide (CNO), significantly reduced the activation of vCA1 PV-INs induced by fear extinction compared with the saline control (Figure 4, B and C, and Supplemental Figure 8A). Considering the potential indirect pathway from LEC layer 2a fan cells to vCA1 via ventral dentate gyrus (vDG) and ventral hippocampal CA3 (vCA3) (Supplemental Figure 9A), we used inhibitory optogenetic inhibition in Sim1-Cre mice. We implanted optical fibers targeting vCA1, vCA3, and vDG and delivered light during fear extinction following bilateral injections of AAV-DIO-NpHR-mCherry into the LEC. This significantly reduced activation in vCA1 PV-INs, but not in vCA3 or vDG (Supplemental Figure 9, B–K), highlighting the importance of the direct LEC-vCA1 projection in fear extinction.

Fiber photometry revealed significant increases in Ca^2+^ signals in vCA1-projecting LEC Sim1^+^ layer 2a fan cells during cue-induced activity in the Late-Ext. phase (Figure 4, D–F, and Supplemental Figure 8B) and extinction retrieval and in response to footshock as the US during fear conditioning (Supplemental Figure 10). In contrast, minimal changes were observed in dHPC-vCA1 or MS-vCA1 pathways (Supplemental Figure 11). Notably, significant Ca^2+^ signal increases were detected in the vCA1 terminals, but not in the vCA3 or vDG terminals, from LEC Sim1^+^ layer 2a fan cells during these phases (Supplemental Figure 12). Consistently, optogenetic inhibition of the projections from LEC Sim1^+^ layer 2a fan cells to vCA1, but not to vCA3 or vDG, significantly attenuated fear extinction (Supplemental Figure 13), further supporting the critical role of the direct projection from LEC Sim1^+^ layer 2a fan cells to vCA1 PV-INs in the fear extinction process.

To further explore the role of LEC Sim1^+^ layer 2a fan cells in neural oscillations during fear extinction, we bilaterally injected AAV-DIO-NpHR-mCherry into the LEC of Sim1-Cre mice (Supplemental Figure 14, A and B). Silencing these fan cells with light activation of NpHR abolished the Late-Ext.–associated increases in low-gamma power and synchronization (Supplemental Figure 14, C–F). Additionally, by using chemogenetic activation (DREADD hM3Dq) in Sim1-Cre mice (AAV-DIO-hM3Dq-EGFP, with AAV-DIO-EGFP as a control), we enhanced the presynaptic activity of fan cells. Local perfusion of CNO (1 mM, 200 nL) into the axon projection fields in vCA1 significantly reduced freezing levels during both extinction training and retrieval compared with controls (Figure 4G and Supplemental Figure 15A). Conversely, targeting an inhibitory DREADD hM4Di (or a control virus without the hM4Di effector) in a Cre- and Flp-dependent (Cre_on_/Flp_on_) manner into vCA1 PV-INs that receive projections from LEC (with an anterogradely trans-synaptic AAV2/1-Flp virus injected into LEC), we chemogenetically inhibited this subpopulation of PV-INs with CNO, leading to significant increases in freezing during extinction training and retrieval (Figure 4H and Supplemental Figure 15B). These bidirectional manipulations did not affect fear conditioning, contextual fear retrieval, or behavioral performance in the open field nor cause conditioned place preference or aversion (Supplemental Figure 16). These results underscore the necessity and sufficiency of the functional connectivity between LEC Sim1^+^ layer 2a fan cells and vCA1 PV-INs in fear extinction, establishing the LEC-vCA1 pathway as a crucial top-down motif.

### vCA1 DBS selectively recruits PV-INs to entrain vCA1 into low-gamma oscillations to propel fear extinction.

**Figure 5:**
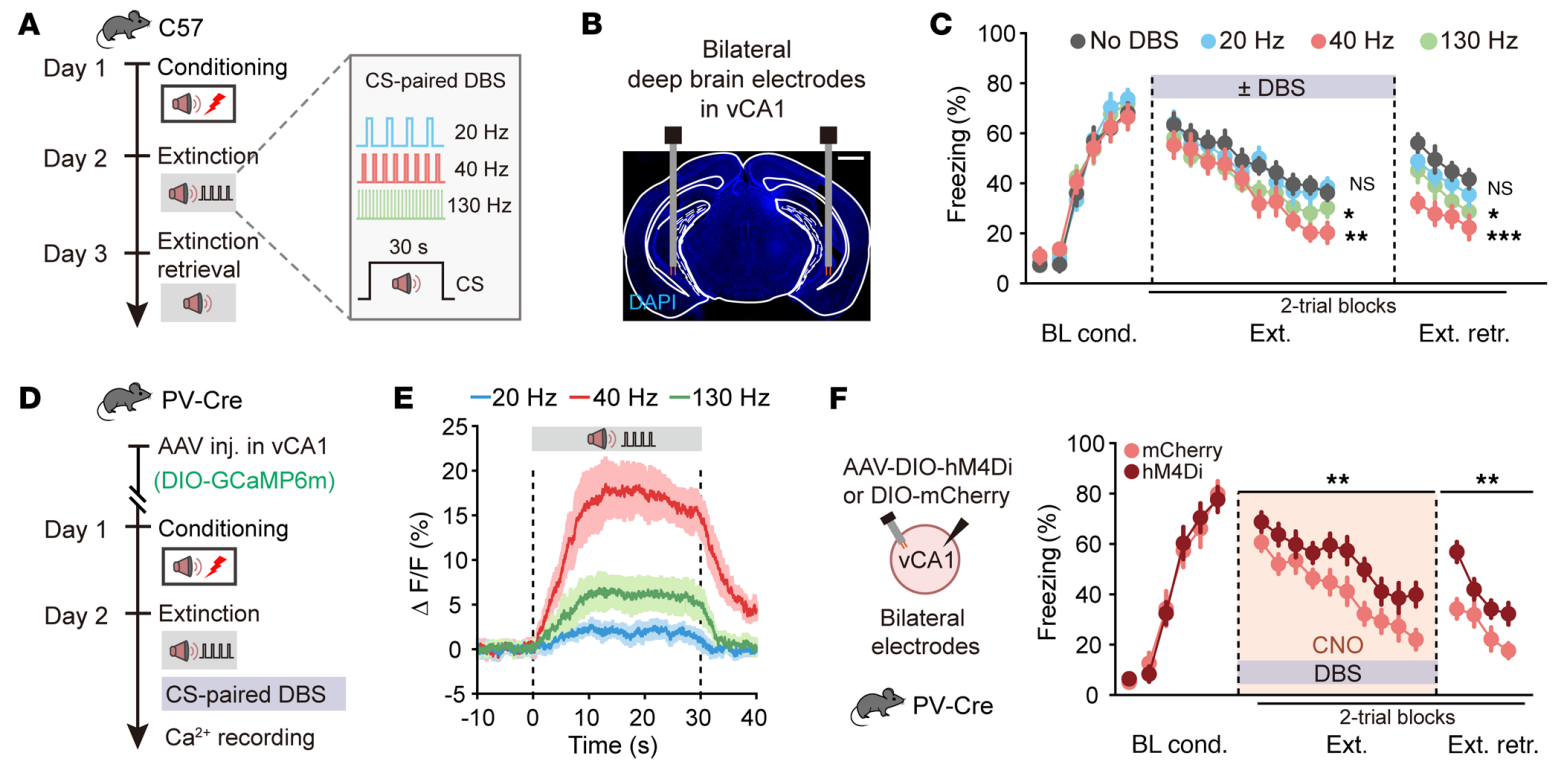
Long-term extinction promotion induced by low-gamma DBS depends on the activation of vCA1 PV-INs. (**A**) Schematics of experimental design. (**B**) Representative image showing electrode placements. Scale bar: 1 mm. (**C**) Time courses of freezing responses to the CS during fear conditioning, extinction training, and extinction retrieval. Statistics are as follows: Main effect of DBS frequency, conditioning, *F*_3,34_ = 0.3943, *P* = 0.7579. No DBS vs. 20 Hz DBS, extinction training, *F*_1,16_ = 0.3954, *P* = 0.5383; extinction retrieval, *F*_1,16_ = 2.126, *P* = 0.1642. No DBS vs. 40 Hz DBS, extinction training, *F*_1,16_ = 12.91, *P* = 0.0024; extinction retrieval, *F*_1,16_ = 24.91, *P* = 0.0001. No DBS vs. 130 Hz DBS, extinction training, *F*_1,16_ = 5.237, *P* = 0.0360; extinction retrieval, *F*_1,16_ = 5.192, *P* = 0.0368. No DBS group, *n* = 8 mice; 20 Hz DBS group, *n* = 10 mice; 40 Hz DBS group, *n* = 10 mice; 130 Hz DBS group, *n* = 10 mice. (**D**) Schematic of AAV injections and experimental design. (**E**) Average calcium signals in PV-INs during extinction training paired with DBS of different frequencies. 20 Hz group, *n* = 5 mice; 40 Hz group, *n* = 5 mice; 130 Hz group, *n* = 6 mice. (**F**) Effect of inhibiting vCA1 PV-INs on DBS-induced extinction promotion. Time courses of freezing responses to the CS during fear conditioning, extinction training, and extinction retrieval. Statistics are as follows: Main effect of AAV, conditioning, *F*_1,18_ = 0.0015, *P* = 0.9699; extinction training, *F*_1,18_ = 12.56, *P* = 0.0023; extinction retrieval, *F*_1,18_ = 14.80, *P* = 0.0012. *n* = 10 mice per group. Data are mean ± SEM. *P < 0.05, **P < 0.01, ***P < 0.001. Repeated-measures 2-way ANOVA (**C** and **F**).

Given the direct pathway from LEC to vCA1 governing fear extinction via low-gamma entrainment, we explored the efficacy of frequency-dependent DBS therapy targeting vCA1 in mice with fear memory. During extinction training, we paired the CS with DBS at different frequencies (20 Hz, 40 Hz, and 130 Hz), with 40 Hz falling within the low-gamma frequency range. Remarkably, mice exposed to 40 Hz DBS paired with the CS exhibited a significant reduction in freezing behavior, which persisted into extinction retrieval, compared with those with 20 Hz DBS or no DBS (Figure 5, A–C). These behavioral changes were specific to fear extinction, as there were no effects on exploratory behavior or baseline anxiety levels in the open field and elevated plus maze tests (Supplemental Figure 17, A–I). The vCA1 DBS did not affect fear conditioning or contextual fear retrieval or induce real-time place preference or aversion (Supplemental Figure 17, J–L). Mechanistically, 40 Hz DBS resulted in a much higher activation of PV-INs compared with other frequencies, correlating with behavioral outcomes (Figure 5, D and E, and Supplemental Figure 18). Chemogenetic inhibition of PV-INs specifically abolished the DBS effects on fear extinction (Figure 5F and Supplemental Figure 19). The response of other interneuron types to DBS was less pronounced, and their inhibition did not affect the effects of DBS on fear extinction (Supplemental Figure 20). Furthermore, optical stimulation of vCA1 PV-INs mimicked the effects of DBS on fear extinction (Supplemental Figure 21), underscoring the selective recruitment of PV-INs by 40 Hz DBS for enhanced extinction efficacy.

### PV-INs with high basal firing rate are preferentially recruited by low-gamma DBS in vCA1.

**Figure 6:**
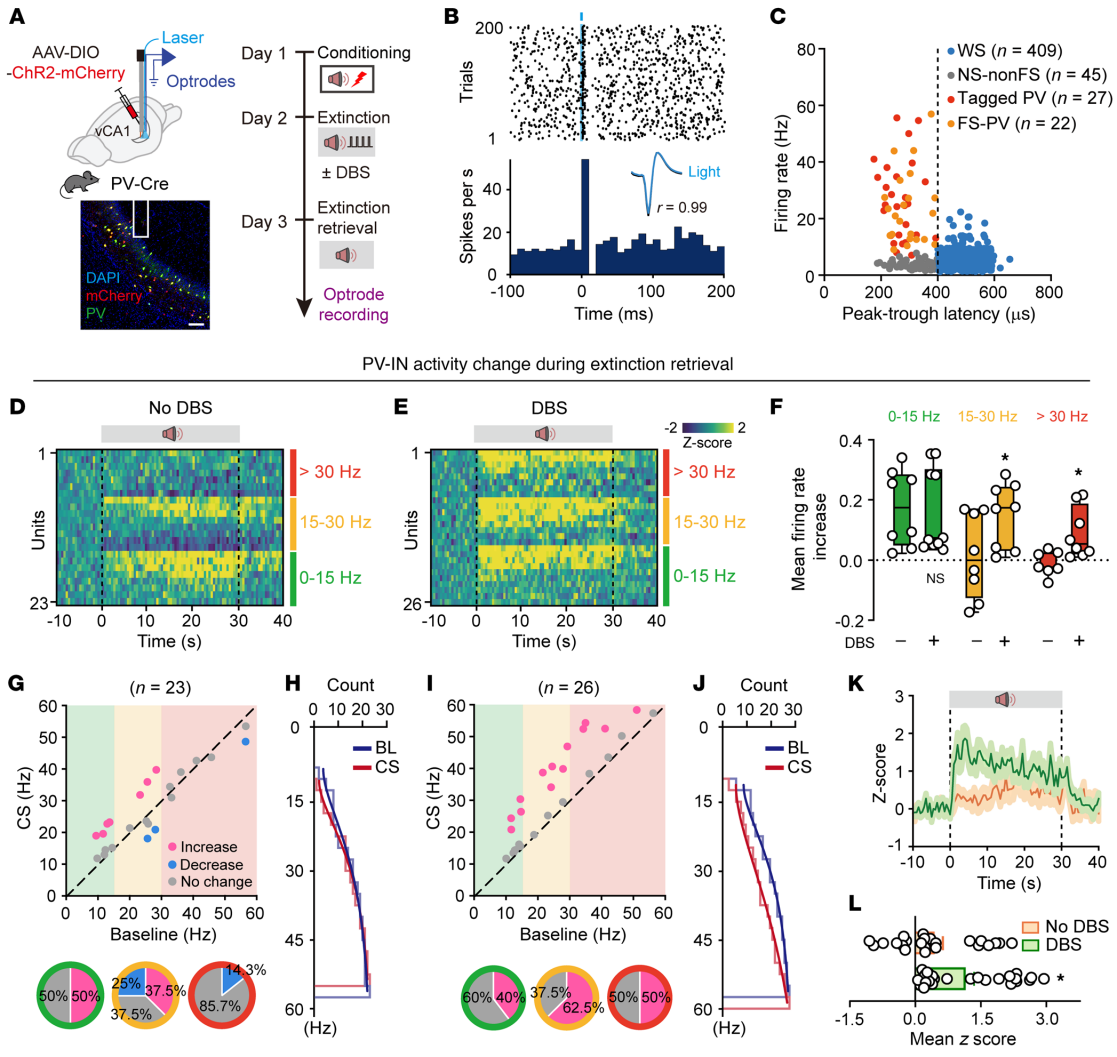
Extinction training paired with low-gamma DBS induces sustained activation of high-firing-rate vCA1 PV-INs during extinction retrieval. (**A**) Schematics of experimental design (top) and representative image of virus expression (bottom). Scale bar: 100 μm. (**B**) Raster plot (top) and peri-stimulus time histogram (bottom) of representative tagged PV-INs. In the inset, light-evoked spike waveforms (blue) were similar to spontaneous ones (black). Pearson’s correlation, *r* = 0.99. (**C**) Classification of recorded vCA1 neurons into wide spike (WS) putative pyramidal cells (blue circles), narrow spike–non-fast-spiking (NS-nonFS) (gray circles), tagged PV (red circles), and FS-PV (orange circles) based on peak-to-trough latency and baseline firing rate. (**D** and **E**) Heatmaps showing responses of PV-INs with different baseline firing rates during extinction retrieval. (**F**) Box plots of firing rate changes. The center line shows median, box edges indicate top and bottom quartiles, and whiskers extend to minimum and maximum values. Circles denote individual neurons. *P < 0.05. (**G** and **H**) Correlation of firing rate at baseline and during CS for individual PV-INs from no-DBS-manipulation mice. (**I** and **J**) The same as **G** and **H** for the correlation of firing rate during baseline (BL) and CS for individual PV-INs from DBS-manipulation mice. (**K** and **L**) *Z*-scored signal changes of PV-INs during extinction retrieval. Orange indicates no DBS manipulation during extinction training, and green indicates 40 Hz DBS manipulation during extinction training. Data are mean ± SEM. *P < 0.05. Unpaired Student’s *t* test (**F** and **L**).

To dissect vCA1 PV-IN firing dynamics during extinction retrieval with high precision, we conducted single-unit electrophysiological recordings. By opto-tagging PV-INs with AAV-DIO-ChR2-mCherry in PV-Cre mice and using an optrode above the vCA1 injection site (Figure 6A and Supplemental Figure 22A), we captured 503 well-isolated neurons, including 27 optogenetically tagged PV-INs, 409 wide spike neurons (putative pyramidal neurons), and 67 narrow spike neurons (putative interneurons) (Figure 6, B and C, and Supplemental Figure 22, B–E). Among the narrow spike population, we identified 49 putative PV-INs including the optogenetically tagged (*n* = 27) and fast-spiking putative (*n* = 22) interneurons, categorized by basal firing rates into high (>30 Hz), medium (15–30 Hz), and low (<15 Hz) groups.

During extinction retrieval without DBS, only a fraction of PV-INs (50% of neurons with 0- to 15-Hz basal firing rate and 37.5% of neurons with 15- to 30-Hz basal firing rate) exhibited increased firing rates in response to the CS, depending on their basal firing rates (Figure 6, D and G). However, when paired with DBS, all 3 groups of PV-INs, including high-firing-rate PV-INs, exhibited significant increases in firing rates in response to the CS (Figure 6, E, F, and I). The firing frequencies of PV-INs shifted toward higher values (Figure 6, H and J) during CS presentation in the presence of DBS, with a shorter latency (Figure 6, K and L). In contrast, putative pyramidal neurons in the DBS group showed an inverse redistribution in firing rate changes, including a larger proportion with decreased firing rates during CS presentation (Supplemental Figure 23), indicating increased inhibition. These results demonstrate that low-gamma DBS in the vCA1 region enhances the responsiveness of PV-INs, particularly those with higher basal firing rates, during extinction retrieval, while promoting inhibition of pyramidal neurons.

### Enduring activity of PV-INs due to low-gamma DBS suppresses fear-tagged neurons in vCA1.

**Figure 7:**
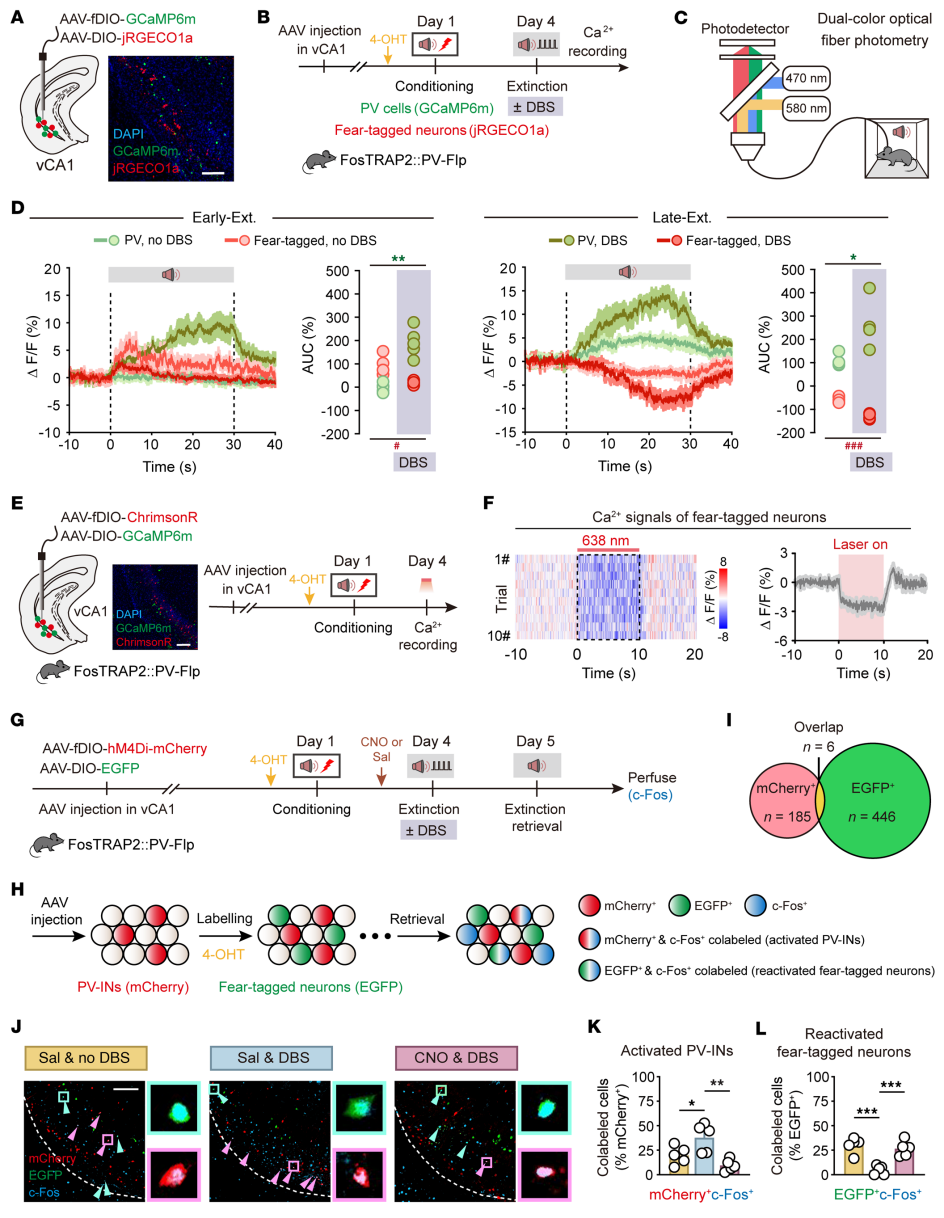
Extinction training paired with low-gamma DBS engages vCA1 PV-INs to suppress fear-tagged neurons. (**A**) Schematic of AAV injections and representative image of virus expression. Scale bar: 100 μm. (**B** and **C**) Schematics of experimental design. (**D**) Average calcium signals in PV-INs and fear-tagged neurons during Early-Ext. (left) and Late-Ext (right). *P < 0.05, **P < 0.01, PV-INs DBS vs. PV-INs no DBS, unpaired Student’s *t* test; ^#^*P* < 0.05, ^###^*P* < 0.001, fear-tagged neurons, DBS vs. no DBS. *n* = 5 mice per group. (**E**) Schematics of AAV injections and experimental design. Representative images of GCaMP6m expression in fear-tagged neurons and ChrimsonR expression in PV-INs in vCA1. Scale bar: 100 μm. (**F**) Left: Representative heatmap of fiber photometry recordings. Right: Averaged fluorescence decreased in response to optogenetic stimulation (*n* = 5 mice). (**G**) Schematic of AAV injections and experimental design. Administration of 4-OHT, 30 minutes before fear conditioning (i.p.), to FosTRAP2 PV-Flp mice was used to induce permanent expression of EGFP in neurons active around the time of the injection. (**H**) Genetic design to investigate fear-tagged neurons and neurons activated during extinction retrieval. Red circles represent PV-INs, green circles represent neurons labeled during conditioning, and blue circles represent neurons activated during memory retrieval. (**I**) Overlap between vCA1 PV-INs (mCherry^+^) and fear-tagged neurons (EGFP^+^). (**J**) Representative images of mCherry^+^ (red), EGFP^+^ (green), and c-Fos^+^ (blue) immunofluorescence in vCA1. Magenta arrowheads denote colabeled mCherry^+^c-Fos^+^ cells; cyan arrowheads denote colabeled EGFP^+^c-Fos^+^ cells. Circles represent enlarged images on the right. Scale bar: 100 μm. (**K** and **L**) The percentage of activated PV-INs (mCherry^+^c-Fos^+^) and reactivated fear-tagged neurons (EGFP^+^c-Fos^+^). Data are mean ± SEM. *P < 0.05, **P < 0.01, ***P < 0.001. Unpaired Student’s *t* test (**D**) and 1-way ANOVA with Tukey’s multiple-comparison test (**K** and **L**).

Given that low-gamma vCA1 DBS enhanced PV-IN activity, we postulated that the robust suppression of cued fear responses by DBS could arise from its ability to inhibit fear engrams. To test this, we used the targeted recombination in active populations (TRAP) strategy (42–44) in FosTRAP2 PV-Flp mice to tag fear engrams (fear-tagged neurons). We coadministered Flp-dependent AAV-fDIO-GCaMP6m and Cre-dependent AAV-DIO-jRGECO1a into vCA1, enabling simultaneous monitoring of PV-IN and fear-tagged neuron activities during extinction training paired with low-gamma DBS (Figure 7, A–C). The Ca^2+^ signals indicated that PV neuron activity was significantly elevated in the DBS group compared with the no-DBS group throughout the extinction process. Conversely, fear-tagged neurons showed increased activity only during the Early-Ext. phase, which was inhibited by DBS, and decreased activity during the Late-Ext. phase, with this reduction being more pronounced under DBS (Figure 7D). These patterns of activation for PV-INs and fear-tagged neurons persisted into the extinction retrieval phase (Supplemental Figure 24), reinforcing the lasting effects of vCA1 DBS on fear extinction.

To directly assess the influence of PV-INs on fear-tagged neurons, we introduced Flp-dependent AAV-fDIO-ChrimsonR and Cre-dependent AAV-DIO-GCaMP6m into vCA1 of FosTRAP2 PV-Flp mice. Activation of PV-INs via red light illumination in the vCA1 significantly reduced Ca^2+^ signals in fear-tagged neurons (Figure 7, E and F). Subsequent c-Fos analysis in these mice, following injection with AAV-fDIO-hM4Di-mCherry and AAV-DIO-EGFP into vCA1, showed that during extinction retrieval, the DBS group had an increased number of activated PV-INs (mCherry^+^c-Fos^+^) and a decreased number of reactivated fear-tagged neurons (EGFP^+^c-Fos^+^) compared with the no-DBS group (Figure 7, G–L). Moreover, chemogenetic suppression of PV-INs prevented the DBS-induced reduction in fear-tagged neurons, indicating that low-gamma vCA1 DBS activates PV-INs, which in turn suppresses fear-tagged neurons and diminishes cued fear responses (Figure 7, J–L). Notably, there was minimal overlap between mCherry^+^ and EGFP^+^ cells (Figure 7, I and J), suggesting that the proportion of vCA1 PV-INs integrated into fear-tagged neurons is negligible, and the PV-INs are preferentially engaged in fear extinction.

### Low-gamma DBS empowers LEC-vCA1 top-down feedforward inhibition pathway via PV-INs to suppress fear-tagged neurons.

**Figure 8:**
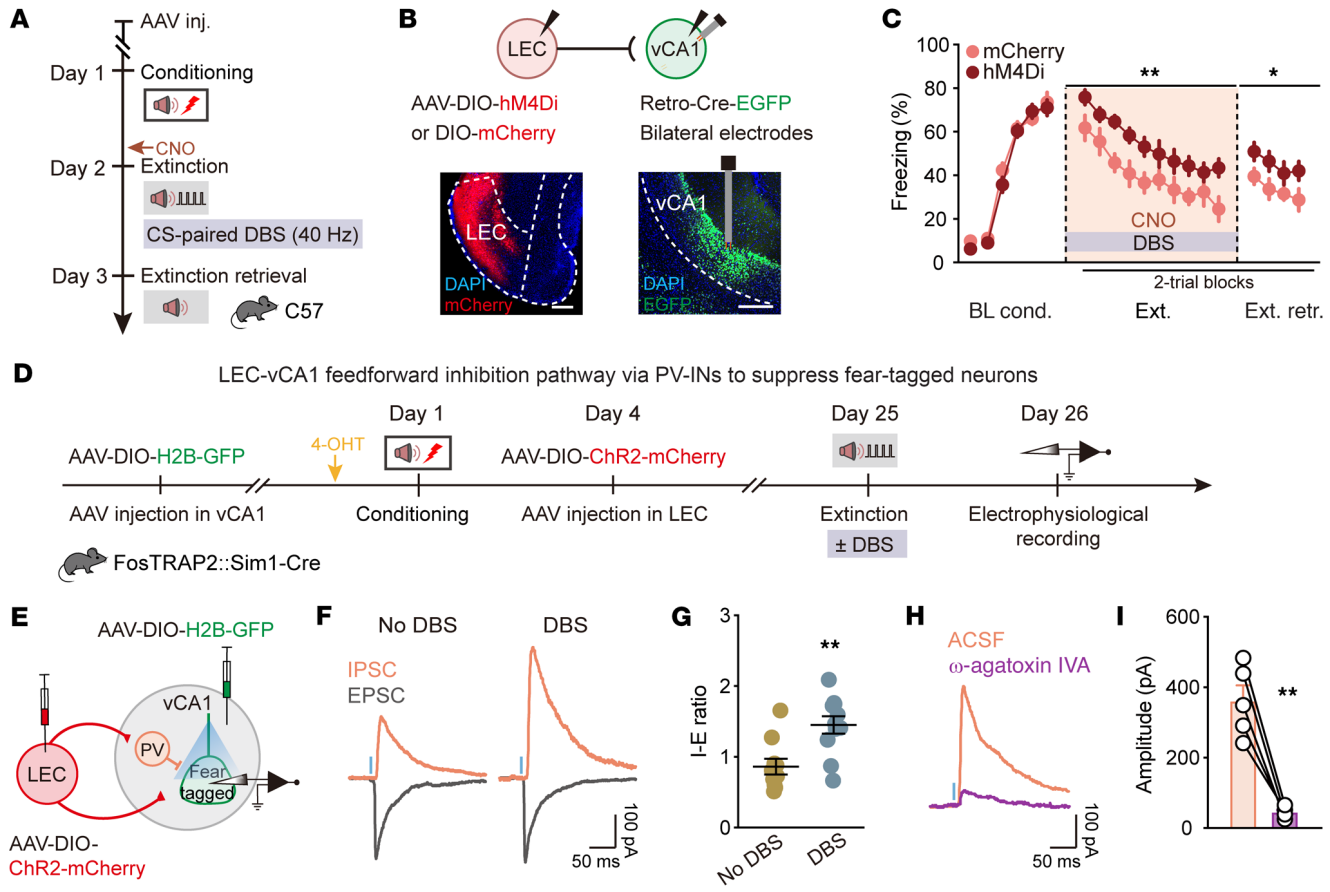
Low-gamma DBS strengthens the inputs from LEC driving PV IN–mediated feedforward inhibition in vCA1 and induces long-lasting suppression of fear-tagged neurons. (**A**) Schematic of experimental design. CS is paired with 40 Hz DBS during extinction training, and CNO was administered (i.p.) 30 minutes before extinction training. (**B**) Schematic of AAV injections (top) and representative images of virus expression (bottom). Scale bars: 200 μm. (**C**) Effect of inhibiting LEC-vCA1 projectors on DBS-induced extinction promotion. Time courses of freezing responses to the CS during fear conditioning, extinction training, and extinction retrieval sessions. Statistics are as follows: Main effect of AAV, conditioning, *F*_1,21_ = 0.4901, *P* = 0.4916; extinction training, *F*_1,21_ = 8.408, *P* = 0.0086; extinction retrieval, *F*_1,21_ = 7.556, *P* = 0.0120. mCherry group, *n* = 12 mice; hM4Di group, *n* = 11 mice. Data are mean ± SEM. *P < 0.05, **P < 0.01. (**D**) Schematic of AAV injections and experimental design. 4-OHT was administered 30 minutes before fear conditioning. (**E**) Experimental scheme for simultaneous recording of light-evoked EPSCs and IPSCs on vCA1 fear-tagged neurons. (**F**) Representative traces of EPSCs and IPSCs evoked by optogenetic stimulation of LEC fibers. (**G**) IPSC/EPSC peak ratios (No DBS, *n* = 10 cells; DBS, *n* = 11 cells). Data are mean ± SEM. **P < 0.01. (**H**) Representative traces showing that light-evoked IPSC amplitudes were reduced with application of 0.5 μM ω-agatoxin IVA. (**I**) Light-evoked IPSC amplitudes in vCA1 fear-tagged neurons with and without ω-agatoxin IVA (*n* = 5 cells). Data are mean ± SEM. **P < 0.01. Repeated-measures 2-way ANOVA (**C**), unpaired Student’s *t* test (**G**), and paired Student’s *t* test (**I**).

To investigate the role of the LEC-vCA1 pathway in mediating the effects of vCA1 DBS, we selectivity inhibited this pathway using chemogenetics during DBS. Inhibiting the LEC-vCA1 pathway (Figure 8, A–C), but not the MEC-vCA1 pathway (Supplemental Figure 25), attenuated the effects of vCA1 DBS, resulting in a higher fear response during extinction training and retrieval. The combination of vCA1 DBS and chemogenetic inhibition of the LEC-vCA1 pathway did not affect fear conditioning, contextual fear retrieval, or behavioral performance in the open field or induce conditioned place preference or aversion (Supplemental Figure 26). Additionally, optical stimulation of the LEC-vCA1 pathway, but not the MEC-vCA1 pathway, with low-gamma frequency replicated the effects of DBS on fear extinction (Supplemental Figure 27). This observation led us to hypothesize that DBS affects the inputs from LEC to vCA1 PV-INs, thereby suppressing fear-tagged neurons. To test this, we sequentially introduced AAV-DIO-H2B-GFP into vCA1 and AAV-DIO-ChR2 into LEC of FosTRAP2 Sim1-Cre mice (Figure 8, D and E). Initially, AAV-DIO-H2B-GFP was injected into vCA1, which allowed H2B-GFP expression in fear-tagged vCA1 cells following 4-hydroxytamoxifen (4-OHT) administration on day 1 before fear conditioning. Since Sim1-Cre is not expressed in vCA1, the expression of H2B-GFP is largely restricted to fear-tagged vCA1 neurons. After the response window for the TRAP system to 4-OHT (approximately 8 hours) (42, 44), we injected AAV-DIO-ChR2-mCherry into LEC on day 4. This approach ensures that ChR2-mCherry is specifically expressed in Sim1^+^ layer 2a cells in the LEC, while H2B-GFP marks fear-tagged neurons in vCA1. Photostimulation of LEC fibers induced monosynaptic excitatory postsynaptic currents (EPSCs) and delayed inhibitory postsynaptic currents (IPSCs) in the same vCA1 fear-tagged neuron, indicating that the LEC sends monosynaptic projections that form a strong feedforward inhibitory circuit to these cells. We observed a significant increase in the amplitude of light-evoked IPSCs in vCA1 fear-tagged neurons from the DBS group, compared with those from the no-DBS group, 1 day after fear extinction. The DBS group exhibited a marked increase in the IPSC/EPSC ratio (Figure 8, F and G). To confirm that vCA1 PV-INs are responsible for the feedforward inhibition within the LEC-vCA1 circuit, we blocked GABA release specifically from PV-INs using ω-agatoxin IVA, a selective antagonist for P/Q-type Ca^2+^ channels (45). Following the application of ω-agatoxin IVA, the IPSC amplitude showed a significant decrease (Figure 8, H and I), confirming that PV-INs mediate the feedforward inhibition driven by LEC Sim1^+^ layer 2a fan cells onto vCA1 fear-tagged neurons. Overall, our findings suggest that low-gamma DBS manipulation strongly activates inputs from LEC that drive PV-IN–mediated feedforward inhibition in vCA1, leading to the long-term suppression of fear-tagged neurons.

### Low-gamma tACS targeting LEC enhances fear extinction.

**Figure 9:**
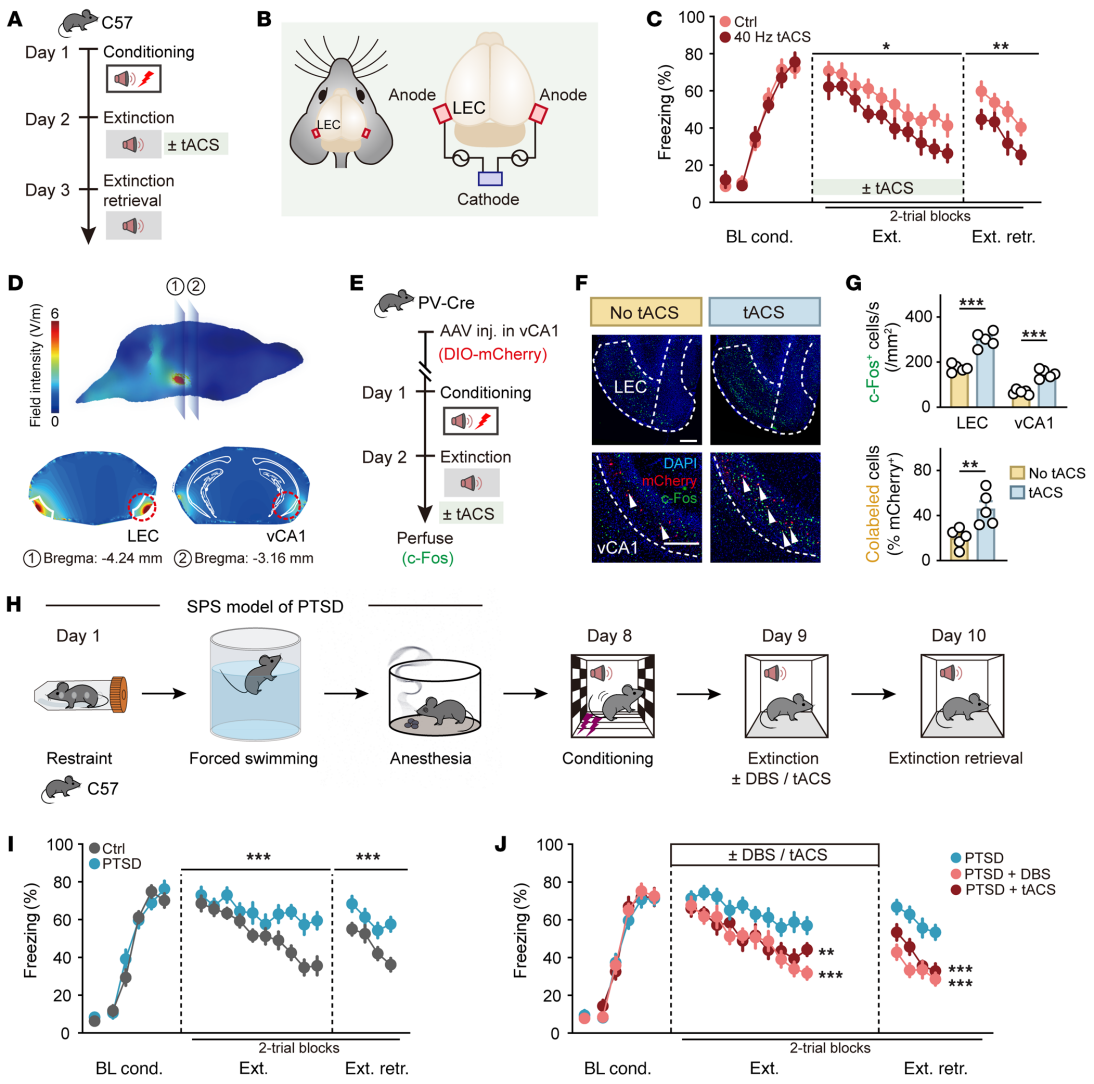
Low-gamma stimulation of LEC vCA1 circuit enhances fear extinction, even under more traumatic conditions. (**A** and **E**) Schematics of experimental design. (**B**) Schematic diagram of stimulus configuration. (**C**) Time courses of freezing responses to the CS. Statistics are as follows: Main effect of tACS, conditioning, *F*_1,14_ = 0.0331, *P* = 0.8582; extinction training, *F*_1,14_ = 8.055, *P* = 0.0132; extinction retrieval, *F*_1,14_ = 15.87, *P* = 0.0014. *n* = 8 mice per group. (**D**) Predicted current density map at the surface of the brain during tACS (top) and slice images of the distribution showing peak current densities during tACS (bottom). (**F**) Representative images of mCherry^+^ (red) and c-Fos^+^ (green) immunofluorescence. White arrowheads denote colabeled cells. Scale bars: 200 μm. (**G**) Quantification for **F**. *n* = 5 mice per group. (**H**) Schematic illustration of single prolonged stress (SPS) and the fear conditioning paradigm. (**I** and **J**) Time courses of freezing responses to the CS. Statistics are as follows: (**I**) Main effect of treatment, conditioning, *F*_1,16_ = 0.2782, *P* = 0.6051; extinction training, *F*_1,16_ = 22.92, *P* = 0.0002; extinction retrieval, *F*_1,16_ = 38.08, *P* < 0.0001. *n* = 9 mice per group. (**J**) PTSD vs. PTSD + DBS, conditioning, *F*_1,16_ = 0.5860, *P* = 0.4551; extinction training, *F*_1,16_ = 16.79, *P* = 0.0008; extinction retrieval, *F*_1,16_ = 70.31, *P* < 0.0001. PTSD vs. PTSD + tACS, conditioning, *F*_1,15_ = 0.5624, *P* = 0.4649; extinction training, *F*_1,15_ = 14.42, *P* = 0.0018; extinction retrieval, *F*_1,15_ = 30.04, *P* < 0.0001. PTSD group, *n* = 9 mice; PTSD + DBS group, *n* = 9 mice; PTSD + tACS group, *n* = 8 mice. Data are mean ± SEM. *P < 0.05, **P < 0.01, ***P < 0.001. Repeated-measures 2-way ANOVA (**C**, **I**, and **J**) and unpaired Student’s *t* test (**G**).

To explore the clinical potential of noninvasive neuromodulation, we investigated the effects of tACS (39) on the LEC-vCA1 pathway. We aimed to use electrical signals delivered via tACS to modulate LEC activity, thereby influencing vCA1 similarly to vCA1 DBS and promoting fear extinction. Bilateral stimulation electrodes (anodes) were implanted over the LEC regions, with a cathode placed on the neck skin of the mice. The mice were divided into 2 groups: one receiving tACS (200 μA, 40 Hz, paired with the CS) and a control group without tACS (Figure 9, A and B). The tACS group demonstrated accelerated extinction compared with the no-tACS group, with this effect persisting into the extinction retrieval session (Figure 9C). Notably, LEC tACS did not affect fear conditioning, contextual fear retrieval, or behavioral performance in the open field or induce real-time place preference or aversion (Supplemental Figure 28, A–I).

To further investigate the effects of low-gamma (40 Hz) tACS on vCA1, we used computational modeling to assess the electric field generated during tACS. The predicted current density map at the brain surface and specific slice views indicated an increase in current density within vCA1 during tACS targeting LEC (Figure 9D). We also analyzed c-Fos expression levels to quantify activity patterns in the presence and absence of the 40 Hz tACS (Figure 9E). The tACS group showed significant increases in the number of c-Fos^+^ cells in both the LEC and vCA1 regions compared with the no-tACS group. Additionally, there was a substantial increase in the number of PV^+^c-Fos^+^ cells in vCA1 (Figure 9, F and G). These results suggest that noninvasive LEC tACS promotes neural communication between the cortex and HPC by recruiting vCA1 PV-INs, sharing similar cellular mechanisms with vCA1 DBS.

### Low-gamma LEC tACS and vCA1 DBS effectively reduce persistent fear in a mouse model of PTSD.

Finally, given that anxiety disorders and PTSD are characterized by persistent fear and difficulties in extinction learning (1, 2), we investigated whether low-gamma LEC tACS or vCA1 DBS could mitigate these symptoms in a PTSD mouse model. The model was induced by single prolonged stress (46–50), consisting of 3 consecutive stressors: restraint, forced swimming, and anesthesia (Figure 9H). This PTSD model, known for its resistance to fear extinction, displayed persistent fear memory without significant differences in the initial fear learning curve in comparison with control mice (Figure 9I). Notably, the application of either LEC tACS or vCA1 DBS significantly facilitated the extinction of cued fear in the PTSD mice (Figure 9J). Moreover, neither vCA1 DBS nor LEC tACS affected behavioral performance in the open field or induced real-time place preference or aversion (Supplemental Figure 28, J–M). These results highlight the potential of both invasive and noninvasive neuromodulation approaches, which target low-gamma entrainment of the entorhinal-hippocampal circuit, to enhance extinction processes and alleviate traumatic memory retention even in severe conditions.

## Discussion

**Figure 10:**
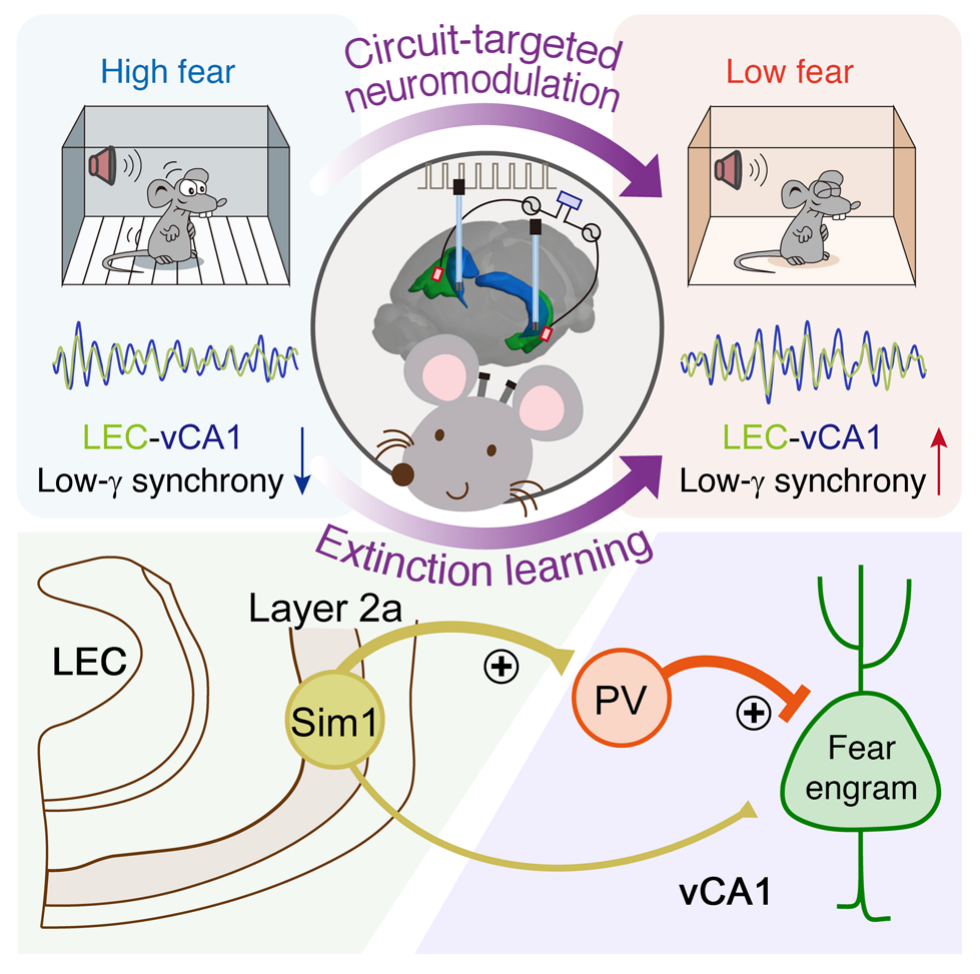
Scheme for a direct LEC-vCA1 projection pathway and the role of low-gamma oscillations and inter-regional entrainment in driving fear extinction, orchestrated by vCA1 PV-INs. This cortical-subcortical motif can be therapeutically targeted through either vCA1 DBS or LEC tACS to enhance feedforward inhibition of fear-tagged neurons, thereby augmenting extinction to remove traumatic memories.

Gaining insights into the neurological mechanisms underlying fear extinction holds substantial promise for psychotherapy, particularly for addressing the challenging issue of PTSD. Our current study unveils a direct projection pathway from the LEC to the vCA1, which is necessary and sufficient for implementing fear extinction. We unravel that fear extinction relies on low-gamma oscillations between the LEC and vCA1 at the circuit level coordinated by vCA1 PV-INs. Direct projections from LEC layer 2a fan cells to vCA1 PV-INs are distinct from indirect projections to the dorsal HPC. Furthermore, we found that exogenous low-gamma vCA1 DBS not only enhances fear extinction but also exerts enduring benefits. This remarkable efficacy is primarily attributed to the activation of high-firing PV-INs and the persistent suppression of fear-tagged neurons, leading to a sustained reduction in fear responses. In our exploration of potential treatments for fear-related disorders like PTSD, we found that noninvasive low-gamma LEC tACS effectively reduces enduring fear when combined with fear extinction training. This positive outcome holds even in a mouse model of PTSD with the most extinction-resilient form of fear memory, rationalizing its practical utility. Together, our study uncovers a top-down structural motif along the cortical-subcortical axis, in which inter-regional synchronization of low-gamma oscillations between LEC and vCA1 prompts extinction of enduring fear memory. These findings not only define a circuitry and mechanistic basis of fear extinction but also present a proof of principle for using FDA-approved invasive and noninvasive approaches to stimulate this pathway for removing traumatic memories with significant efficacy and persistence (Figure 10).

Contrary to the extensively studied dorsal hippocampal–entorhinal network, which supports both spatial navigation and associative memory (16–19, 51, 52), the connectivity, activity, and consequent behavioral implications of the ventral hippocampal–entorhinal network remain largely unexplored. A circuit mapping study has unveiled significant variations in input proportions and distributions between dorsal and ventral hippocampal CA1 pyramidal neurons, including distinct input patterns from the EC (53). Notably, there are instances in which projections from EC neurons expressing corticotropin-releasing factor (CRF) directly target the vCA1, influencing behaviors of mice that respond to human experimenters’ sex and modulating the animals’ neural responses to ketamine (54). Here, we identify a direct projection from LEC layer 2a fan cells to vCA1 PV-INs, which controls fear extinction learning. Both populations of neurons in the projections, including LEC layer 2a fan cells and vCA1 PV-INs, are significantly activated by fear extinction learning. Notably, the LEC-vCA1 projections are necessary for the low-gamma-band oscillatory firing in each area, along with inter-regional low-gamma synchronization in response to fear extinction learning. Behaviorally, the specific activation or inhibition of this projection demonstrated a bidirectional influence on fear extinction. More strikingly, this projection was amenable to alteration through DBS and noninvasive tACS neuromodulation approaches. Among the projections from LEC layer 2a fan cells to various cell types in vCA1, isolation of this pivotal connection from LEC layer 2a fan cells to vCA1 PV-INs opens up an exciting avenue to decode the neural network mechanism of fear extinction. In conjunction with the well-known role of dorsal hippocampal–entorhinal circuits in spatial navigation and associative memory, our discovery of a monosynaptic pathway from LEC to the vCA1 region, characterized by a unique projection pattern and specificity in fear extinction, exemplifies the organization of parallel structural and functional motifs for segregating single memory trace with diverse contents.

The LEC-vCA1 pathway, pivotal to fear extinction, is likely part of cognitive motif ensembles for memory processing. Unlike the established extinction circuits (3, 23, 55, 56) that collectively appear to effectively inhibit the expression of conditioned fear behaviors, the LEC integrates diverse sensory information (15, 19, 57–59), aided by dopaminergic innervation, to construct a cognitive map of abstract task rules (14). Fear extinction as a more abstract form of inhibitory learning (60) requires a dopaminergic switch for transitioning from fear to safety (61, 62). However, the contribution of LEC dopamine signals to the LEC-vCA1 motif remains open for future investigation. Thus, parallel cortical-subcortical motifs effectively process intricate contextual and sensory cues, with the LEC-vCA1 pathway being the key handle for implementing extinction of conditioned fear behaviors.

Notably, there exists an indirect pathway from LEC Sim1^+^ layer 2a fan cells to vCA1 via vDG and vCA3, potentially mediating some effects on fear extinction. Our optogenetic inhibition experiments revealed that reducing the activation of projections from LEC Sim1^+^ layer 2a fan cells to vCA1, vCA3, and vDG during the fear extinction phase significantly decreased activation in vCA1 PV-INs, but not in vCA3 or vDG. Consistently, recordings of Ca^2+^ signals in the vCA1, vCA3, and vDG terminals from LEC Sim1^+^ layer 2a fan cells during the fear extinction phase showed significant activation only in the projections to vCA1 during extinction training and retrieval. Moreover, optogenetic inhibition of projections from LEC Sim1^+^ layer 2a fan cells to vCA1, vCA3, and vDG demonstrated that only the inhibition of the direct projection to vCA1 significantly attenuated fear extinction, underscoring the critical role of this direct pathway in the fear extinction process. Therefore, while we acknowledge the potential involvement of the indirect pathway, our findings highlight the dominant role of the direct pathway from LEC Sim1^+^ layer 2a fan cells to vCA1 PV-INs in mediating fear extinction, warranting further investigation into the indirect pathway’s contributions.

It is plausible that vCA1 PV-INs, within the top-down motif, are selectively and progressively recruited during fear extinction learning, facilitating synchronization of cortical and subcortical networks for fear extinction. This aligns with the concept that fear extinction involves inhibitory learning mechanisms directed against the original fear memory (63). In this study, we present compelling evidence supporting the existence of an extinction-initiated memory trace, with vCA1 PV-INs playing a causal role in suppressing fear-tagged neurons at the network level. Notably, vCA1 PV-INs exhibit significant multifaceted adaptations upon fear extinction. First, there is a gradual increase in neuronal activity throughout the extinction learning process, as indicated by a progressive rise in cue-evoked Ca^2+^ signals. This adaptation reflects an increasing responsiveness of vCA1 PV-INs to the CS as extinction process advances. Second, a post-learning (extinction) adaptation is observed, marked by elevated c-Fos expression in vCA1 PV-INs following extinction learning compared with control conditions in a homecage setting. This suggests a lasting adaptation beyond the immediate learning phase, possibly linked to the consolidation or retention of extinction memory. Lastly, during extinction retrieval, vCA1 PV-INs display persistent plasticity, characterized by enhanced neuronal firing in response to CS presentation and a notable increase in the proportion of high-firing-rate PV-INs, particularly after vCA1 DBS modulation. This indicates long-term changes in the excitability and firing patterns of vCA1 PV-INs, crucial for the retrieval of the extinction memory. While the exact molecular mechanisms underlying these vCA1 PV-IN adaptations remain to be fully understood, targeting these adaptations holds promise for developing treatments for fear-related disorders. It is hypothesized that vCA1 pyramidal neurons, as the final component of the cortical-subcortical motif for fear extinction, may shift their firing toward lower frequencies and more synchronous patterns because of the adaptations in vCA1 PV-INs resulting from extinction training and vCA1 DBS. Overall, vCA1 PV-INs dynamically adjust their activity throughout the fear extinction process, thereby synchronizing neuronal activity within the cortical-subcortical motif for learning to extinguish fear memory.

Translating our circuit findings, we established two independent neuromodulation approaches, vCA1 DBS and LEC tACS, to enhance fear extinction, providing potential interventions for PTSD and other fear-related disorders. Both approaches effectively mitigated extinction resistance in a PTSD mouse model, a result attributed to activation of high-firing-rate vCA1 PV-INs and sustaining of fear-tagged neuron suppression, resulting in lasting fear reduction. Building on established therapeutic approaches for Parkinson’s disease using DBS (64, 65) and promising results in noninvasive brain stimulation methods, such as tACS, for various conditions (39, 66), our findings advocate applying neuromodulation techniques to address fear-related disorders, including PTSD. Because the neocortex is the most accessible with these neuromodulation technologies, our identification of adaptable motifs along the cortical-subcortical axis to boost fear extinction exemplifies the potential to advance the treatment options for individuals grappling with debilitating psychiatric and neurodegenerative conditions.

In conclusion, our study unveils the significance of the direct LEC-vCA1 projection and the role of low-gamma oscillations and inter-regional entrainment in driving fear extinction, orchestrated by vCA1 PV-INs. By validating the efficacy of vCA1 DBS and LEC tACS, we introduce effective neuromodulation techniques to augment fear extinction, presenting promising interventions for PTSD and related disorders. These findings not only deepen our comprehension of psychotherapeutic approaches but also pave the way for innovative treatments in the realm of fear-related conditions. Our findings serve as a proof of principle for advancing therapies for memory diseases and neuropsychiatric disorders by precisely targeting accessible top-down cortical motifs in a pathway-specific manner with cell type–specific effects.

## Methods

Detailed information on materials and methods is provided in Supplemental Methods.

### Sex as a biological variable.

Our study examined male mice to investigate PTSD mechanisms because of their stable hormonal cycles, which reduce variability and allow for more consistent data interpretation. Male and female rodents can exhibit different stress responses, likely influenced by sex hormones. By focusing on male mice initially, we establish a clear baseline understanding of PTSD neural circuits without hormonal fluctuations. Although there are sex-specific differences, the core pathways involved in fear extinction and neural plasticity are conserved across sexes, making our findings relevant to both. Future studies will include female mice to ensure comprehensive insights.

### Animals.

The following animals were used in this study: C57BL/6J mice (Shanghai Laboratory Animal Center at the Chinese Academy of Sciences, Shanghai, China), Fos^2A-iCreER^ (TRAP2) mice (stock 030323, The Jackson Laboratory), PV-Cre mice (stock 017320, The Jackson Laboratory), PV-Flp mice (stock 022730, The Jackson Laboratory), SST-Cre mice (stock 013044, The Jackson Laboratory), VIP-Cre mice (stock 010908, The Jackson Laboratory), Sim1-Cre mice (stock 006395, The Jackson Laboratory), and lox-stop-lox-H2B-GFP (H2B-GFP^flox^) reporter mice (67). All mice were group-housed on a 12-hour light/12-hour dark cycle with rodent chow and water ad libitum. Adult male mice (8–12 weeks old) were used for all experiments.

### Statistics.

Detailed statistical analyses were performed using MATLAB (MathWorks) and GraphPad Prism. The data were collected and processed randomly. All behavioral tests and analyses were blindly conducted. Data distributions were tested for normality, and variance equality among groups was assessed using Levene’s test. Data are mean ± SEM unless otherwise indicated. Statistical comparisons were performed using 2-tailed unpaired or paired Student’s *t* test and 2-tailed 1-sample *t* test as well as 1-way or 2-way repeated-measures ANOVA, where appropriate. For non-parametric data sets, Wilcoxon’s signed-rank test was used to determine significance. For post hoc analysis, Tukey’s, Bonferroni’s, or Šidák’s multiple-comparison test was used for multiple comparisons. Significance is mainly displayed as *P < 0.05, **P < 0.01, ***P < 0.001; NS denotes no significant difference, which is not typically indicated except for emphasis.

### Study approval.

All animal care and experimental procedures were approved by the Animal Ethics Committee of Shanghai Jiao Tong University School of Medicine and by the Institutional Animal Care and Use Committee (Department of Laboratory Animal Science, Shanghai Jiao Tong University School of Medicine; policy DLAS-MP-ANIM.01–05).

### Data and code availability.

All data needed to evaluate the conclusions of this study are present in the main paper and/or the supplemental material. Source data for this study are also available in the supplemental Supporting Data Values file. All data used to generate the figure panels and the code built on the FieldTrip toolbox (68) for weighted phase lag index analysis can be found at Zenodo (https://doi.org/10.5281/zenodo.13268936). Any additional information required for reanalyzing the reported data is available upon request.

## Author contributions

ZJL, XG, TFY, WGL, and TLX conceived the project, designed the experiments, and interpreted the results. ZJL and XG performed the majority of behavioral experiments, animal surgery, immunohistochemistry, and data analysis. YJW, QW, and XYZ assisted with some of the behavioral experiments and conducted viral injections. WKG assisted with DBS and tACS experiments. MW and QL did computational modeling. XG, YJW, and XRW performed slice recording and data analysis. ZJL, XG, MXZ, LYW, WGL, and TLX wrote the manuscript with contributions from all authors. All authors read and approved the final manuscript.

## Supplementary Material

Supplemental data

Supporting data values

## Version 1 09/24/2024

In-Press Preview

## Version 2 11/15/2024

Electronic publication
