# Supplementary material for: Stimulation of an entorhinal-hippocampal extinction circuit facilitates fear extinction in a post-traumatic stress disorder model
Source: J Clin Invest. 2024 Sep 24;134(22):e181095. doi: 10.1172/JCI181095 (PMC11563685; doi:10.1172/JCI181095)
Supplement: Supplemental data [file jci-134-181095-s129.pdf]

Supplemental Material for

Stimulation of an entorhinal-hippocampal extinction circuit facilitates fear extinction in a post-traumatic stress disorder model

Ze-Jie Lin[1,2,†], Xue Gu[2,3,†], Wan-Kun Gong[4], Mo Wang[5], Yan-Jiao Wu[1,2], Qi Wang[1,2], Xin-Rong Wu[1,2], Xin-Yu Zhao[1,2], Michael X. Zhu[6], Lu-Yang Wang[7,8], Quanying Liu[5], Ti-Fei Yuan[4,*], Wei-Guang Li[2,9,10,*], Tian-Le Xu[1,2,11,*]

The file includes:

Supplemental Methods

Supplemental Figures 1 to 28

Supplemental References

Supplemental Methods

Virus constructs. The following viruses were used: rAAV2/9-EF1α-DIO-eNpHR3.0-mCherry-WPRE-hGH polyA, rAAV-EF1α-DIO-RVG-WPRE-hGH polyA, rAAV-EF1α-DIO-H2B-EGFP-T2A-TVA-WPRE-hGH polyA, RV-EnvA-DG-DsRed, rAAV-nEf1α-fDIO-RVG-WPRE-hGH polyA, rAAV-nEF1α-fDIO-EGFP-T2A-TVA-WPRE-hGH polyA, rAAV2/9-EF1a-DIO-BFP-Flag-WPRE-bGH polyA, rAAV2/9-CaMKIIa-hChR2(E123T/T159C)-mCherry-WPRE-hGH polyA, rAAV2/9-CaMKIIa-mCherry-WPRE-hGH polyA, rAAV2/9-EF1α-DIO-hM4D(Gi)-mCherry-WPREs, rAAV2/9-EF1α-DIO-mCherry-WPRE-hGH polyA, rAAV2/9-EF1a-Con Fon-GCaMp6s-WPRE-hGH polyA, rAAV2/R-hSyn-FLP-WPRE-hGH polyA, rAAV2/9-EF1a-DIO-hM3D(Gq)-EGFP-WPREs, rAAV2/9-EF1a-DIO-EGFP-WPRE-hGH polyA, AAV2/1-hSyn-FLP-WPRE-hGH polyA, rAAV2/9-hSyn-Con Fon-hM4D(Gi)-EGFP-WPRE-hGH polyA, rAAV2/9-hSyn-Con/Fon-EYFP-WPRE-hGH polyA, rAAV2/R-hSyn-CRE-EGFP-WPRE-hGH polyA, rAAV2/R-hSyn-CRE-WPRE-hGH polyA, rAAV2/9-nEF1a-fDIO-GCaMp6m-WPRE-hGH polyA, rAAV2/9-EF1a-jRGECO1a-WPREs, rAAV2/9-EF1α-DIO-hChR2(E123T/T159C)-mCherry-WPRE-hGH polyA and rAAV2/9-EF1a-DIO-EGFP-WPRE-hGH polyA (all purchased from Brain VTA, Wuhan, China); AAV2/9-hSyn-DIO-GCaMP6m-WPRE-pA, AAV2/9-hEF1a-fDIO-hM4D(Gi)-mCherry-ER2-WPRE-pA and AAV2/9-hEF1a-fDIO-ChrimsonR-mCherry-ER2-WPRE-pA (from Taitool Bioscience, Shanghai, China). All viruses were stored in aliquots at –80 °C until use. The viral titers for injection were $>10^{12}$ viral particles per ml.

Stereotaxic surgery. Mice at 6–7 weeks old were anesthetized with 1% sodium pentobarbital via a single intraperitoneal injection per mouse (10 ml per kg body weight), after which each mouse was mounted in a stereotactic frame with non-rupture ear bars (RWD Life Science). After making an

incision to the midline of the scalp, small craniotomies were performed using a microdrill with 0.5-mm burrs. For LFP recording, tungsten electrodes (Microprobes) were inserted into following coordinates (posterior to Bregma, AP; lateral to the midline, ML; below the Bregma, DV; in mm): LEC: AP, –4.16 mm; ML, ±3.90 mm; DV, –4.60 mm; MEC: AP, –4.46 mm; ML, ±3.0 mm; DV, –4.20 mm; vCA1: AP, –3.20 mm; ML, ±3.08 mm; DV, –4.0 mm.

For AAV injection, virus solutions were loaded into the tips of pipettes (Sutter Glass pipettes) and injected at the following coordinates: LEC: AP, –4.16 mm; ML, ±3.90 mm; DV, –4.60 mm; MEC: AP, –4.46 mm; ML, ±3.0 mm; DV, –4.20 mm; vCA1: AP, –3.20 mm; ML, ±3.08 mm; DV, –4.0 mm. After injection, the pipette was left in place for an additional 10 min to allow the injectant to diffuse adequately.

For retrograde monosynaptic tracing, a 1:1 volume mixture of AAV-EF1α-DIO-RVG and AAV-EF1α-DIO-H2B-EGFP-TVA (200-300 nl) was injected into the vCA1 of PV-Cre mice. A 1:1 volume mixture of AAV-EF1α-fDIO-RVG and AAV-EF1α-fDIO-H2B-EGFP-TVA (200-300 nl) was injected into the vCA1 of PV-Flp mice. Two weeks later, RV-ENVA-dG-DsRed (200-300 nl) was injected into the same location. The histology experiments were performed one week after rabies virus injection. For fiber photometry and optogenetic experiments, ceramic fiber optic cannulas (200 μm in diameter, 0.37 numerical aperture (NA), Hangzhou Newdoon Technology) were implanted above the vCA1 (AP, –3.20 mm; ML, ±3.08 mm; DV, –3.9 mm).

Fear conditioning, extinction, and memory retrieval. All auditory fear conditioning, extinction, and memory retrieval procedures were performed using the Ugo Basile Fear Conditioning System

(UGO BASILE srl). Briefly, mice were first handled and habituated to the conditioning chamber for three successive days. The conditioning chambers (17 cm × 17 cm × 25 cm) were equipped with stainless-steel shocking grids and connected to a precision-feedback current-regulated shocker. During fear conditioning, the chamber walls were covered with black-and-white checkered wallpaper, and the chambers were cleaned with 75% ethanol (context A). On day 1, mice were conditioned individually in context A with five pure tones (CS; 4 kHz, 76 dB, 30 s each) delivered at variable intervals (20–180 s). Each tone was co-terminated with a foot shock (US; 0.75 mA, 2 s each). ANY-maze software (Stoelting Co.) was used to automatically control the delivery of tones and foot shocks. Conditioned mice were returned to their homecages 30 s after the end of the last tone, and the cage was cleaned with 75% ethanol for each mouse. For extinction learning, mice conditioned on day 1 were presented with 20 CS presentations (4 kHz, 76 dB, 30 s each) without foot shock in context B (gray floor box) on day 2. On day 3, mice received eight CS-alone (30 s each) presentations in the extinction context (context B) for extinction retrieval. In experiments presented in Supplemental Figures 1, 4, 10, and 12, mice were habituated to the conditioning chamber on day 0. On day 1, mice were conditioned to the pure tone (CS; 4 kHz, 76 dB, 30 s each) individually in context A. The following day, mice were re-exposed to context A for 3 min for contextual fear retrieval. On day 3, mice were placed in context B with five presentations of white noise (CS-; 76 dB, 30 s each) in the absence of the footshock. On day 4, mice were presented with 20 CS presentations (30 s each) in the extinction context (context B) without foot shock for extinction learning. On day 5, mice received eight CS presentations (30 s each) in the extinction context (context B) for extinction retrieval. The movement of the mouse in the chamber was recorded using a near-infrared camera and analyzed in real-time with ANY-maze software. A fear response was operationally defined as measurable behavioral freezing (more than 1-s cessation of movement), which was automatically scored and analyzed by ANY-maze software

(version 7.2, Stoelting Co., USA).

Open field test. The open field test was conducted to measure general locomotor activity. The mice with DBS or tACS electrodes were connected to the stimulator by means of a flexible cable and placed in an open field chamber (40-cm length, 40-cm width, 30-cm height). The open field chamber was divided into a center zone (center, 20 × 20 cm) and an outer zone (periphery). The movement of the mice was recorded and analyzed by using a video-tracking system (EthoVision 15.0, Noldus). In the experiments with DBS or tACS, the open field test consisted of an 18-minute session in which there were six alternating 3-minute epochs (OFF-ON epochs). The time spent in the center zone and the total distance and velocity traveled in the whole open field arena were measured over 18 min. In the experiments with chemogenetic manipulation, the open field test consisted of a 10-minute session. The time spent in the center zone and the total distance and velocity traveled in the whole open field arena were measured over 10 min.

Elevated plus maze test. The elevated plus maze was made of grey plastic and consisted of two closed arms (30 × 5 cm), two open arms (30 × 5 cm) and a central platform (5 × 5 cm). The maze was elevated 30 cm from the ground. The test mouse was placed in the center of the crossed maze, and the locomotion of the animal was recorded with a video-tracking system (EthoVision 15.0, Noldus) for 9 min. Each session was divided into three alternating 3-minute epochs: stimulation off, stimulation on, and stimulation off (OFF-ON epochs). The time spent in the open arms and that spent in the closed arms during the 9 mins period were quantified by EthoVision software.

Real-time place preference/aversion test. A two-compartment real-time place preference test was

conducted to assess whether acute stimulation, either vCA1 DBS or LEC tACS, induces behavioral consequences to generate emotional valence. This protocol was adapted from previous studies (1, 2) with minor modifications. Briefly, mice were placed in a two-chamber arena (40 cm × 20 cm × 20 cm) where they could freely explore both chambers through a small opening. For each trial, mice were initially placed in the non-stimulation chamber (randomly assigned). Acute stimulation (DBS or tACS) was continuously delivered whenever the animal entered the stimulation chamber and ceased as soon as the mouse exited. Each trial lasted for 15 min, after which the mice were returned to their home cages. The chambers were cleaned with 70% alcohol between trials. The mice were tracked using a video camera interfaced with EthoVision software (EthoVision 15.0, Noldus), which also controlled the stimulation and extracted behavioral parameters.

Conditioned place preference/aversion test. To evaluate the effects of chemogenetic activation or inhibition on specific circuits or projections, a two-compartment conditioned place preference test was performed, following previous studies (3) with minor modifications. The apparatus consisted of two distinct conditioning chambers with unique wall drawings and flooring. The test comprised three phases: pre-test, acquisition, and post-test. On day 1 (pre-test), mice were allowed to freely explore both chambers for 15 min. For mice with chemogenetic manipulation alone, on day 2-4 (acquisition), they were confined to one chamber (unpaired side) for 30 min. 3 h later, they were placed in the opposite side chamber (paired side) for 30 min following the administration of CNO. For mice undergoing chemogenetic manipulation in combination with DBS or tACS, the procedure was similar. However, during the 30-min session in the paired side, stimulation (DBS or tACS with a 3-min on/off cycle) was applied following CNO administration. On day 5 (post-test), mice were given free access to both chambers for 15 min without any stimulation. The time spent in each chamber was recorded

and analyzed to determine whether the mice developed a conditioned place preference or aversion to the or stimulation- or no-stimulation-paired chamber. The movement of the mice was tracked and analyzed using a video-tracking system (EthoVision 15.0, Noldus).

PTSD model. Single prolonged stress (SPS) was conducted as previously described (4). Briefly, the mice were restrained for 2 h in 50 mL polypropylene conical tubes with a nose hole for ventilation. Subsequently, the mice were forced to swim for 20 min in a plastic tub (20 cm diameter, 30 cm height) filled with water (24 ± 1 °C, 20 cm depth). Then, they were dried and allowed to recuperate for 15 min before being exposed to isoflurane and anesthetized until the characteristics of rapid breathing and loss of responses to toe and tail pinch were evident. After that, the mice were left undisturbed in their homecages for 7 days.

LFP recording and analysis. Neural signals were recorded by using a multichannel data acquisition system (Zeus, Bio-Signal Technologies, Nanjing, China). Local field potentials were down-sampled to 1000 Hz prior to analysis and filtered with a bandpass filter between 0 and 150 Hz. Raw data were stored for later offline analysis. The time course of the LFP power spectra was generated using spectrogram analysis (NeuroExplorer, Nex Technologies) and the resultant three-dimensional time frequency spectra were smoothed using a Gaussian filter. To construct LFP power spectrograms, a multi-taper periodogram method was employed (5 tapers). Frequency-Power curve was generated using NeuroExplorer software Power Spectra for Continuous function.

Coherence between LFP channels across different electrodes was calculated using the weighted phase lag index (wPLI) (5). The wPLI is a measure of phase-synchronization between LFP signals,

which is less affected by volume-conduction, noise and sample size. wPLI was estimated using the imaginary component of the ($\boldsymbol{S_{xy}}$) (Equations 1 and 2).

$$S_{xy} = A_x A_y e^{i(\varphi_x - \varphi_y)} \qquad \text{(Equations 1)}$$

$$\mathrm{wPLI} = \frac{\sum|\mathrm{imag}(S_{xy})||\mathrm{sgn}(S_{xy})|}{\sum|\mathrm{imag}(S_{xy})|} \qquad \text{(Equations 2)}$$

where $\boldsymbol{A_x}$ and $\boldsymbol{A_y}$ are instantaneous amplitudes; and $\boldsymbol{\varphi_x}$ and $\boldsymbol{\varphi_y}$ are instantaneous phases for vCA1 and EC (LEC or MEC) signals, respectively.

Optogenetic manipulation. For optogenetic manipulation during behavioral assays, a 473-nm (blue light) or 589-nm laser (yellow light) (Hangzhou Newdoon Technology Co. Ltd) was connected to a patch cord with connectors on each end. For optogenetic inhibition of PV neurons, AAV-DIO-NpHR-mCherry or AAV-DIO-mCherry was injected into the vCA1 of PV-Cre mice, where optic fibers (200 μm in diameter, 0.37 NA) were implanted to allow inhibition at the cell bodies of PV neurons. To optogenetically inhibit LEC neuron terminals in the vCA1, vCA3, or vDG, AAV-DIO-NpHR-mCherry or control virus was injected in LEC and optical fibers were individually implanted into vCA1, vCA3, or vDG. The mice were tethered to optic fiber patch cords using ceramic mating sleeves and received photoinhibition (589 nm, 8–10 mW) in a continuous pattern during presentation of every 30-s CS (exceeding 5 s before and after the CS to ensure the light delivery covered the CS exposure).

For optogenetic stimulation of PV neurons and terminals from LEC and MEC with ChR2, mice were tethered to optic fiber patch cords and received photostimulation (473 nm, 4–6 mW) in 10 ms pulses at 40 Hz during presentation of every 30-s CS (exceeding 5 s before and after the CS to ensure the light delivery covered the CS exposure).

Chemogenetic manipulation. For chemogenetic activation experiments, AAV-DIO-hM3Dq-EGFP or AAV-DIO-EGFP was bilaterally injected into LEC of Sim1-Cre mice and cannulas were implanted into vCA1. After four weeks to allow viral expression, clozapine N-oxide (CNO, 1 mM) was applied into the cannulas with a microinjection pump (RWD Ltd.) at a rate of 0.2 μl/min and then an additional minute was given to allow the drug to be locally delivered into the LEC axon terminal fields.

For LEC-vCA1 projection inhibition, AAV2/1-Flp was injected into LEC and AAV-Cre (on)/Flp (on)-hM4Di-EGFP or AAV-Cre (on)/Flp (on)-EGFP into vCA1 of PV-Cre mice. For chemogenetic inhibition experiments, AAV-DIO-hM4Di-mCherry or AAV-DIO-mCherry was injected into vCA1 of PV-Cre, SST-Cre or VIP-Cre mice. After four weeks, mice were subjected to fear learning and extinction. CNO (1 mg/kg) was injected intraperitoneally 30 min before extinction training.

To inhibit vCA1-projecting LEC or MEC neurons, Retro-Cre-EGFP was injected into vCA1 and AAV-DIO-hM4Di-mCherry or control virus into LEC and MEC, respectively. After four weeks, mice were subjected to fear learning and extinction. CNO (1 mg/kg) was injected intraperitoneally 30 min before extinction training.

Fiber photometry. The fiber photometry system (ThinkerTech, Nanjing, China) allows for real-time recording of fluorescence signals from genetically encoded calcium indicators in freely moving mice. AAV-DIO-GCaMP6m was injected and optical fibers (200 μm in diameter, 0.37 NA) were individually implanted into vCA1 in PV-Cre, SST-Cre or VIP-Cre mice. To record the calcium signals

of vCA1-projecting LEC SIM1$^+$ layer 2a fan cells, AAV-Cre(on)/Flp(on)-GCaMP6s and Retro-Flp were individually injected into LEC and vCA1 in Sim1-Cre mice. Optical fibers were implanted into LEC. To record the effect of DBS manipulation (different frequencies) on calcium signals in PV neurons, AAV virus expressing GCaMP6m was injected and optical fibers were implanted in PV-Cre mice.

To record Ca^{2+} signals in fear-tagged neurons induced by optogenetic stimulation of vCA1 PV-INs, FosTRAP2::PV-Flp mice were injected with AAV-DIO-GCaMP6m and AAV-DIO-ChrimsonR at vCA1 for expression in fear-tagged neurons and PV neurons, respectively. Recordings in fear-tagged neurons were performed concurrently with PV neurons being stimulated through the same optic fiber. Optogenetic stimulation was delivered (λ = 638 nm) at 40 Hz and fiber photometry recordings were made using an Arduino board running custom code synchronized to the photometry system.

To simultaneously record calcium signals of PV neurons and fear-tagged neurons in vCA1 at a 100-Hz sampling rate, different excitation wavelengths were used (470 nm for Ca^{2+}-dependent GCaMP6m activity, 580 nm for Ca^{2+}-dependent jRGECO1a activity). To minimize photo-bleaching, the laser power at the tip of the optical fiber was adjusted to a low level of 40-60 μW.

The emitted fluorescence signals were collected and converted into electrical signals to reflect neural activity. The analog voltage signals were digitalized at 100 Hz using a Power 1401 digitizer and Spike2 software (CED, Cambridge, UK). Photometry data were further analyzed using MATLAB. The data were segmented and aligned to the onset of trigger events within individual trials. Fluorescence changes ($\Delta F/F$) were calculated as $(F - F_0)/F_0$, where F_0 is the mean fluorescence signal for 10 s before

the trigger event. $\Delta F/F$ values are presented as heatmaps, single trial curve or average plots with the shaded area indicating the SEM.

Deep brain stimulation (DBS). The implanted bipolar DBS electrode made of platinum-iridium wire (76.2 μm diameter, coated, AM Systems) was connected to the stimulator by means of a flexible cable, allowing free movement of the animal. Mice were housed individually after the surgery during the entire period of the experiments. Electrical stimulation was delivered through a stimulator (STG4008; Multi Channel Systems, Reutlingen, German), which generates square-wave biphasic current pulses. Mice received stimulation at 20 Hz (50 μA and pulse width of 5 ms), 40 Hz (50 μA and pulse width of 5 ms) or 130 Hz (50 μA and pulse width of 70 μs). In sham-DBS control experiments, mice were connected to the external cable but not subjected to electrical stimulation. Current and frequencies were chosen based on previous studies in the literature (6).

Transcranial alternating current stimulation (tACS). Upon exposure of the skull, a burr hole craniotomy was drilled using drill bit in the skull at a location chosen to target LEC (AP, –4.16 mm; ML, ±4.20 mm; DV, –4.85 mm). The dura was left intact. Bone anchor screws were driven approximately half-way through the skull and served as anode electrodes for tACS. Another electrode was inserted into the neck muscles and served as cathode electrodes. The stimulation group received tACS with 40 Hz of 200 μA over the bilateral LEC (STG4008; Multi Channel Systems, Reutlingen, German).

Computational model and solution method. The Finite Element Method (FEM) was used to simulate the electric fields engendered by transcranial electric stimulation. The FEM model was

meticulously constructed based upon the Digimouse dataset (7), a comprehensive whole-body computed tomography compendium of a mouse, boasting a high resolution (~0.1 mm), curated by Alekseichuk et al. (8).

In the initial phase of the current study, segmentation of brain tissues was executed, and integration of the electrode was accomplished utilizing the ITK-SNAP software (9). The placement of all electrodes was meticulously aligned with the protocols used in the animal experiments. Subsequently, a tetrahedral-based FEM model was made to incorporate in excess of 5 million tetrahedral elements, through a series of modified segmentation procedures and the application of iso2mesh (10). Then, the FEM model was processed using Gmsh (11) and subsequently exported as a Nastran Bulk Data File for further computational analysis.

To define conductivities and compute the physics equations, COMSOL Multiphysics 4.3 (COMSOL, Inc., Burlington, MA) was employed. The conductivities were defined as follows (in Siemens per meter, S/m): white matter and grey matter, 0.126; Cerebrospinal Fluid (CSF), 1.654; Bone, 0.01; Scalp, 0.465; Eye Balls, 0.5; Copper, 7×10^6; Platinum-iridium alloy, 6×10^7.

The nature of the study was delineated as steady-state current studies, and the initial state of the electric field was predetermined to be zero. The electric field was derived through the injection of current (200 μA) via a stimulator (STG4008; Multi Channel Systems, Reutlingen, German). The magnitude of the electric field (E) was calculated as follows (Equations 3):

$$E = \sqrt{Ex^2 + Ey^2 + Ez^2} \qquad \text{(Equations 3)}$$

where x, y, and z represent the respective orthogonal directions.

Histology and fluorescent immunostaining. Animals were deeply anesthetized with 1% sodium pentobarbital and were transcardially perfused with 1 × PBS, followed by 4% paraformaldehyde (PFA) in 1 × PBS. Brains were extracted and post-fixed overnight at 4 °C in 4% PFA (pH = 7.2) and 40-μm coronal sections were collected using a vibratome (VT1000S, Leica). After a 15-min incubation in a 4,6-Diamidino-2-phenylindole dihydrochloride hydrate (DAPI) solution (1:2000), sections were washed three times (15 min each time) in PBS with 0.1% Tween-20. Slides were mounted in the dark with glass coverslips using mounting media. The coverslips were sealed to the slide with nail polish. All fluorescent images were collected by taking serial z-stack images through 10 × or 20 × objectives of a confocal microscope (Digital Eclipse A1R+, Nikon).

For c-Fos staining, brain slices were washed three times (5 min each time) with 1 × PBS and then blocked with 10% normal donkey serum in 1 × PBS with 0.3% Triton X-100 (PBST) for 1 h, after which they were incubated overnight at 4 °C with rabbit anti-c-Fos (1:500, Cell Signaling Technology, catalog no. 2250S). Sections were then washed with PBS, incubated in 2% normal donkey serum for 10 min, and then incubated for 2 h with Alexa Fluor 488 donkey anti-rabbit IgG or Alexa Fluor 568 donkey anti-rabbit (ThermoFisher Scientific).

For labeling PV, SST, VIP or Reelin, mice were perfused under deep anesthesia with PBS followed by 4% PFA. The brains were post-fixed in PFA for 2 h and stored in a 30% sucrose solution for 1-2 days at 4 °C. Brain sections (40-μm) were prepared with a cryostat (CM1900, Leica). Slices were incubated in the blocking solution with mouse anti-PV (1:300, Sigma-Aldrich, catalog no. MAB1572), goat anti-somatostatin (SST) (1:500, Santa Cruz, catalog no. sc-7819), rabbit anti-VIP (1:500,

Immunostar, catalog no. 20077) or rabbit anti-Reelin (1:500, Invitrogen, catalog no. PA5-78413) at 4 °C overnight and then with the secondary antibody (Alexa Fluor 568 donkey anti-mouse IgG, Alexa Fluor 488 donkey anti-mouse IgG, Alexa Fluor 647 donkey anti-mouse IgG, Alexa Fluor 568 donkey anti-goat IgG, Alexa Fluor 568 donkey anti-rabbit IgG or Alexa Fluor 647 donkey anti-rabbit IgG (all 1:500, Thermo Fisher Scientific) for 2 h. Slices were washed in 1 × PBS with 0.1% Tween-20, mounted onto slides, and covered with coverslips with ProLong Gold Antifade Mountant (Invitrogen).

Slice electrophysiology. Whole-cell recordings were performed in acute brain slices. Mice were deeply anesthetized with 1% sodium pentobarbital and subsequently decapitated. Brains were dissected quickly and chilled in well-oxygenated (95% O_2/5% CO_2, v/v) ice-cold artificial cerebrospinal fluid (ACSF) containing the following (in mM): 125 NaCl, 2.5 KCl, 12.5 D-glucose, 1 $MgCl_2$, 2 $CaCl_2$, 1.25 NaH_2PO_4, and 25 $NaHCO_3$ (pH 7.35-7.45). Coronal brain slices (300-μm thick) containing regions of interest were cut with a vibratome (Leica VT1000S, Germany). After recovery for 1 h in oxygenated ACSF at 30 ± 1 °C, each slice was transferred to a recording chamber and was continuously superfused with oxygenated ACSF at a rate of 1–2 ml per minute. The neurons in vCA1 were patched under visual guidance using infrared differential-interference contrast microscopy (BX51WI, Olympus) and an optiMOS camera (QImaging). The slices were continuously perfused with well-oxygenated ACSF at 35 ± 1 °C during all electrophysiological studies. Whole-cell patch clamp recordings were performed using an Axon 200B amplifier (Molecular Devices). Membranous currents were sampled and analyzed using a Digidata 1440 interface and a personal computer running Clampex and Clampfit software (Version 10.5, Axon Instruments). Optical stimulation of ChR2-, or NpHR-expressing neurons was performed using a collimated LED (Lumen Dynamics) with peak wavelengths of 473, 589 nm, respectively. The LED was connected to an Axon 200B amplifier to trigger

photostimulation. The brain slice in the recording chamber was illuminated through a 40 × water-immersion objective lens (LUMPLFLN 40XW, Olympus). To verify the functional potency of NpHR-mediated optogenetic inhibition, yellow light (λ = 589 nm, 1-s pulse) was delivered to generate outward photocurrents under voltage-clamp mode, which promoted membrane hyperpolarization to reduce spikes produced by current injection under current-clamp mode. The functional potency of the ChR2-expressing virus was validated by measuring the number of action potentials evoked by using 40 Hz of blue-light stimulation (1 ms, λ = 473 nm). To evoke synaptic responses of PV neurons in the vCA1 by optogenetic photostimulation of LEC axons, the slice was illuminated with blue-light pulses of 5-ms durations. For light-evoked EPSCs, the recording pipettes (3–5 MΩ) were filled with a solution containing the following (in mM): 132.5 cesium gluconate, 17.5 CsCl, 2 $MgCl_2$, 0.5 EGTA, 10 HEPES, 4 Mg-ATP, and 5 QX-314 chloride (280–300 mOsm, pH 7.2 with CsOH). TTX (1 μM TTX), 4-AP (100 μM), and NBQX (10 μM) were diluted in ACSF and applied through superfusion. For the feedforward inhibition test, light-evoked EPSCs and IPSCs were recorded at −70 mV and 0 mV, respectively, with 5-ms blue laser pulses every 20 s. For pharmacological isolation of PV-IPSCs specifically, PV-IPSCs were blocked using 0.5 μM of ω-agatoxin IVA, a selective antagonist for P/Q-type calcium channels (ApexBio, B7192). Data were analyzed by the Mini Analysis Program (Synaptosoft) with an amplitude threshold of 20 mV.

Engram labeling. Recombination was induced with 4-hydroxytamoxifen (4-OHT, Sigma-Aldrich, Catalog no. H6278). In brief, 4-OHT was dissolved at 20 mg/ml in ethanol by shaking at 37 °C for 30 min and then stored in aliquots at –20 °C for up to several weeks. Before use, 4-OHT was re-dissolved in ethanol by shaking at 37 °C. Corn oil (Sigma-Aldrich, Catalog no. C8267) was then added for a final concentration of 10 mg/ml 4-OHT, and the ethanol was evaporated by vacuum under

centrifugation. The final 10 mg/ml 4-OHT solutions were stored at 4 °C for < 24 h before use. All injections were delivered intraperitoneally (i.p.). Mice were transported from the vivarium to an adjacent holding room at least 3 h before the 4-OHT injection to minimize transportation-induced immediate early gene activity. Activity-dependent neuronal labeling was induced by a single intraperitoneal injection of 4-OHT (50 mg/kg mice) administered before the conditioning session for the fear-labeled mice. Mice were then returned to the vivarium with a regular 12 h light-dark cycle for the remainder of the experiment.

In vivo electrophysiological recording. For in vivo optogenetic tagging of PV-INs, PV-Cre mice were unilaterally injected with AAV DIO ChR2-mCherry at vCA1 (AP, –3.20 mm; ML, ±3.08 mm; DV, –4.0 mm). Three weeks later, custom-made optrodes (consisting of an optical fiber and 8 tetrodes) were implanted at the same coordinates where the virus was injected. The tip of the optic fiber was 300 μm above the tip of the electrodes. Each tetrode was made of four twisted fine platinum/iridium wires (12.5 μm diameter, California Fine Wire). Silver wires with two screws were attached to the skull as ground. After a 7-day recovery from the surgery, the mice were habituated to the headstage and cables connected to the electrode on their heads for several days prior to electrophysiological recordings. Spiking activities were digitized at 30 kHz, band-pass filtered between 250 and 8000 Hz, and stored on a PC for further offline analysis.

Spike sorting and unit classification with optogenetic tagging. Briefly, data were exported to Offline Sorter (Plexon Inc.) and NeuroExplorer (Nex Technologies) for offline analysis. Spike waveforms were identified by threshold crossing and sorted into units (presumptive neurons) by principal component analysis (PCA). Waveforms with inter-spike intervals (ISIs) less than the

refractory period (2 ms) were excluded. Only units showing spikes with a signal-to-noise ratio larger than 2 were considered as neurons and included. Cross-correlation histograms were plotted to ensure that no unit was discriminated against more than once on different channels. All units were further classified as wide-spiking (WS) putative pyramidal neurons, narrow-spiking (NS) INs and fasts-piking parvalbumin (FS-PV) INs based on the peak-trough latency and the baseline firing rate. A unit with > 400 μs of peak-trough latency was classified as a WS neuron, with ≤400 μs of peak-trough latency was classified as an NS-IN and NS-IN with firing rate > 10 Hz was classified as an FS-PV IN.

For optical identification of vCA1 PV neurons, blue-light pulses (470 nm, 5-ms pulse duration, 1 Hz) were delivered at the end of each recording session. To assess whether these units were driven directly by ChR2 or indirectly by synaptic connections, we analyzed the onset latency relative to each light pulse. Only spikes with short spike latency (< 5 ms) and low jitter (< 3 ms) after light-pulse illumination were considered as being directly stimulated in this study. Only when the waveforms of laser-evoked and spontaneously generated spikes were highly similar (correlation coefficient >0.9), then they were considered to originate from the same unit (12).

Single unit firing analysis. To analyze vCA1 neuronal activity during 30-s trials, z-scored peristimulus time histograms (PSTHs) were calculated for each individual neuron, averaged over 8 CS trials for extinction retrieval, with or without DBS-vCA1 in extinction learning. Spikes were divided into 500-ms bins for visualizing individual unit responses and comparing responses between groups. A z-score was calculated for each bin relative to the 10-s prestimulus activity by subtracting average firing rate during baseline and by dividing the difference by the baseline standard deviation. For a given unit, firing rates during extinction retrieval and during baseline were compared to determine the

significance of firing rate difference between these two conditions (paired Student's *t* test). To compare PSTHs between groups, the mean z-score was calculated for each unit by averaging z-scored PSTHs during extinction retrieval (30-s CS). Response heatmaps were generated from z-scored PSTHs of units sorted by their mean z-score during 30-s CS in extinction retrieval.

Supplemental Figures and Legends

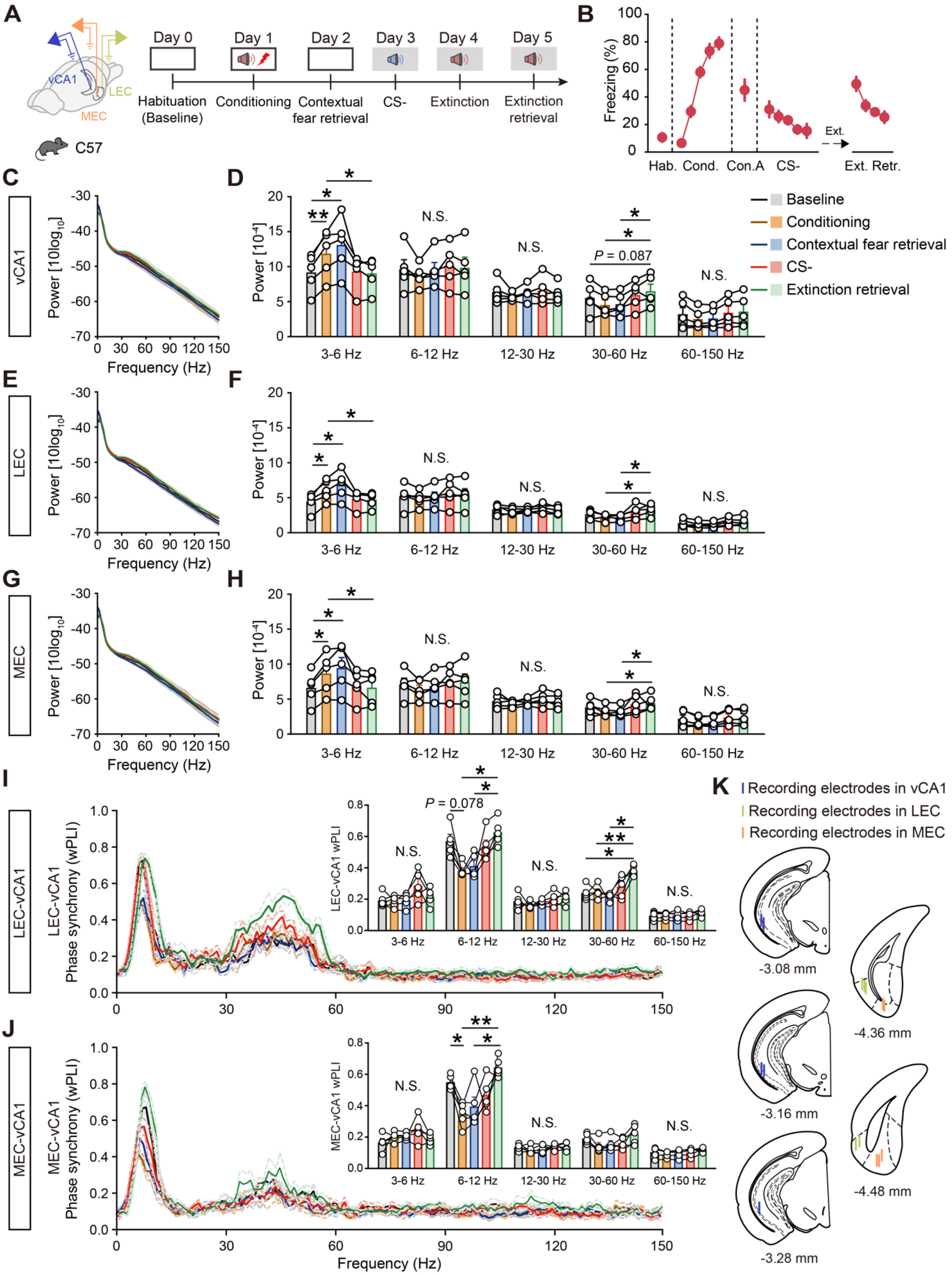

Supplemental Figure 1. In vivo electrophysiological recordings during baseline, fear

conditioning, contextual fear retrieval, CS-, and extinction retrieval. (**A**) Schematics of electrode implantation and experimental design. (**B**) Time courses of freezing responses during habituation, fear conditioning, contextual fear retrieval, CS-, and extinction retrieval. (**C**, **E** and **G**) Power spectrum of vCA1 (**C**), LEC (**E**) and MEC (**G**) LFP recordings during baseline, fear conditioning, contextual fear retrieval, CS-, and extinction retrieval. Solid lines represent the averages and shaded areas indicate SEM. (**D, F** and **H**) Average power of vCA1 (**D**), LEC (**F**) and MEC (**H**) LFP recordings during baseline, fear conditioning, contextual fear retrieval, CS-, and extinction retrieval. Histograms represent mean ± SEM and circles denote individual mice. $n = 5$. N.S., no significant difference, *$P < 0.05$, **$P < 0.01$. (**I** and **J**) Phase synchrony for LEC-vCA1 (**I**) and MEC-vCA1 (**J**) LFPs weighted phase lag index (wPLI) during baseline, fear conditioning, contextual fear retrieval, CS-, and extinction retrieval, respectively. The inset shows different phase synchrony quantified using the wPLI. Histograms represent mean ± SEM and circles denote individual mice. n = 5. N.S., no significant difference, *$P < 0.05$, **$P < 0.01$. (**K**) Location of center for LFP electrode lesions for all mice. Repeated measures one-way ANOVA with Tukey's multiple comparisons test in (**D**, **F**, **H**, **I** and **J**).

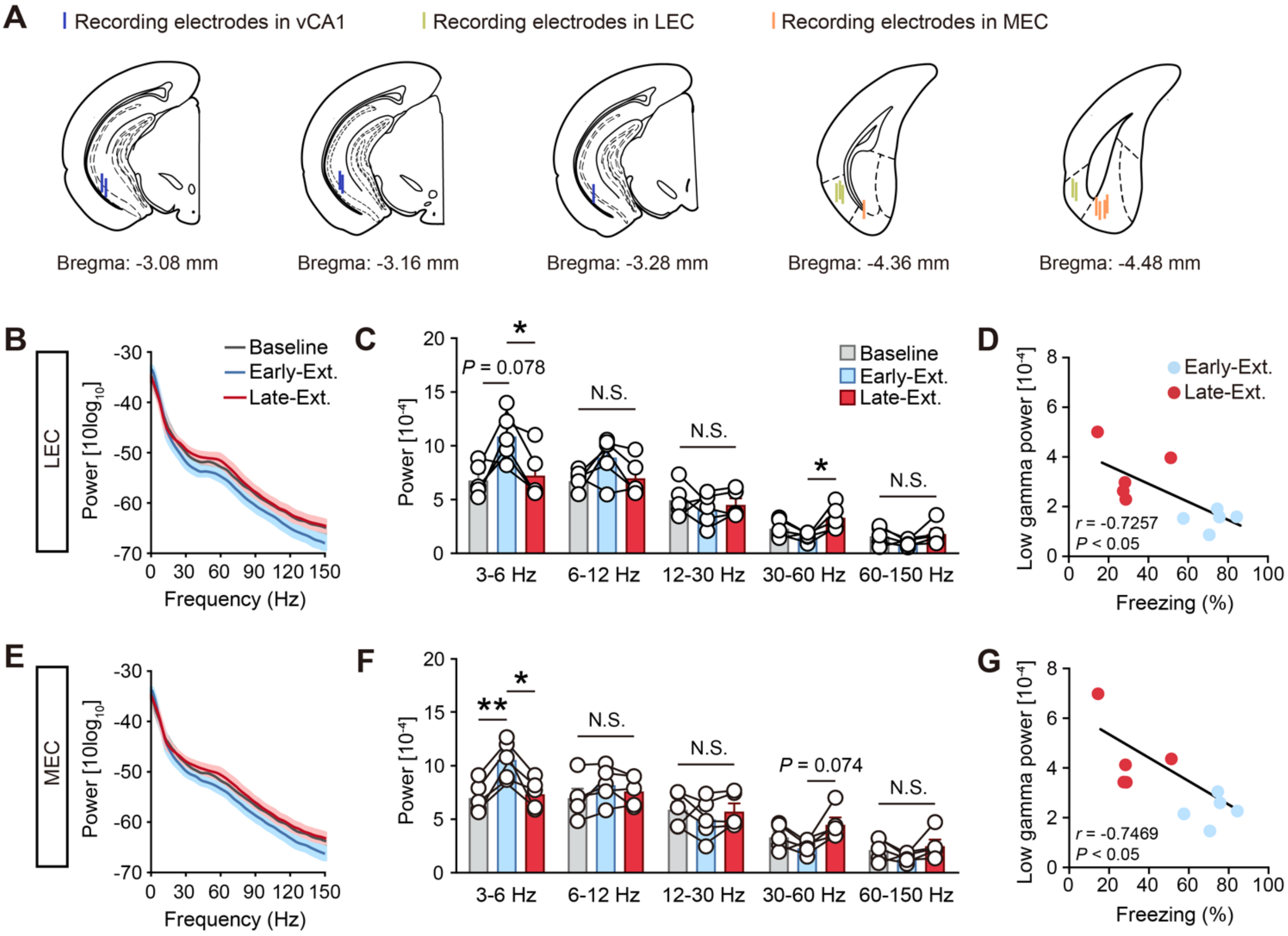

Supplemental Figure 2. Effects of fear extinction on different frequency oscillations in the LEC and MEC. (**A**) Location of center for LFP electrode lesions for all mice. (**B** and **E**) Power spectrum of LEC (**B**) and MEC (**E**) LFP recordings during Baseline, Early-Ext. and Late Ext.. Solid lines represent the averages and shaded areas indicate SEM. (**C** and **F**) Average power of LEC (**C**) and MEC (**F**) LFP recordings during Baseline, Early-Ext. and Late Ext.. Histograms represent mean ± SEM and circles denote individual mice. $n = 5$. N.S., no significant difference, *$P < 0.05$, **$P < 0.01$. (**D** and **G**) Linear regression of freezing responses vs LEC (**D**) and MEC (**G**) low-gamma power during Early-Ext. and Late Ext. sessions. Repeated measures one-way ANOVA with Tukey's multiple comparisons test in (**C** and **F**).

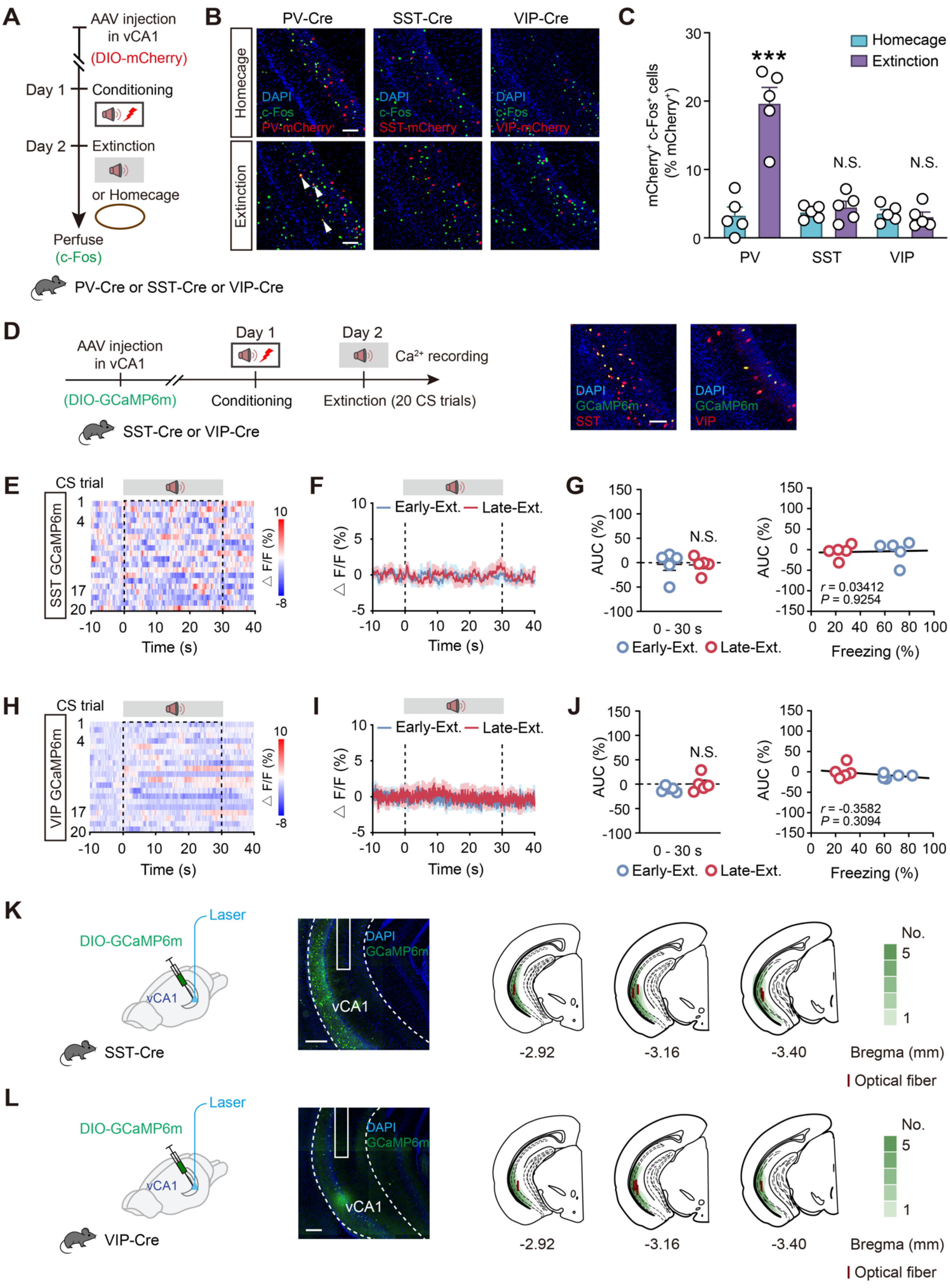

Supplemental Figure 3. vCA1 PV-INs but not SST-INs or VIP-INs were activated in the

extinction. (**A**) Schematics of AAV injections and experimental design. (**B**) Representative images of mCherry$^+$ (red) and c-Fos$^+$ (green) immunofluorescence in the vCA1. The white arrowheads denote colabeled mCherry$^+$/c-Fos$^+$ cells. Scale bar, 100 μm. (**C**) Histograms represent mean ± SEM and circles denote individual mice. The number of reactivated (mCherry$^+$/c-Fos$^+$) PV-INs was significantly higher in the extinction group than the homecage group. Extinction group, n = 5 mice; homecage group, n = 5 mice. N.S., no significant difference, ***$P < 0.001$. (**D**) Schematics of AAV injections, experimental design and immunostaining confirming the specificity of GCaMP6m expression in the SST-INs and VIP-INs. Scale bar, 100 μm. (**E**) Heatmap of calcium signals in the SST-INs during extinction training sessions. (**F**) Average calcium signals in the SST-INs during Early-Ext. and Late-Ext. Data are mean ± SEM. n = 5 mice. (**G**) Activity of the SST-INs and correlation of freezing responses with the calcium signals during Early-Ext. and Late-Ext. Quantification of AUC of calcium signals in the SST-INs (left). Data are mean ± SEM. N.S., no significant difference. Linear regression of freezing responses vs AUC of SST-INs GCaMP signals during Early-Ext. and Late Ext. (right). (**H**–**J**) The same as (**E**–**G**) for the VIP-INs. Data are mean ± SEM. n = 5 mice. N.S., no significant difference. (**K** and **L**) Schematics of AAV injections and representative images of virus expression (left). Scale bar, 200 μm. Location of center for optical fiber lesions and virus expression for all mice (right). Unpaired Student's t test in (**C**) and paired Student's t test in (**G** and **J**).

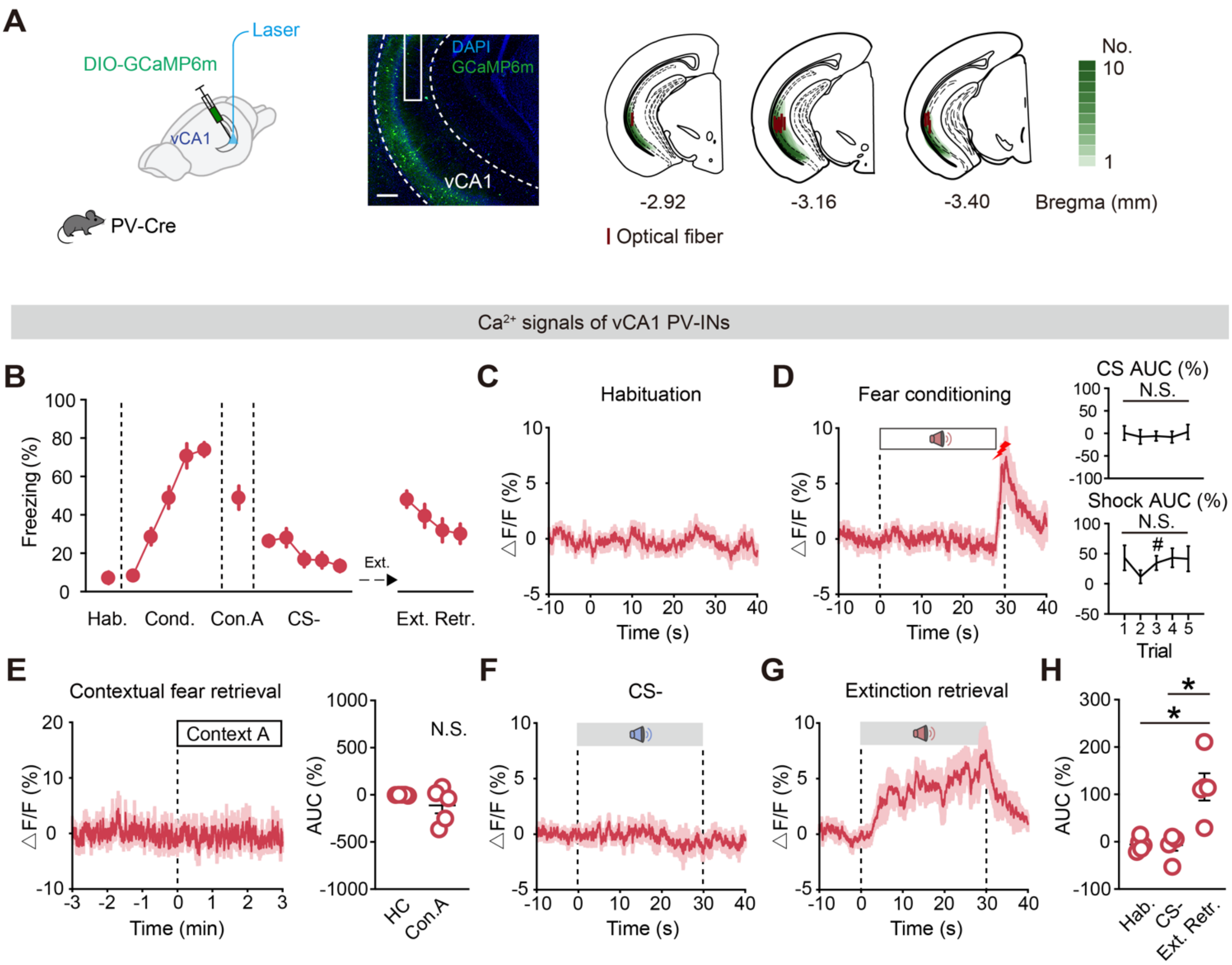

Supplemental Figure 4. Ca^{2+} recordings of vCA1 PV-INs during habituation, fear conditioning, contextual fear retrieval, CS- and extinction retrieval. (**A**) Schematics of stereotaxic surgery and representative image of virus expression (left). Scale bar, 200 μm. Location of center for optical fiber lesions and virus expression for all mice (right). (**B**) Time courses of freezing responses during habituation, fear conditioning, contextual fear retrieval, CS-, and extinction retrieval. (**C**) Average calcium signals in the PV-INs during habituation. (**D**) Average Ca^{2+} signals in the PV-INs during fear conditioning (left) and quantification of AUC of calcium signals (right). Repeated measures one-way ANOVA: CS AUC, $F_{1.567,\ 6.267} = 0.1307$, $P = 0.8331$; Shock AUC, $F_{2.122,\ 8.489} = 0.9933$, $P = 0.4145$. N.S., no significant difference. One-sample *t* test with hypothetical mean of zero, $^{\#}P < 0.05$. (**E**) Average calcium signals in the PV-INs during contextual fear retrieval (left) and quantification of AUC of calcium signals (right). Data are mean ± SEM. n = 5 mice. N.S., no significant difference. (**F** and **G**) Average calcium signals in the PV-INs during CS- (**F**) and extinction retrieval (**G**). (**H**) Quantification of AUC of Ca^{2+} signals. Data are mean ± SEM. n = 5 mice. $^{*}P < 0.05$. Repeated measures one-way ANOVA and one-sample *t* test with hypothetical mean of zero in (**D**), paired Student's *t* test in (**E**) and repeated measures one-way ANOVA with Tukey's multiple comparisons test in (**H**).

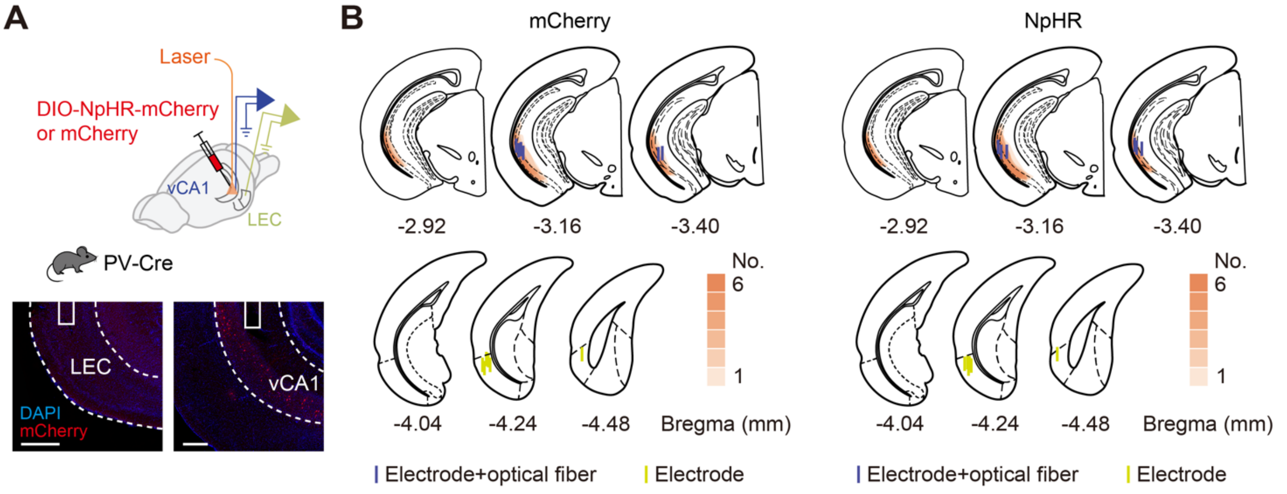

Supplemental Figure 5. Histological validation of the virus, LFP electrode and optical fiber location. (**A**) Schematics of stereotaxic surgery and representative images of virus expression. Scale bar, 200 μm. (**B**) Location of center for LFP electrode and optical fiber lesions and virus expression for all mice.

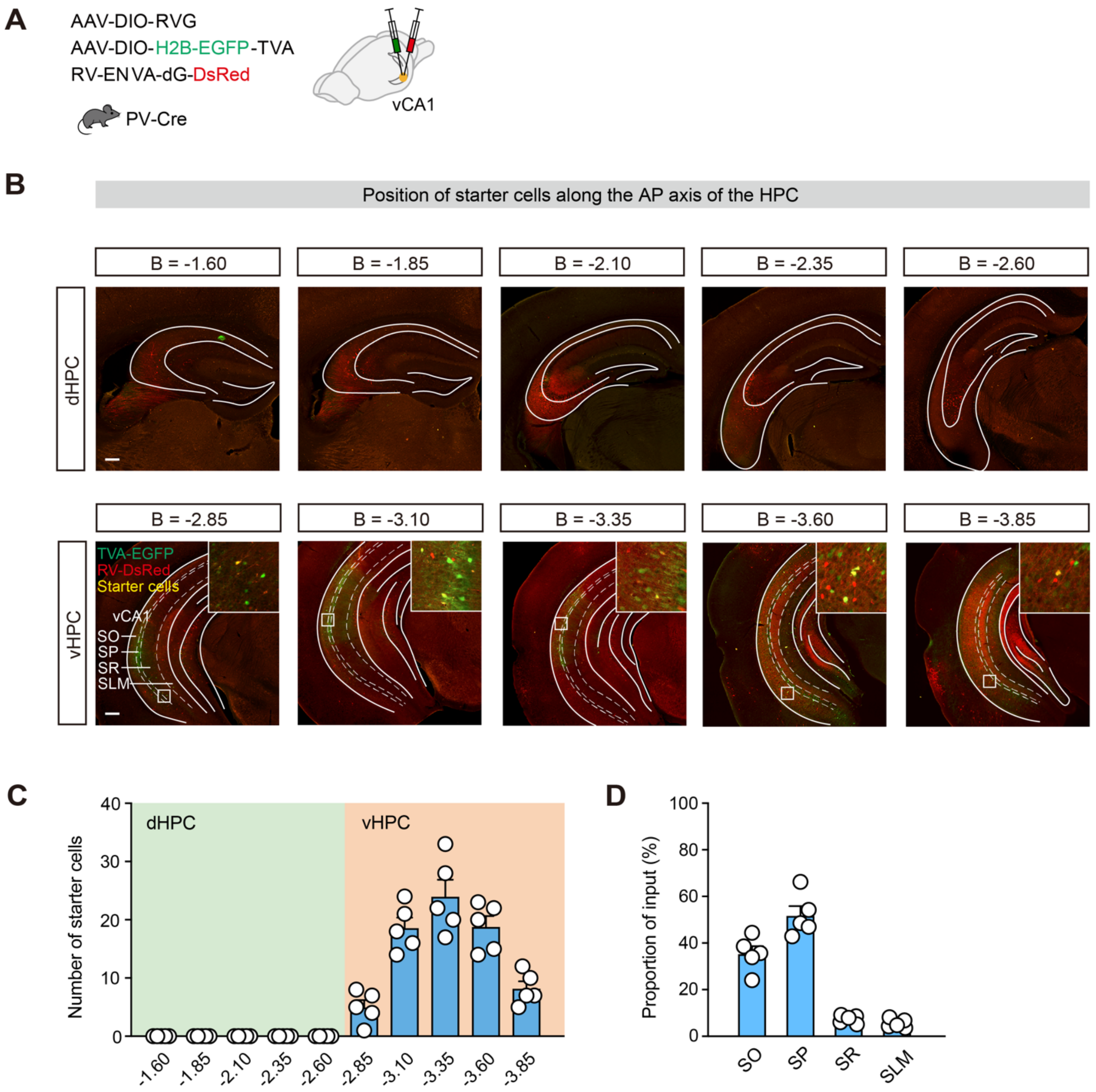

Supplemental Figure 6. Analysis of spatial distribution of starter cells. (**A**) Schematics of AAV injections and experimental design. (**B**) Starter cells at different bregma sites in dHPC and vHPC of PV-Cre mice. Scale bar, 200 μm. (**C**) Data showing the number of starter cells at different bregma sites in dHPC and vHPC of PV-Cre mice. (**D**) Proportion of starter cells in different vCA1 layers. dHPC, dorsal hippocampus; vHPC, ventral hippocampus; SO, stratum oriens; SP, stratum pyramidal; SR, stratum radiatum; SLM, stratum lacunosum moleculare. Data are mean ± SEM. *n* = 5 mice.

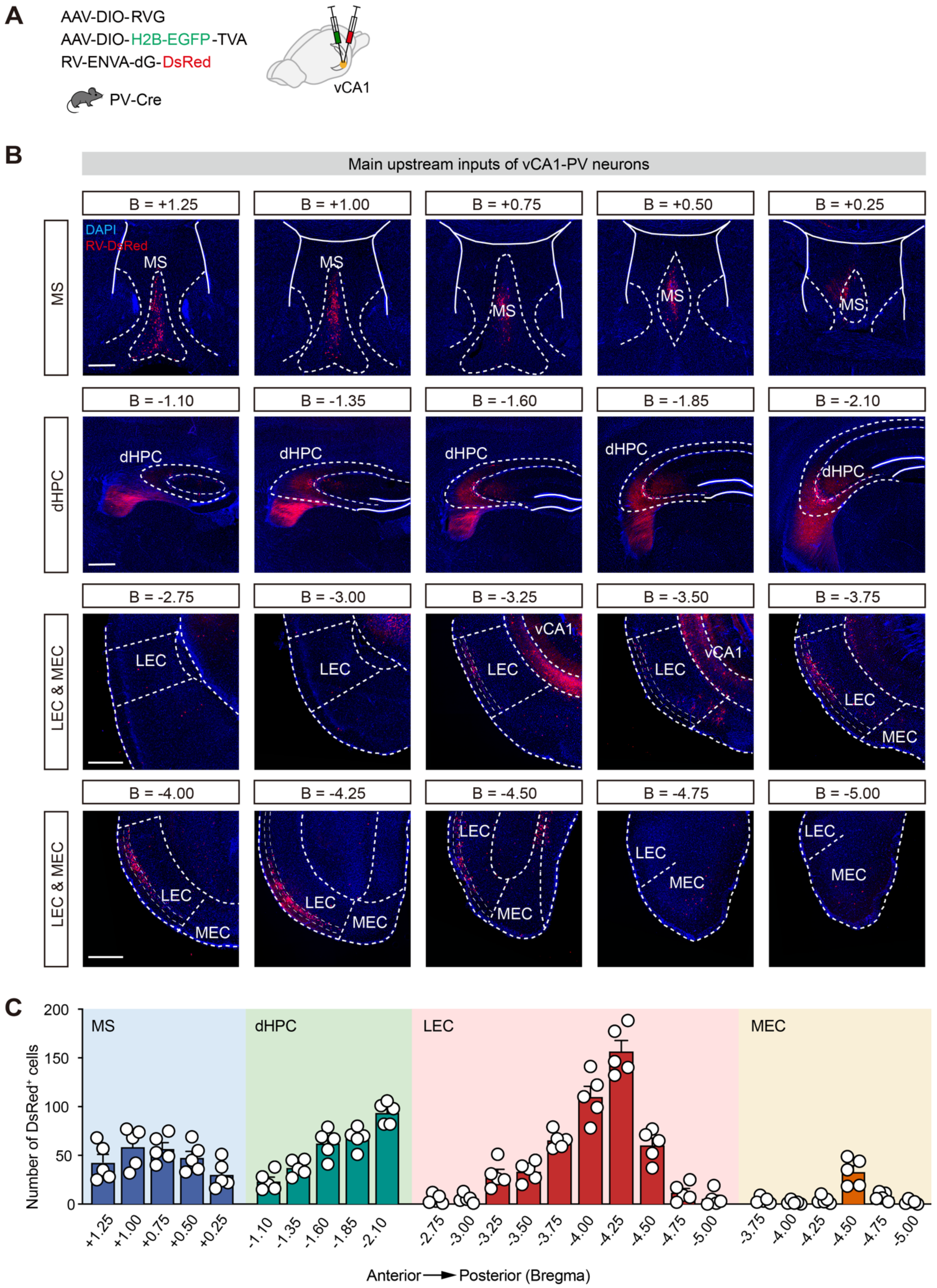

Supplemental Figure 7. vCA1 PV projecting neurons at different bregma sites in MS, dHPC and EC. (**A**) Schematics of AAV injections and experimental design. (**B**) DsRed-labeled neurons at

different bregma sites in MS, dHPC and EC of PV-Cre mice. Scale bar, 500 μm. (**C**) Data showing the number of DsRed-labeled cells at different bregma sites in MS, dHPC and EC of PV-Cre mice. Data are mean ± SEM. *n* =5 mice. MS, medial septal nucleus; dHPC, dorsal hippocampus; LEC, lateral entorhinal cortex; MEC, medial entorhinal cortex.

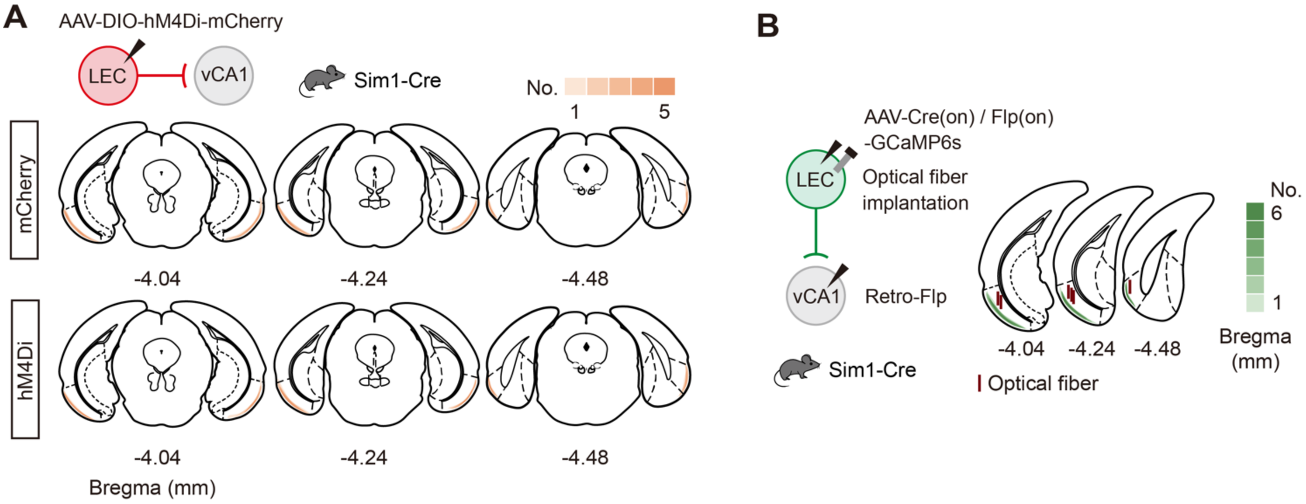

Supplemental Figure 8. Histological validation of the virus and optical fiber location. (**A**) Schematics of stereotaxic surgery and location of virus expression for all mice. (**B**) Schematics of stereotaxic surgery and location of center for optical fiber lesions and virus expression for all mice.

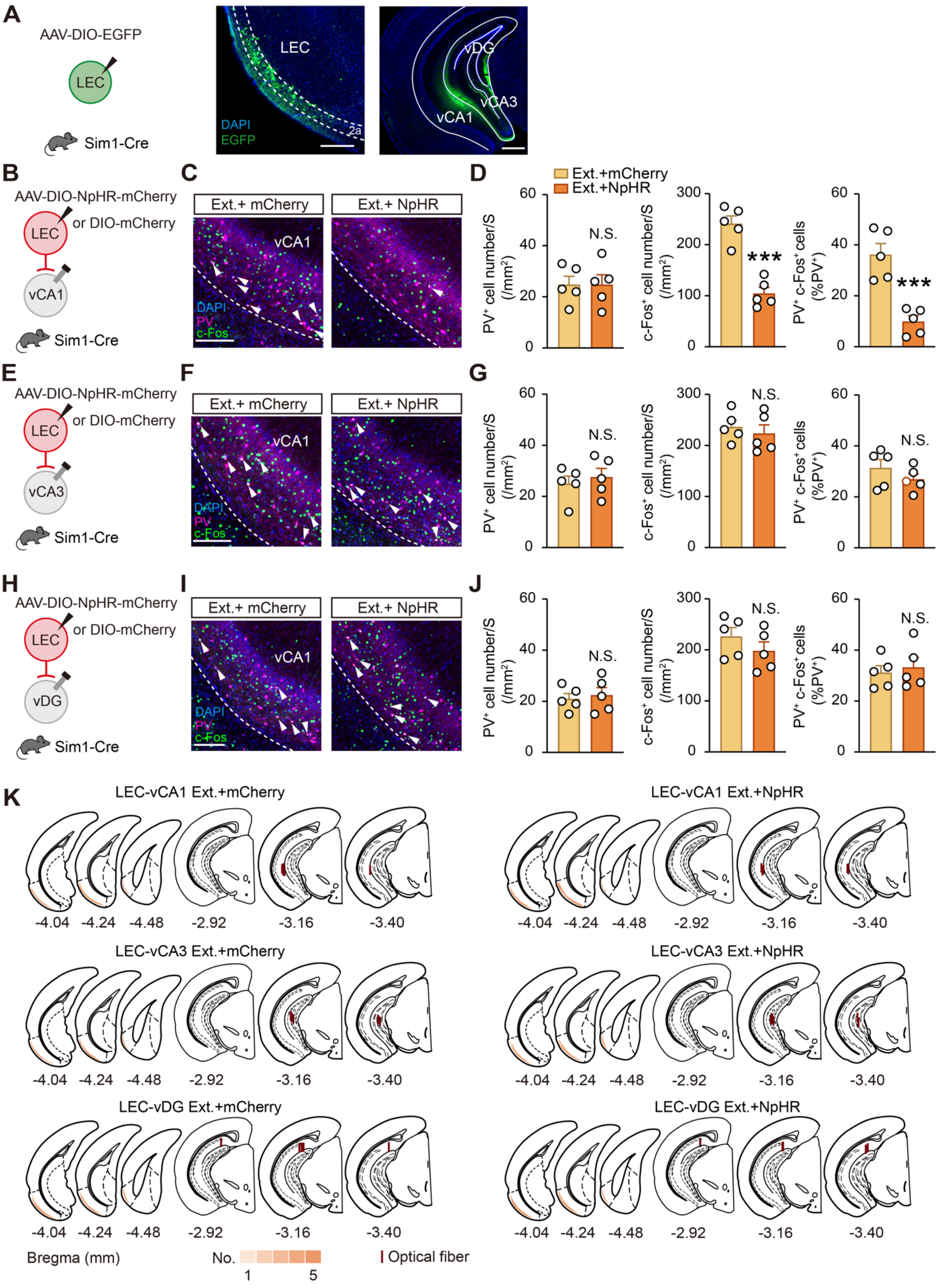

Supplemental Figure 9. Effects of manipulating LEC layer 2a fan cells-vCA1/vCA3/vDG

pathway on vCA1 PV-INs during fear extinction. (**A**) Schematics of AAV injections, representative images of virus expression and the axon projection targets of LEC layer 2a fan cells. Scale bar, 200 μm (left). Scale bar, 500 μm (right). (**B**) Schematics of AAV injections and experimental design. Optogenetic stimulation (yellow light) was delivered during extinction training. (**C**) Representative images of PV (purple) and c-Fos$^+$ (green) immunofluorescence in vCA1. The white arrowheads denote colabeled PV$^+$/c-Fos$^+$ cells. Scale bar, 100 μm. (**D**) Summary graphs of PV-INs and c-Fos fluorescence in vCA1. Histograms represent mean ± SEM and circles denote individual mice. mCherry group, n = 5 mice; NpHR group, n = 5 mice. N.S., no significant difference, ***$P < 0.001$. (**E**–**G**) The same as (**B**–**D**) for inhibition of LEC-layer 2a→vCA3 projection. n = 5 mice per group. Histograms represent mean ± SEM and circles denote individual mice. N.S., no significant difference. (**H**–**J**) The same as (**B**–**D**) for inhibition of LEC-layer 2a→vDG projection. n = 5 mice per group. Histograms represent mean ± SEM and circles denote individual mice. N.S., no significant difference. (**K**) Location of virus expression and optical fiber lesions for all mice. Unpaired Student's t test in (**D**, **G** and **J**).

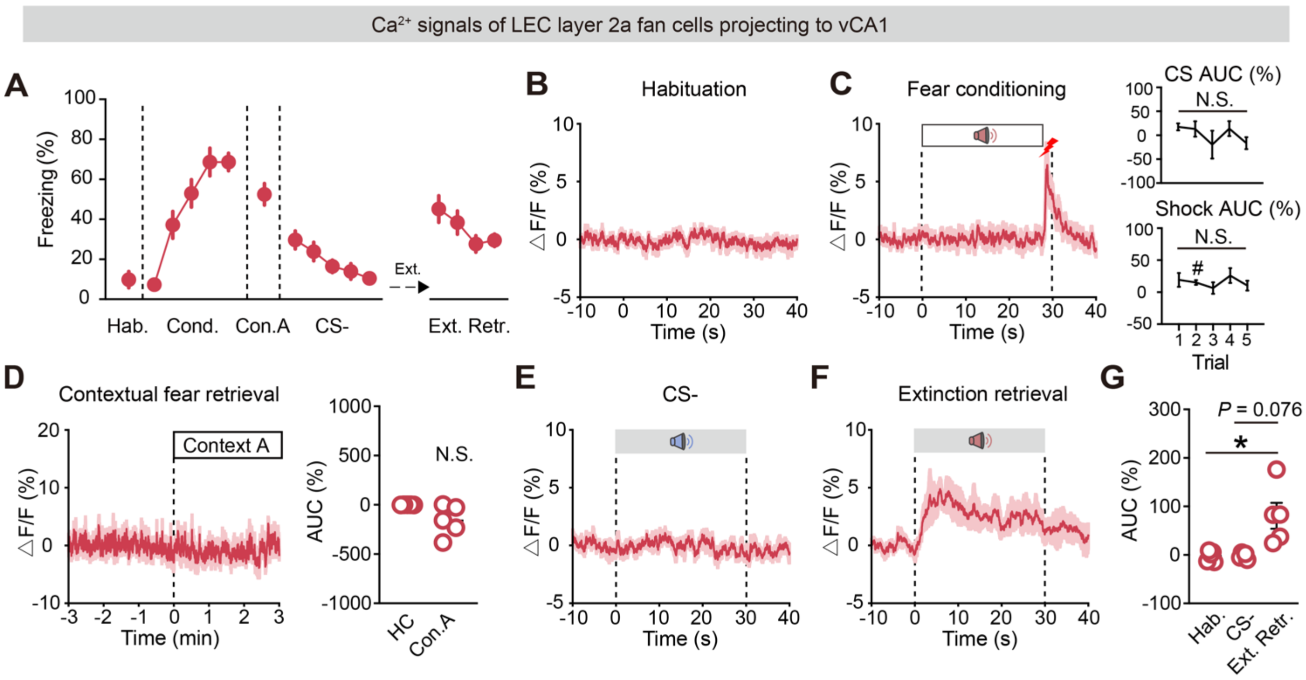

Supplemental Figure 10. Ca^{2+} recordings of the LEC-vCA1 pathway during habituation, fear conditioning, contextual fear retrieval, CS-, and extinction retrieval. (**A**) Time courses of freezing responses during habituation, fear conditioning, contextual fear retrieval, CS-, and extinction retrieval. (**B**) Average Ca^{2+} signals during habituation. (**C**) Average Ca^{2+} signals during fear conditioning (left) and quantification of AUC of Ca^{2+} signals (right). Repeated measures one-way ANOVA: CS AUC, $F_{1.786,\ 7.143}$ = 1.133, P = 0.3661; Shock AUC, $F_{2.044,\ 8.174}$ = 1.037, P = 0.3986. N.S., no significant difference. One-sample t test with hypothetical mean of zero, $^{\#}P < 0.05$. (**D**) Average Ca^{2+} signals during contextual fear retrieval (left) and quantification of AUC of Ca^{2+} signals (right). Data are mean ± SEM. n = 5 mice. N.S., no significant difference. (**E** and **F**) Average Ca^{2+} signals during CS- (**E**) and extinction retrieval (**F**). (**G**) Quantification of AUC of Ca^{2+} signals. Data are mean ± SEM. n = 5 mice. $^{*}P < 0.05$. Repeated measures one-way ANOVA and one-sample t test with hypothetical mean of zero in (**C**), paired Student's t test in (**D**) and repeated measures one-way ANOVA with Tukey's multiple comparisons test in (**G**).

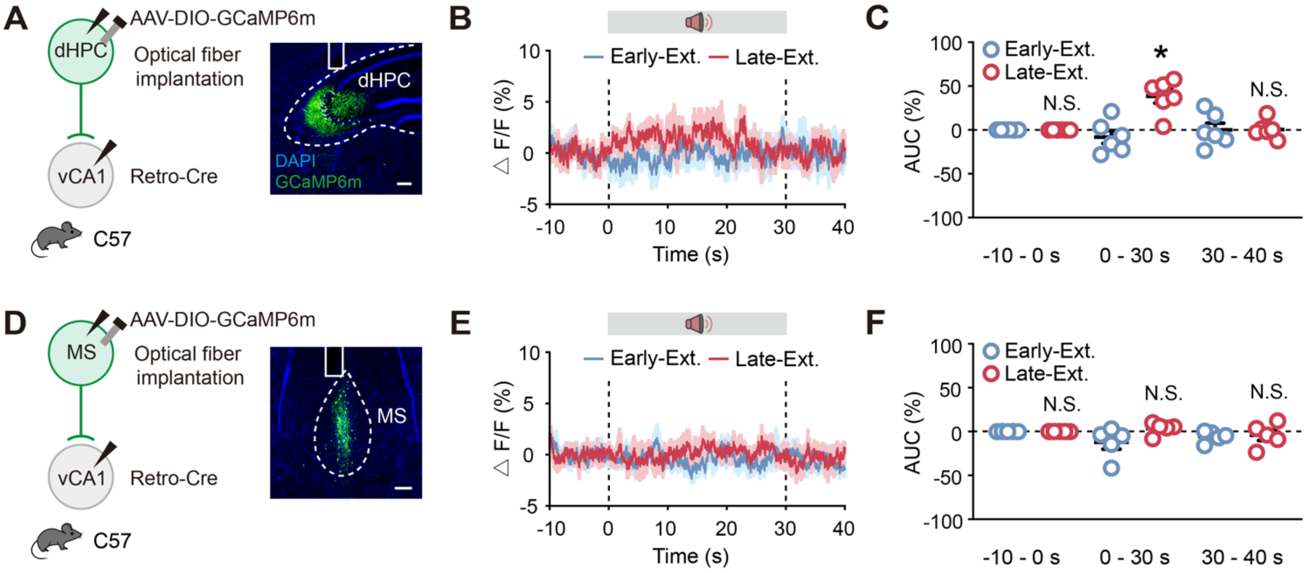

Supplemental Figure 11. Ca^{2+} recordings of dHPC-vCA1 pathway and MS-vCA1 pathway during extinction. (**A**–**C**) Ca^{2+} recording of dHPC-vCA1 pathway during extinction. Schematics of AAV injections and fiber implantation (left). Representative images of CGaMP6m expression in dHPC (right). Scale bar, 200 μm (**A**). Average calcium signals during Early-Ext. and Late-Ext. (**B**). Activity of Ca^{2+} signals (AUC) during Early-Ext. and Late-Ext. (**C**). Data are mean ± SEM. N.S., no significant difference, *$P < 0.05$. $n = 5$ mice. (**D**–**F**) Ca^{2+} recording of MS-vCA1 pathway during extinction. Schematics of AAV injections and fiber implantation (left). Representative images of CGaMP6m expression in MS (right). Scale bar, 200 μm (**D**). Average Ca^{2+} signals during Early-Ext. and Late-Ext. (**E**). AUC during Early-Ext. and Late-Ext. (**F**). Data are mean ± SEM. N.S., no significant difference. $n = 5$ mice. Paired Student's t test in (**C** and **F**).

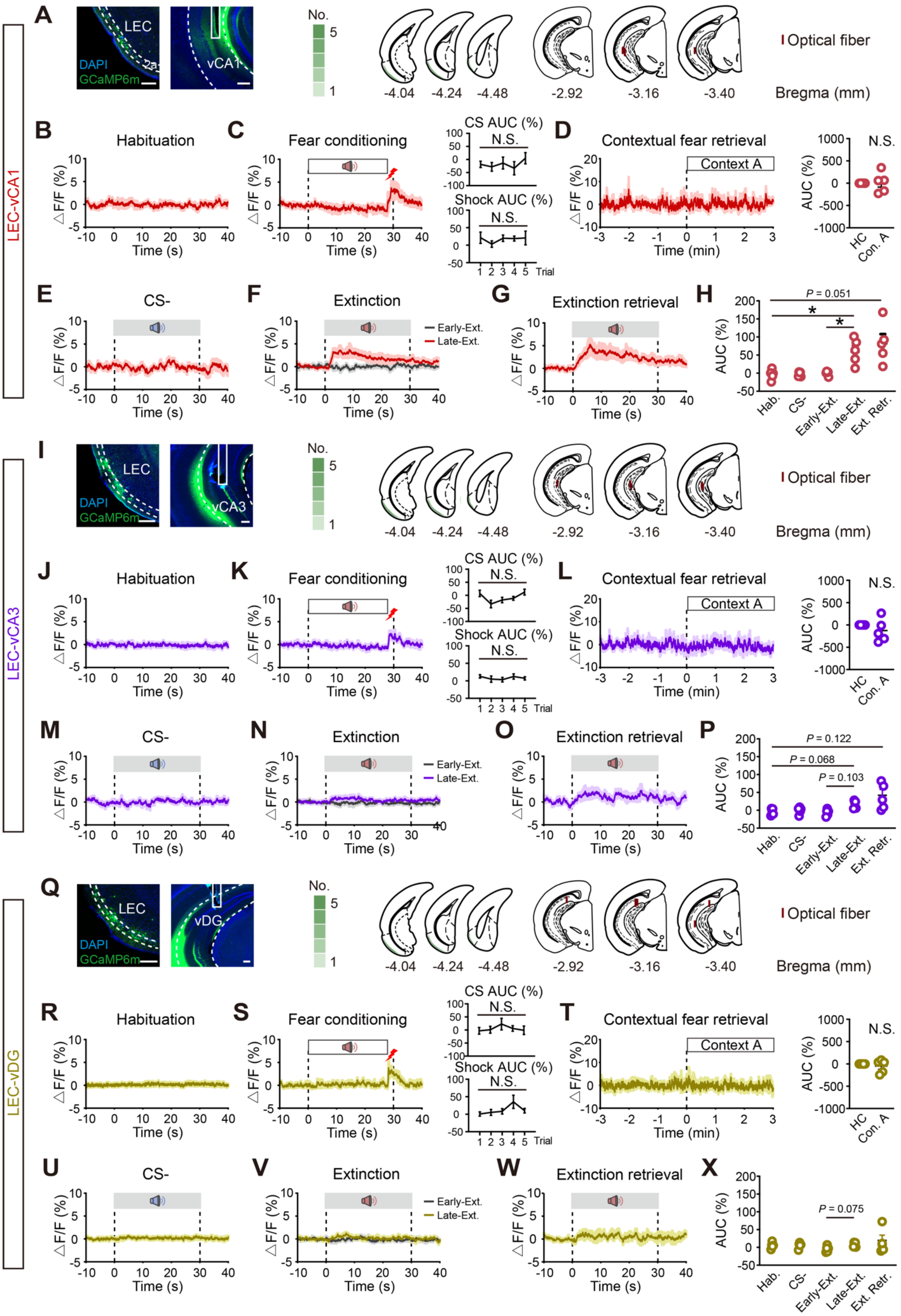

A
LEC
DAPI
GCaMP6m
vCA1
No.
5
1
-4.04
-4.24
-4.48
-2.92
-3.16
-3.40
Optical fiber
Bregma (mm)
LEC-vCA1
B
Habituation
C
Fear conditioning
CS AUC (%)
N.S.
Shock AUC (%)
N.S.
Trial
D
Contextual fear retrieval
Context A
Time (min)
AUC (%)
HC
Con. A
E
CS-
F
Extinction
Early-Ext.
Late-Ext.
G
Extinction retrieval
H
P = 0.051
*
*
Hab.
CS-
Early-Ext.
Late-Ext.
Ext. Retr.
△F/F (%)
Time (s)
I
LEC
DAPI
GCaMP6m
vCA3
LEC-vCA3
J
Habituation
K
Fear conditioning
L
Contextual fear retrieval
M
CS-
N
Extinction
O
Extinction retrieval
P
P = 0.122
P = 0.068
P = 0.103
Q
LEC
DAPI
GCaMP6m
vDG
LEC-vDG
R
Habituation
S
Fear conditioning
T
Contextual fear retrieval
U
CS-
V
Extinction
W
Extinction retrieval
X
P = 0.075

Supplemental Figure 12. Ca^{2+} recordings of the LEC-vCA1/vCA3/vDG pathway during habituation, fear conditioning, contextual fear retrieval, CS-, and extinction retrieval. (**A**) Representative image of virus expression and the axon projection targets of LEC layer 2a fan cells. Scale bar, 200 μm (left). Location of center for optical fiber lesions and virus expression for all mice (right). (**B**) Average Ca^{2+} signals during habituation. (**C**) Average Ca^{2+} signals during fear conditioning (left) and quantification of AUC of Ca^{2+} signals (right). Repeated measures one-way ANOVA: CS AUC, $F_{1.222,\ 4.888} = 0.6275$, $P = 0.4968$; Shock AUC, $F_{1.383,\ 5.531} = 0.4038$, $P = 0.6153$. N.S., no significant difference. (**D**) Average Ca^{2+} signals during contextual fear retrieval (left) and quantification of AUC of Ca^{2+} signals (right). Data are mean ± SEM. n = 5 mice. N.S., no significant difference. (**E**–**G**) Average Ca^{2+} signals during CS- (**E**), extinction training (**F**), and extinction retrieval (**G**). (**H**) Quantification of AUC of Ca^{2+} signals. Data are mean ± SEM. n = 5 mice. *$P < 0.05$. (**I**–**P**) The same as (**A**–**H**) for Ca^{2+} recordings of LEC layer 2a fan cells→vCA3 projection. *n* = 5 mice. (**Q**–**X**) The same as (**A**–**H**) for Ca^{2+} recordings of LEC layer 2a fan cells→vDG projection. (**K** and **S**) CS AUC, $F_{2.269,\ 9.074} = 2.506$, $P = 0.1326$; Shock AUC, $F_{2.148,\ 8.591} = 0.3428$, $P = 0.7331$ for (**K**). CS AUC, $F_{1.314,\ 5.257} = 0.3942$, $P = 0.6116$; Shock AUC, $F_{1.334,\ 5.336} = 1.558$, $P = 0.2779$ for (**S**). N.S., no significant difference. *n* = 5 mice. Repeated measures one-way ANOVA and one-sample *t* test with hypothetical mean of zero in (**C**, **K** and **S**), paired Student's *t* test in (**D**, **L** and **T**) and repeated measures one-way ANOVA with Tukey's multiple comparisons test in (**H**, **P** and **X**).

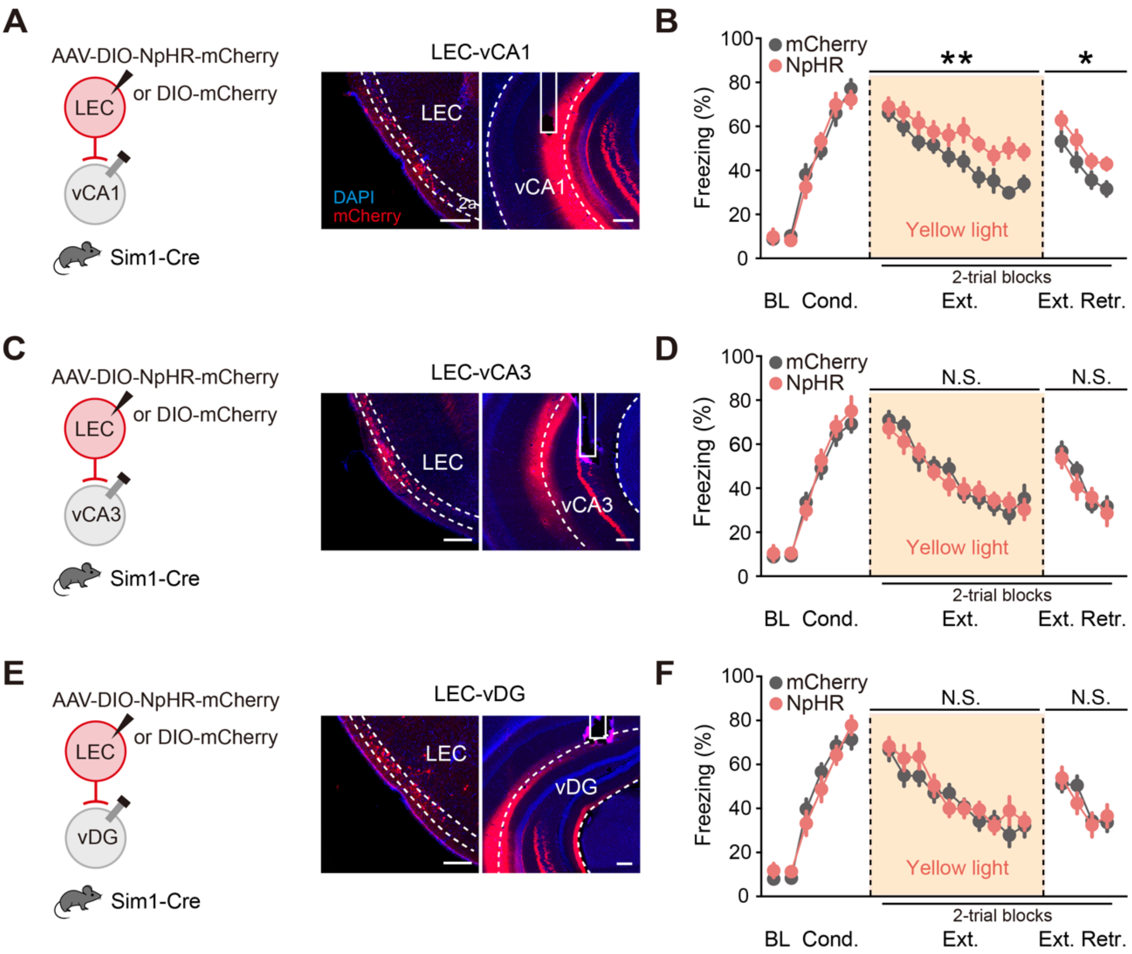

Supplemental Figure 13. Effects of manipulating LEC layer 2a fan cells-vCA1/vCA3/vDG pathway on fear extinction. (**A**, **C** and **E**) Schematics of AAV injections and representative images of virus expression. Scale bar, 200 μm. (**B**) Effect of inhibiting LEC layer 2a fan cells→vCA1 projection on extinction training and retrieval. Time courses of freezing responses to the CS during fear conditioning, extinction training, and extinction retrieval sessions. Statistics are as follows: main effect of AAV, conditioning, $F_{1,14} = 0.06277$, $P = 0.8058$; extinction training, $F_{1,14} = 9.633$, $P = 0.0078$; extinction retrieval, $F_{1,14} = 5.694$, $P = 0.0317$. $n = 8$ mice per group. Data are mean ± SEM. *$P < 0.05$, **$P < 0.01$. (**D**) Effect of inhibiting LEC layer 2a fan cells→vCA3 projection on extinction training and retrieval. Time courses of freezing responses to the CS during fear conditioning, extinction training and extinction retrieval sessions. Statistics are as follows: main effect of AAV, conditioning, $F_{1,14} = 0.4246$, $P = 0.5252$; extinction training, $F_{1,14} = 0.1204$, $P = 0.7337$; extinction retrieval, $F_{1,14} = 0.2503$, $P = 0.6246$. $n = 8$ mice per group. Data are mean ± SEM. N.S., no significant difference. (**E**) Effect of inhibiting LEC layer 2a fan cells→vDG projection on extinction training and retrieval. Time courses of freezing responses to the CS during fear conditioning, extinction training and extinction retrieval sessions. Statistics are as follows: main effect of AAV, conditioning, $F_{1,13} = 0.1988$, $P = 0.6630$; extinction training, $F_{1,13} = 0.8019$, $P = 0.3868$; extinction retrieval, $F_{1,13} = 0.05742$, $P = 0.8144$. mCherry group, $n = 8$ mice, NpHR group, $n = 7$ mice. Data are mean ± SEM. N.S., no significant difference. Repeated measures two-way ANOVA in (**B**, **D** and **F**).

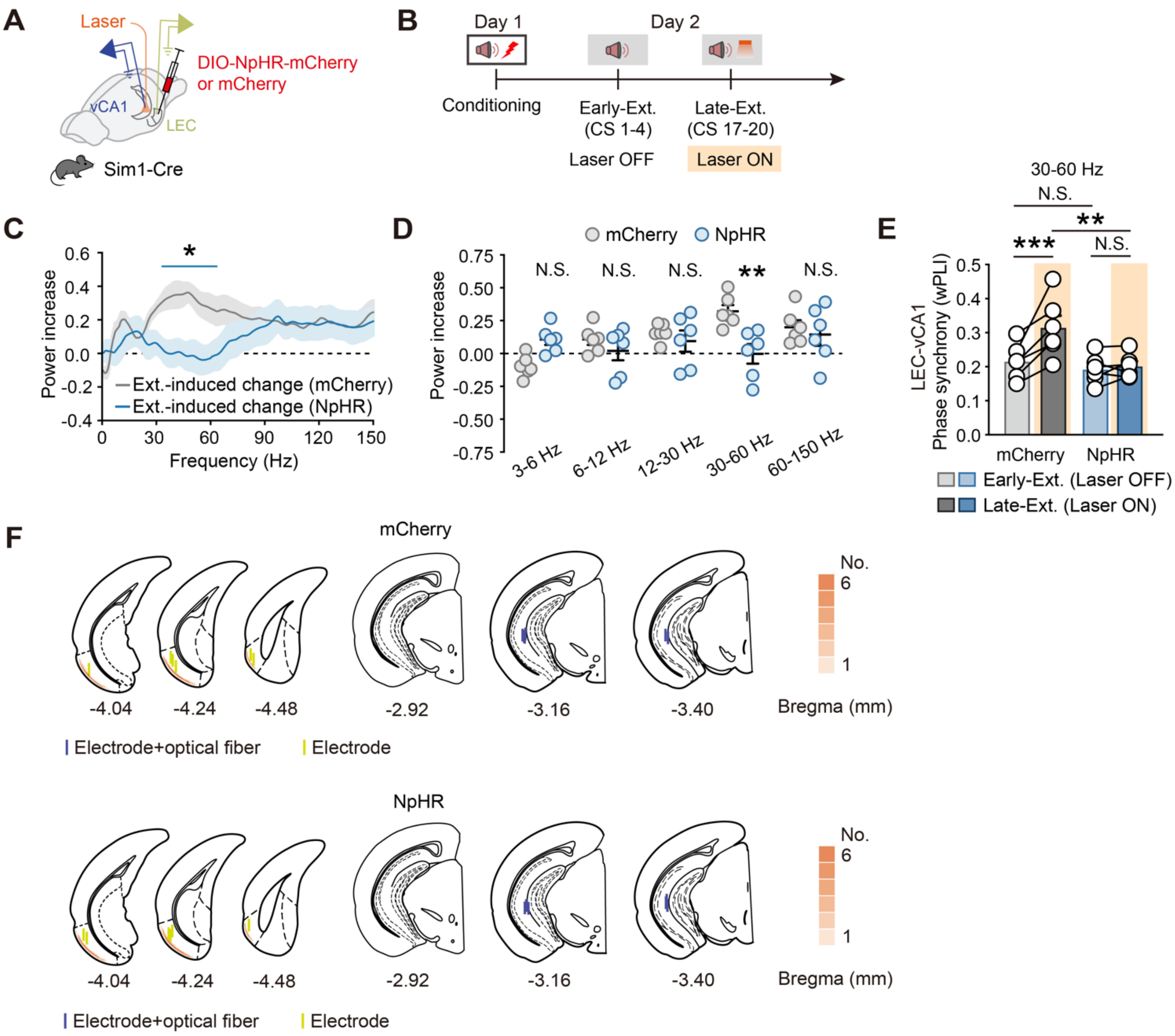

Supplemental Figure 14. LEC-vCA1 projections promote network oscillations associated with fear extinction. (**A** and **B**) Schematics of stereotaxic surgery (**A**) and experimental design (**B**). Light for optogenetic inhibition was delivered during Late-Ext. on Day 2. (**C**) Extinction-induced changes for power spectrum of vCA1 LFP recordings. Mean ± SEM of power (Late-Ext. - Early-Ext.) / (Late-Ext. + Early-Ext.). $n = 6$ mice per group. Blue line indicates frequencies with a significant effect. *$P < 0.05$ with Bonferroni correction for multiple comparisons. (**D**) Average power increase of vCA1 LFP recordings. Data are mean ± SEM and circles denote individual mice. $n = 6$ mice per group. Statistics are as follows: main effect of AAV, $F_{1,10} = 0.9022$, $P = 0.3646$. N.S., no significant difference, **$P < 0.01$. (**E**) Low-gamma phase synchrony quantified using the wPLI between LEC and vCA1 LFPs. Histograms represent mean ± SEM and circles denote individual mice. $n = 6$ mice per group. N.S., no significant difference, **$P < 0.01$, ***$P < 0.001$, light × group interaction, $F_{1,10} = 10.98$, $P = 0.0078$. (**F**) Location of center for LFP electrode and optical fiber lesions for all mice. Wilcoxon signed-rank test with Bonferroni correction for multiple comparisons in (**C**), repeated measures two-way ANOVA and unpaired Student's *t* test in (**D**) and repeated measures two-way ANOVA with Sidak's multiple comparisons test in (**E**).

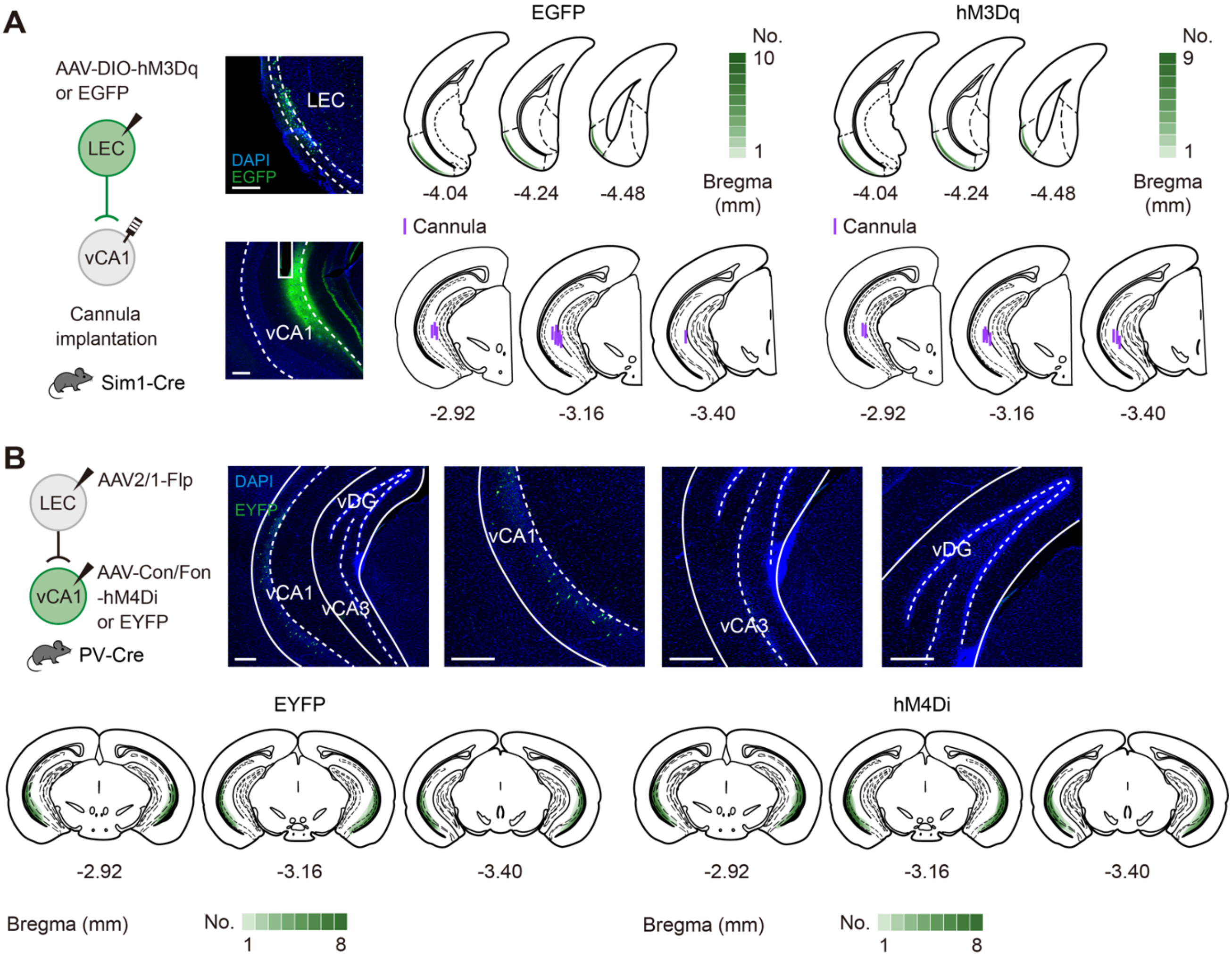

Supplemental Figure 15. Histological validation of the virus, optical fiber and cannula location. (**A**) Schematics of AAV injections and representative images of virus expression (left). Scale bar, 200 µm. Location of center for cannula implantation and virus expression for all mice (right). (**B**) Schematics of AAV injections and representative images of virus expression (top). Scale bar, 200 µm. Location of virus expression for all mice (bottom).

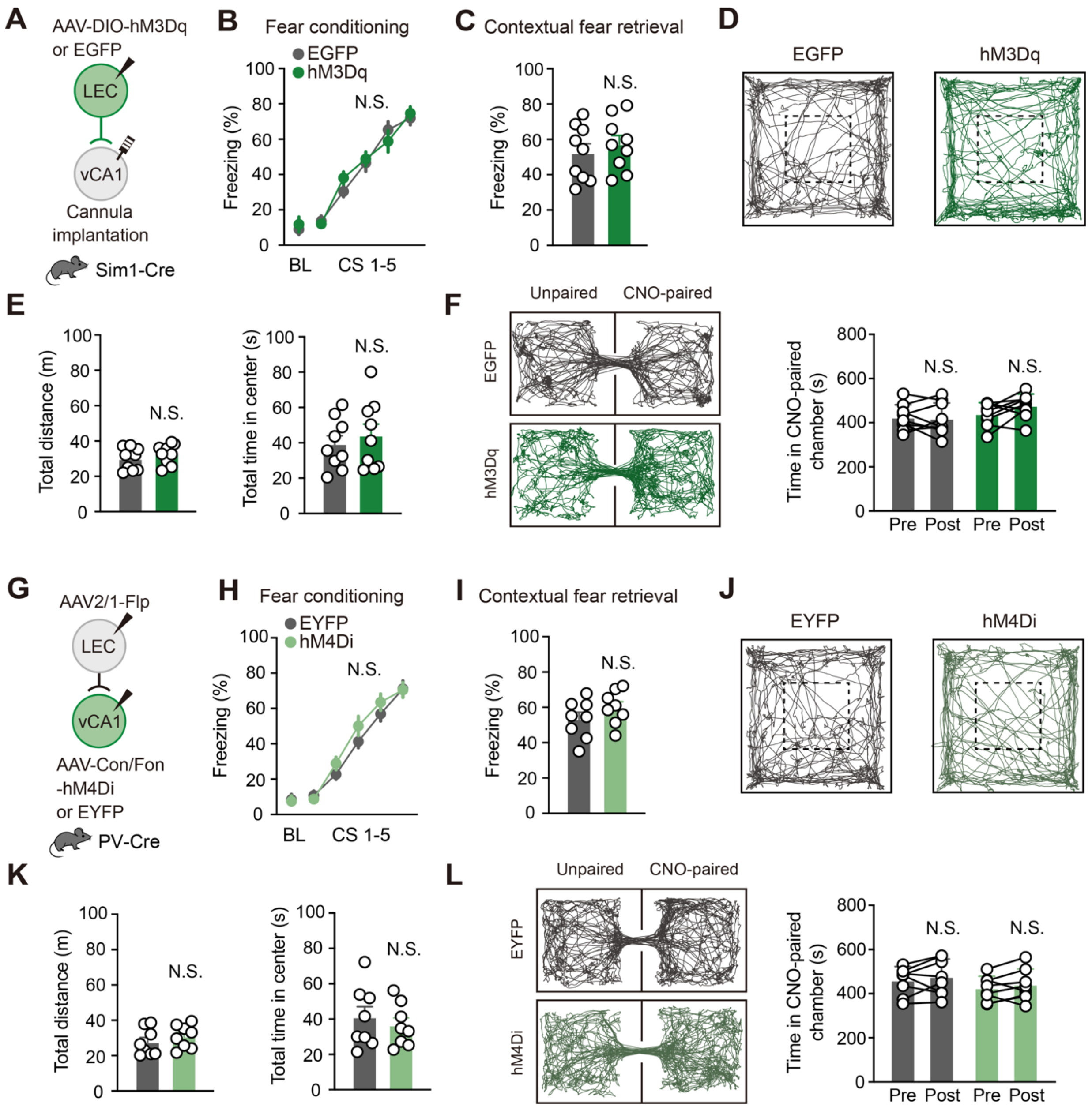

Supplemental Figure 16. Effects of manipulating LEC-layer 2a→vCA1 projection on fear conditioning, contextual fear retrieval, and non-task related place preference assays. (**A**) Schematics of AAV injections and experimental design for activation of LEC-layer 2a→vCA1 projection. (**B**) Time courses of freezing responses during baseline and fear conditioning. Statistics are as follows: main effect of AAV, conditioning, $F_{1,16} = 0.04578$, $P = 0.8333$; $n = 9$ mice per group. Data are mean ± SEM. N.S., no significant difference. (**C**) Freezing responses during contextual fear retrieval. Data are mean ± SEM. N.S., no significant difference. (**D** and **E**) Effect of manipulating LEC-layer 2a→vCA1 projection on locomotor activity in the open field test (OFT). (**D**) Example traces of open field test. (**E**) Total distances travelled and total time in center in the open field. Data are mean ± SEM. N.S., no significant difference. (**F**) Effect of manipulating LEC-layer 2a→vCA1 projection on conditioned place aversion (CPA) or preference (CPP). Representative traces of post conditioning (left) and individual data of the durations of time spent in the CNO-paired chamber (right). Data are mean ± SEM. N.S., no significant difference. $n = 9$ mice per group. (**G**–**L**) The same as (**A**–**F**) for inhibition

of LEC-layer 2a→vCA1 projection. n = 8 mice per group. Statistics are as follows: (**H**) main effect of AAV, conditioning, $F_{1,14}$ = 1.144, P = 0.3029; Data are mean ± SEM. N.S., no significant difference. Repeated measures two-way ANOVA with Sidak's multiple comparisons test in (**B** and **H**), unpaired Student's t test in (**C**, **E**, **I** and **K**) and paired Student's t test in (**F** and **L**).

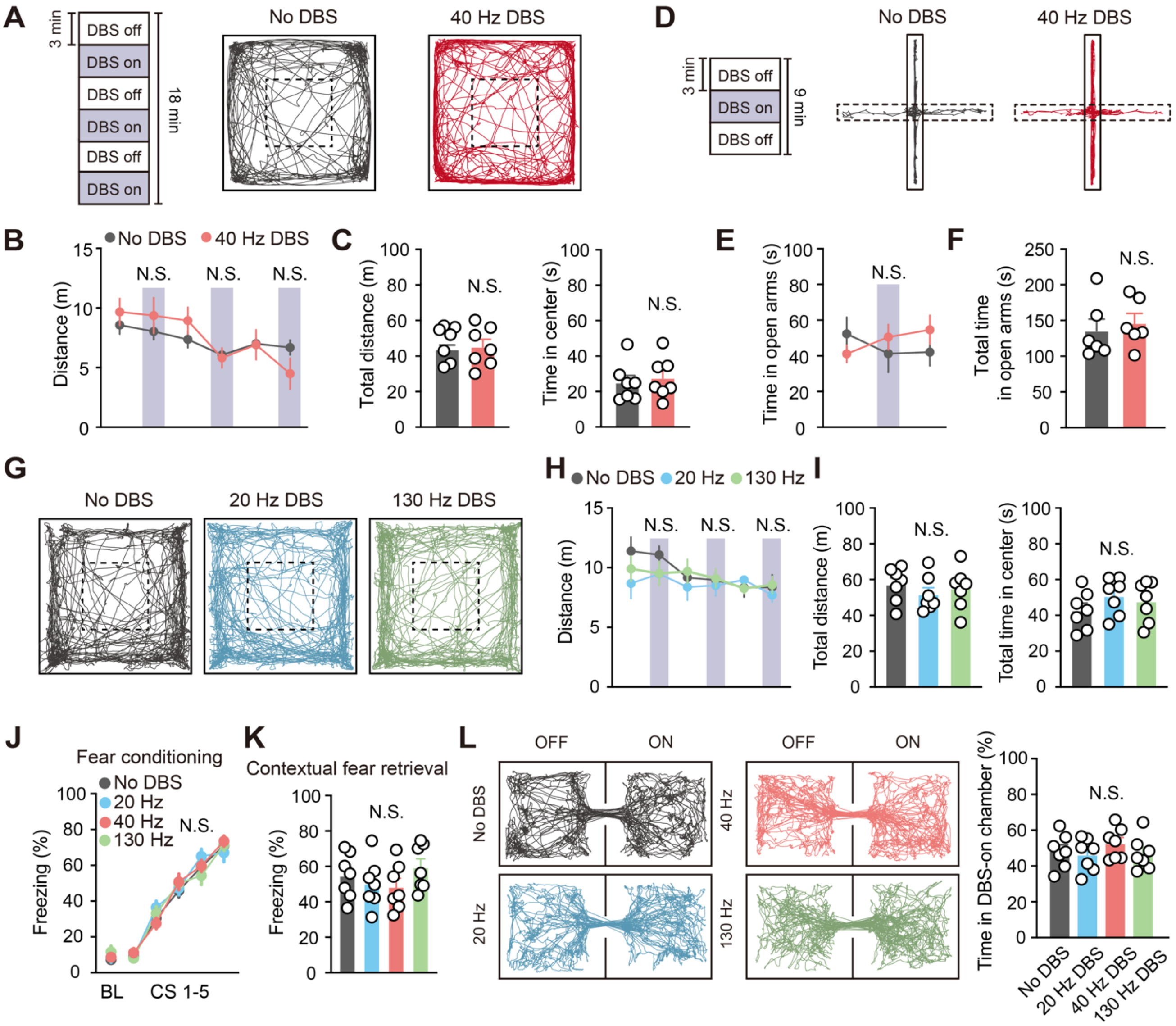

Supplemental Figure 17. Effects of vCA1 DBS on fear conditioning, contextual fear retrieval, and non-task related place preference assays. (**A** and **D**) Schematics of experimental design of the locomotor and anxiety-related activity for low-gamma vCA1 DBS. (**B**) Effect of low-gamma vCA1 DBS on locomotor activity in OFT. The distance traveled in a 3-min DBS-on (purple box) and DBS-off period. (**C**) Total distances travelled (left) and total time in center (right) in OFT for No DBS group and DBS group. Data are mean ± SEM. N.S., no significant difference (left). n = 7 mice per group. (**E**) Effect of low-gamma vCA1 DBS on anxiety-related activity in the elevated-plus maze (EPM). The time spent exploring open arms in a 3-min DBS-on (purple box) and DBS-off period. (**F**) Total time spent exploring open arms. Data are mean ± SEM. N.S., no significant difference. n = 7 mice per group. (**G**–**I**) The same as (**A**–**C**) for 20 Hz and 130 Hz vCA1 DBS. n = 7 mice per group. Data are mean ± SEM. N.S., no significant difference. (**J**) Time courses of freezing responses during baseline and fear conditioning. Statistics are as follows: main effect of AAV, conditioning, $F_{3,24} = 0.2206$, $P = 0.8811$. N.S., no significant difference. (**K**) Freezing responses during contextual fear retrieval. N.S., no significant difference. (**L**) Effect of manipulating LEC-layer 2a→vCA1 projection on real-time place aversion (RTPA) or preference (RTPP). Representative traces (left) and individual data of the durations of time spent in the DBS-on chamber. n = 7 mice per group. Data are mean ± SEM. N.S., no significant

difference. Repeated measures two-way ANOVA with Sidak's multiple comparisons test in (**B**, **E**, and **H**), unpaired Student's *t* test in (**C** and **F**) and one-way ANOVA with Tukey's multiple comparisons test in (**I**, **K**, and **L**).

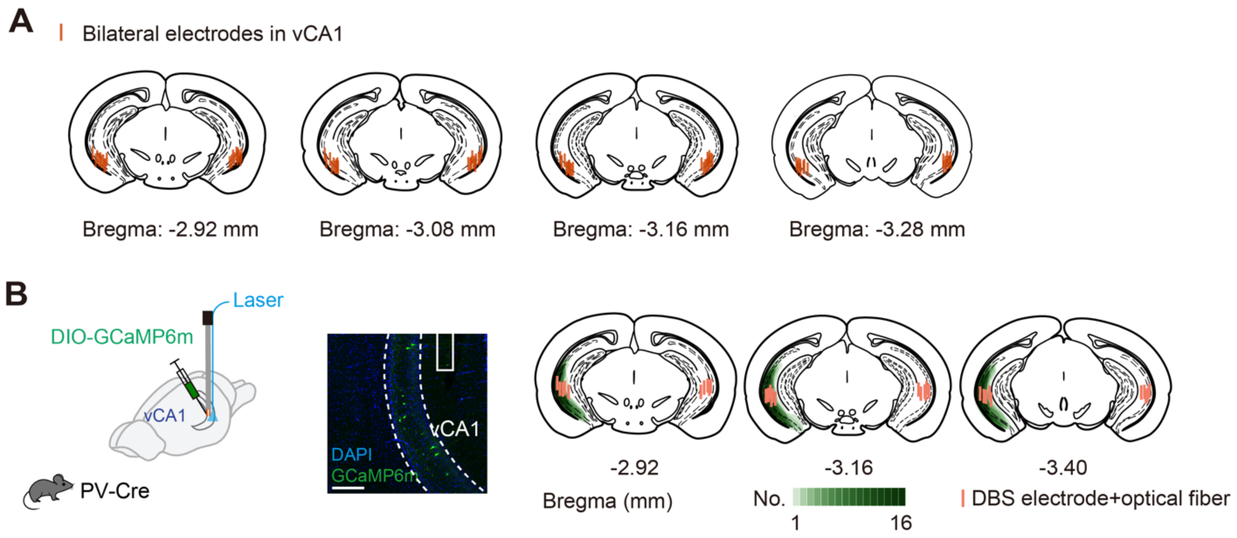

Supplemental Figure 18. Histological validation of the virus, optical fiber, and DBS electrode location. (**A**) Location of center for DBS electrode lesions for all mice. (**B**) Schematics of AAV injections and representative images of virus expression (left). Scale bar, 200 μm. Location of center for DBS electrode, optical fiber lesions and virus expression for all mice (right).

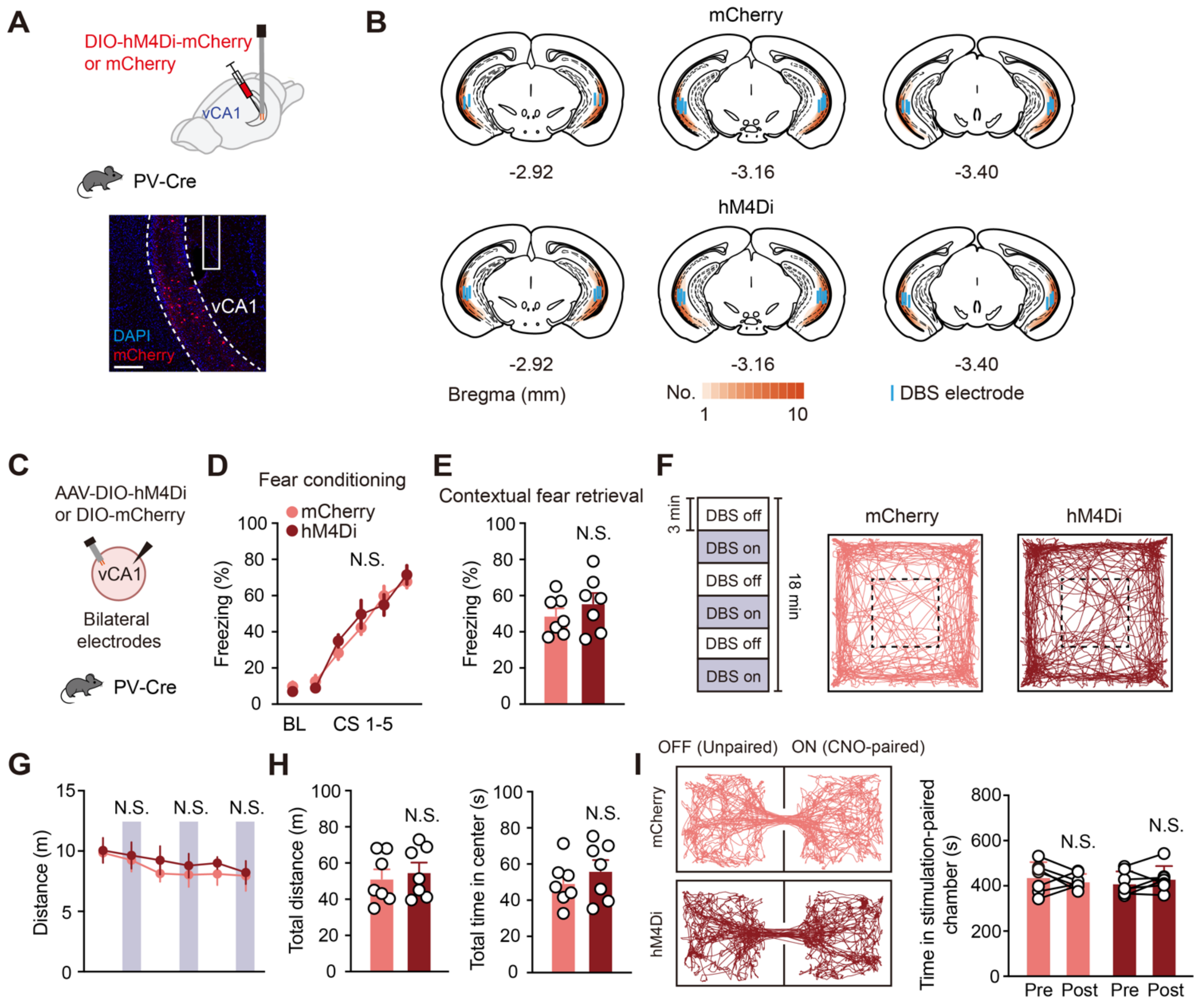

Supplemental Figure 19. Effects of vCA1 DBS in combination with chemogenetic inhibition of vCA1 PV-INs on fear conditioning, contextual fear retrieval, and non-task related place preference assays. (**A** and **B**) Schematics of AAV injections and representative images of virus expression (**A**). Scale bar, 200 μm. Location of center for DBS electrode and virus expression for all mice (**B**). (**C**) Schematics of AAV injections and experimental design. (**D**) Time courses of freezing responses during baseline and fear conditioning. Statistics are as follows: main effect of AAV, conditioning, $F_{1,12} = 0.2734$, $P = 0.6106$. N.S., no significant difference. (**E**) Freezing responses during contextual fear retrieval. Data are mean ± SEM. N.S., no significant difference. (**F–H**) Effect of low-gamma DBS-vCA1 with vCA1 PV-INs inhibition on locomotor activity in OFT. (**F**) Example traces of open field test. (**G** and **H**) Total distances travelled and total time in center in the open field. Data are mean ± SEM. N.S., no significant difference. (**I**) Effect of low-gamma DBS-vCA1 with vCA1 PV-INs inhibition on conditioned place aversion or preference. Representative traces of post conditioning (left) and individual data of the durations of time spent in the stimulation-paired chamber (right). Data are mean ± SEM. N.S., no significant difference. *n* = 7 mice per group. Repeated measures two-way ANOVA with Sidak's multiple comparisons test in (**D** and **G**), unpaired Student's *t* test in (**E** and **H**) and paired Student's *t* test in (**I**).

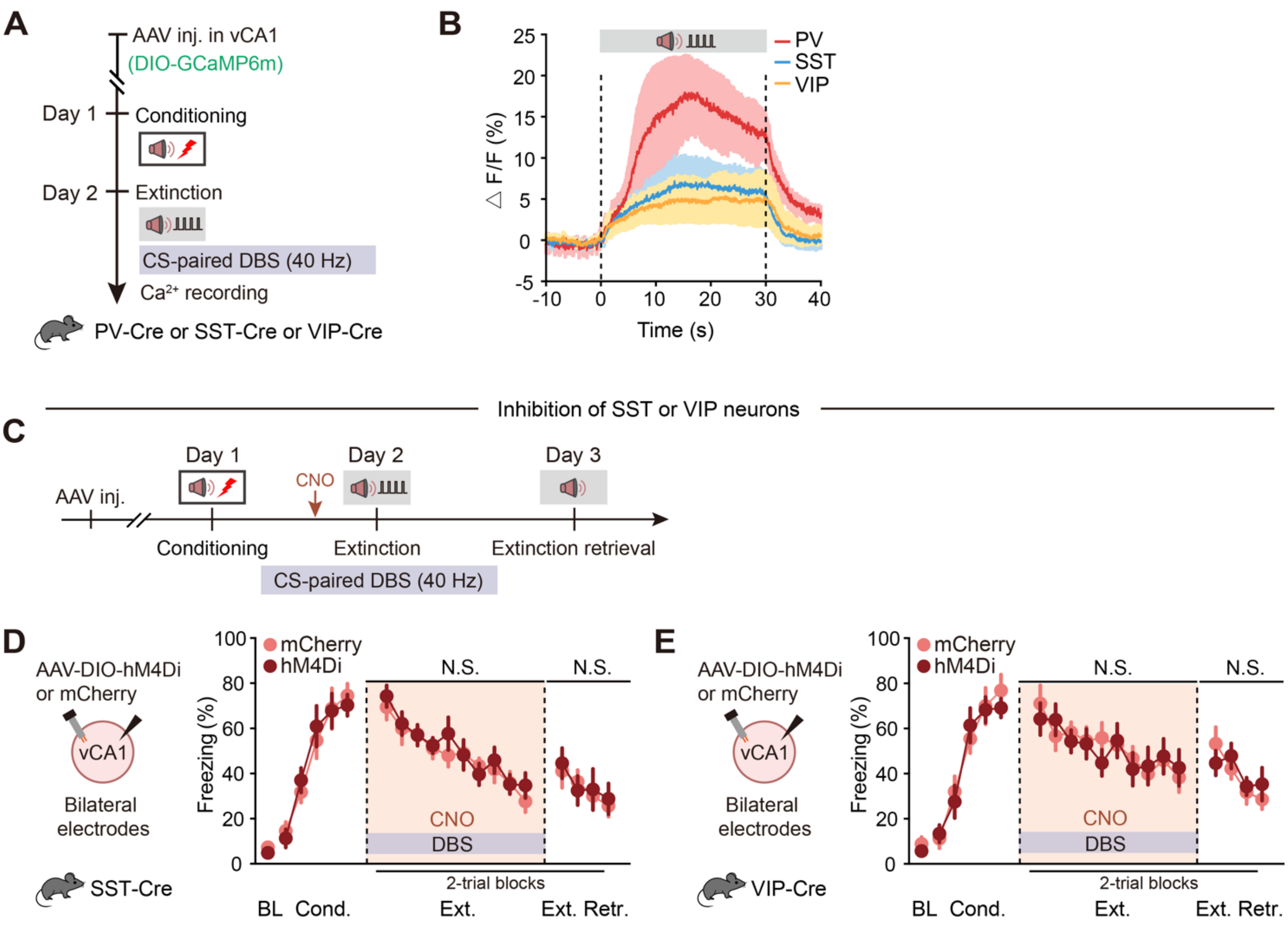

Supplemental Figure 20. No effects of inhibition of SST-INs or VIP-INs on low-gamma DBS-induced extinction promotion. (**A** and **C**) Schematics of AAV injections and experimental design. (**B**) Average Ca^{2+} signals in different INs during 40 Hz DBS-paired extinction training. Data are mean ± SEM. PV group, *n* = 4 mice; SST group, *n* = 4 mice; VIP group, *n* = 5 mice. (**D** and **E**) Effect of inhibiting vCA1 SST-INs (**D**) or VIP-INs (**E**) on DBS-induced extinction promotion. Schematics of AAV injections (left). Time courses of freezing responses to the CS during fear conditioning, extinction training and extinction retrieval sessions (right). Statistics are as follows: main effect of AAV, (**D**) conditioning, $F_{1,12} = 0.0081$, $P = 0.9297$; extinction training, $F_{1,12} = 0.6232$, $P = 0.4452$; extinction retrieval, $F_{1,12} = 0.0374$, $P = 0.8499$. mCherry group, *n* = 7 mice; hM4Di group, *n* = 7 mice. (**E**) conditioning, $F_{1,12} = 0.0460$, $P = 0.8339$; extinction training, $F_{1,12} = 0.0661$, $P = 0.8015$; extinction retrieval, $F_{1,12} = 0.0788$, $P = 0.7837$. mCherry group, *n* = 7 mice; hM4Di group, *n* = 7 mice. Data are mean ± SEM. N.S., no significant difference. Repeated measures two-way ANOVA in (**D** and **E**).

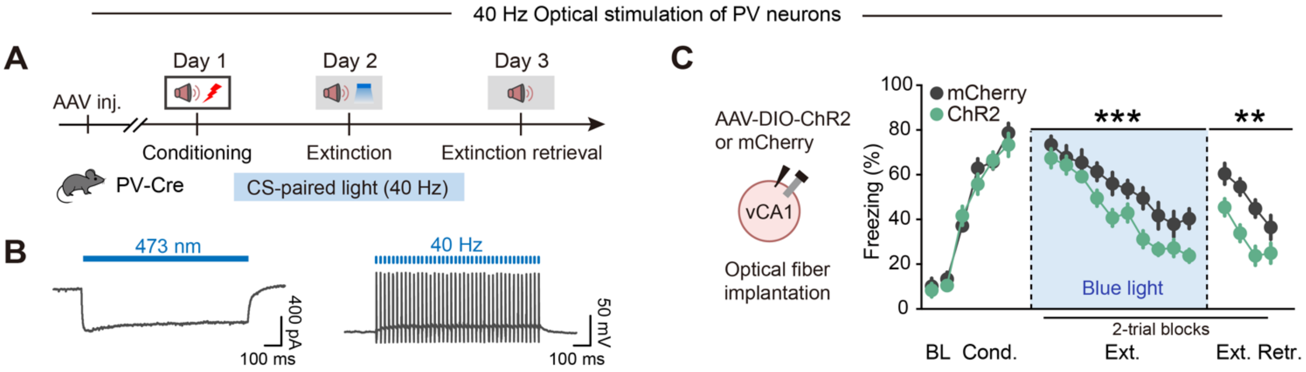

Supplemental Figure 21. Effects of 40 Hz optical stimulation of PV-INs on extinction training and retrieval. (**A**) Experimental design of 40 Hz optical stimulation of vCA1 PV-INs. CS is paired with 40 Hz blue light during extinction training. (**B**) Activation effect of blue light on ChR2-expressing vCA1 PV-INs. Representative trace of ChR2-mediated current by 1-s pulses of blue light in the voltage-clamp mode (left). Action potentials were evoked in a ChR2-expressing vCA1 PV-INs in current-clamp mode by 1-ms pulses of photostimuli at 40 Hz (right). (**C**) Effects on optical activating vCA1 PV-INs on extinction training and extinction retrieval. Schematics of AAV injections (left). Time courses of freezing responses to the CS during fear conditioning, extinction training and extinction retrieval sessions (right). Statistics are as follows: main effect of AAV, conditioning, $F_{1,16} = 0.6004$, $P = 0.4497$; extinction training, $F_{1,16} = 17.04$, $P = 0.0008$; extinction retrieval, $F_{1,16} = 15.35$, $P = 0.0012$. mCherry group, n = 9 mice, ChR2 group, n = 9 mice. Data are mean ± SEM. N.S., no significant difference, $^{**}P < 0.01$, $^{***}P < 0.001$. Repeated measures two-way ANOVA in (**C**).

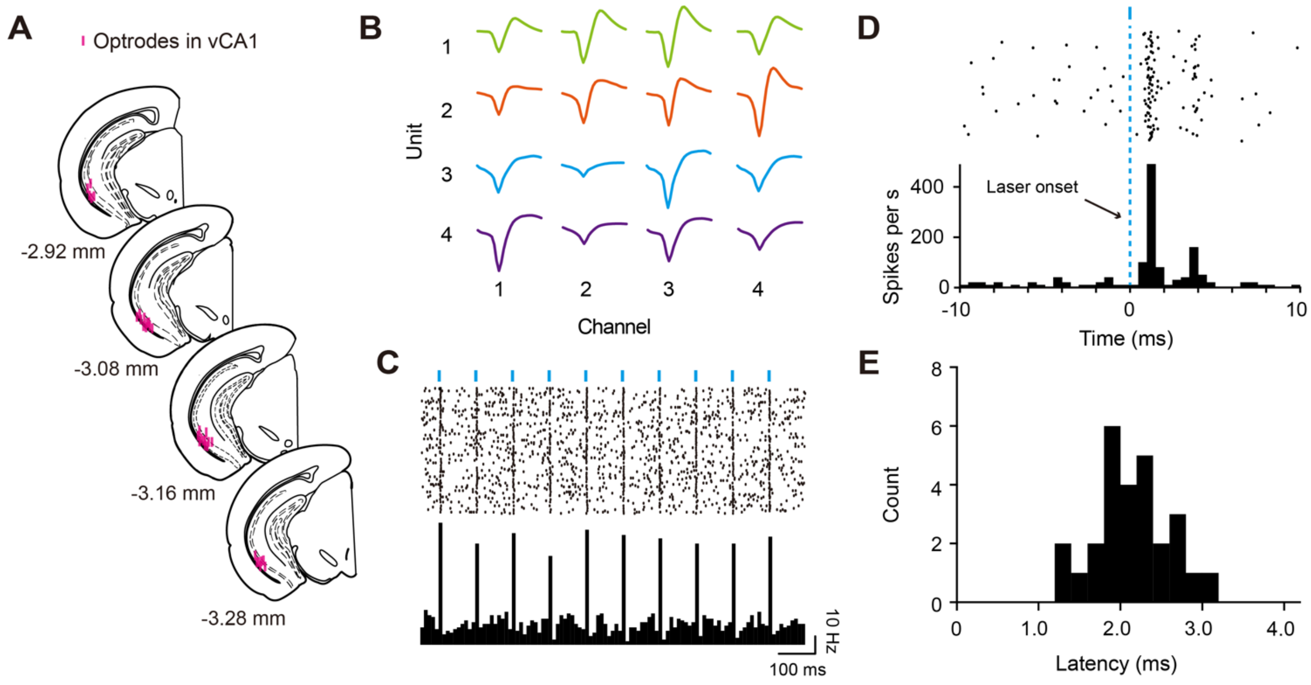

Supplemental Figure 22. Single-unit recording of vCA1. (**A**) Optrode placement in vCA1 for optrode recording experiments. (**B**) An example spike sorting result from a single tetrode in vCA1. (**C**) Raster plot (top) and PSTH (bottom) for light-evoked spikes of an example tagged PV-IN. Blue bars mark light pulses (1-ms, 10 Hz). (**D**) Sample of peri-event raster plots and PSTH for light-evoked spike latency around 2 ms (left). Plot of latency distribution showing short latencies of light-evoked spike for all ChR2-tagged PV-INs in vCA1 (right). (**E**) Classification of recorded vCA1 neurons into WS putative pyramidal cells (blue circles), NS-nonFS (gray circles), Tagged PV (red circles) and FS-PV (orange circles) based on peak-to-trough latency and baseline firing rate.

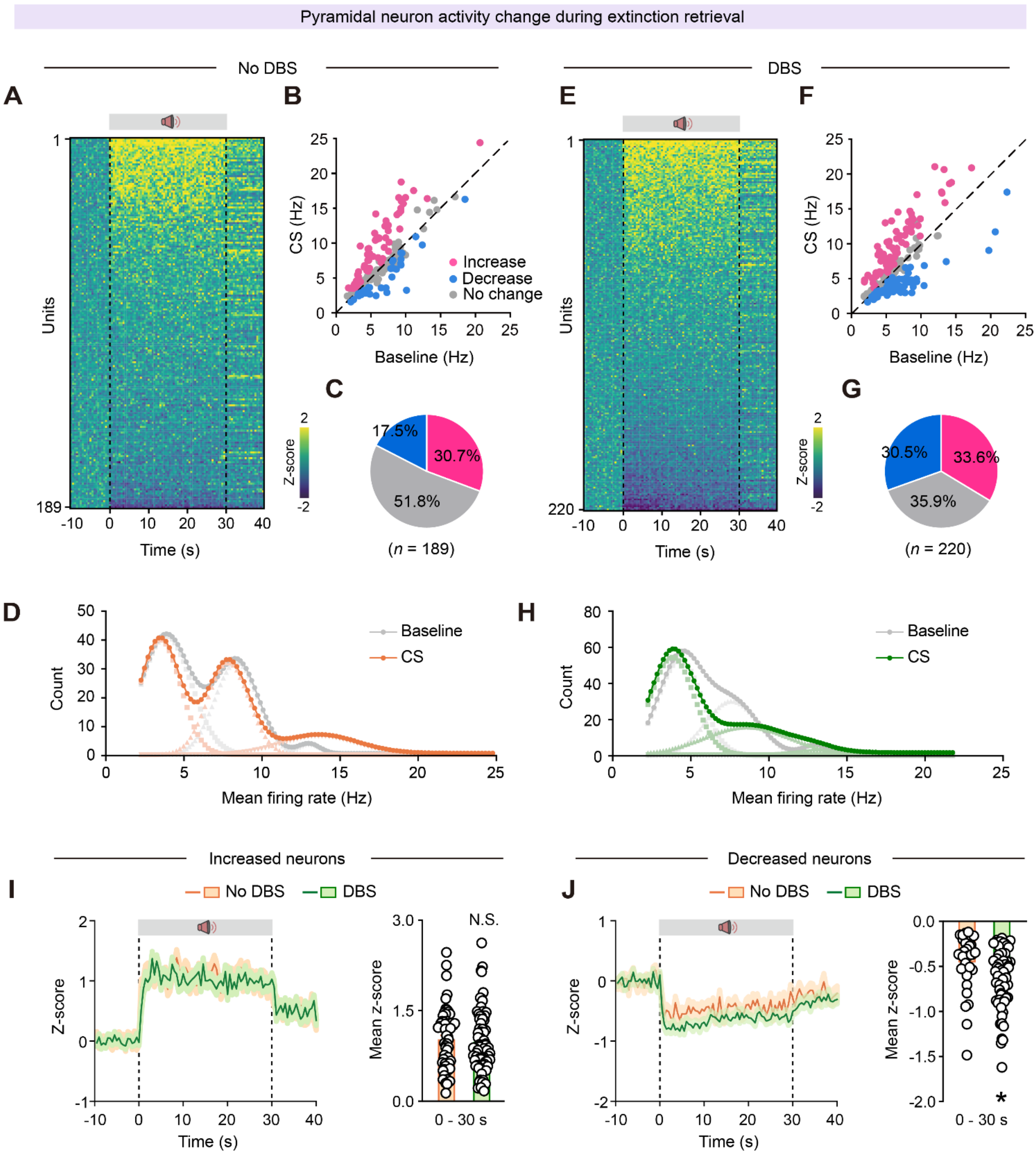

Supplemental Figure 23. Low-gamma DBS paired extinction training induces sustained inhibition of vCA1 pyramidal neurons during extinction retrieval. (**A** and **E**) Heatmaps showing responses of pyramidal neurons during extinction retrieval. (**A**) No DBS manipulation during extinction training. (**E**) CS is paired with 40 Hz DBS during extinction training. (**B** and **F**) Correlation of firing rate during Baseline and CS for individual pyramidal neurons. (**B**) No DBS manipulation during extinction training. (**F**) CS is paired with 40 Hz DBS during extinction training. Colored circles indicate neurons that showed significant firing rate increase (pink) or decrease (blue) or no significant difference (gray). (**C** and **G**) The pie graphs show the percentage of neurons that had significantly higher, lower, or unchanged firing rates during extinction retrieval. (**D** and **H**) Distribution of mean

firing rate during Baseline and CS for individual pyramidal neurons. (**D**) No DBS manipulation during extinction training. Gray lines indicate the distribution of mean firing rate during Baseline. Orange lines indicate the distribution of mean firing rate during CS. (**H**) CS is paired with 40 Hz DBS during extinction training. Gray lines indicate the distribution of mean firing rate during Baseline. Green lines indicate the distribution of mean firing rate during CS. (**I** and **J**) Z-scored signal changes of increased (**I**) and decreased (**J**) responses of pyramidal neurons during extinction retrieval. Orange lines indicate No DBS manipulation during extinction training and green lines indicate 40 Hz DBS manipulation during extinction training. Data are mean ± SEM. N.S., no significant difference, $^*P < 0.05$. Unpaired Student's *t* test in (**I** and **J**).

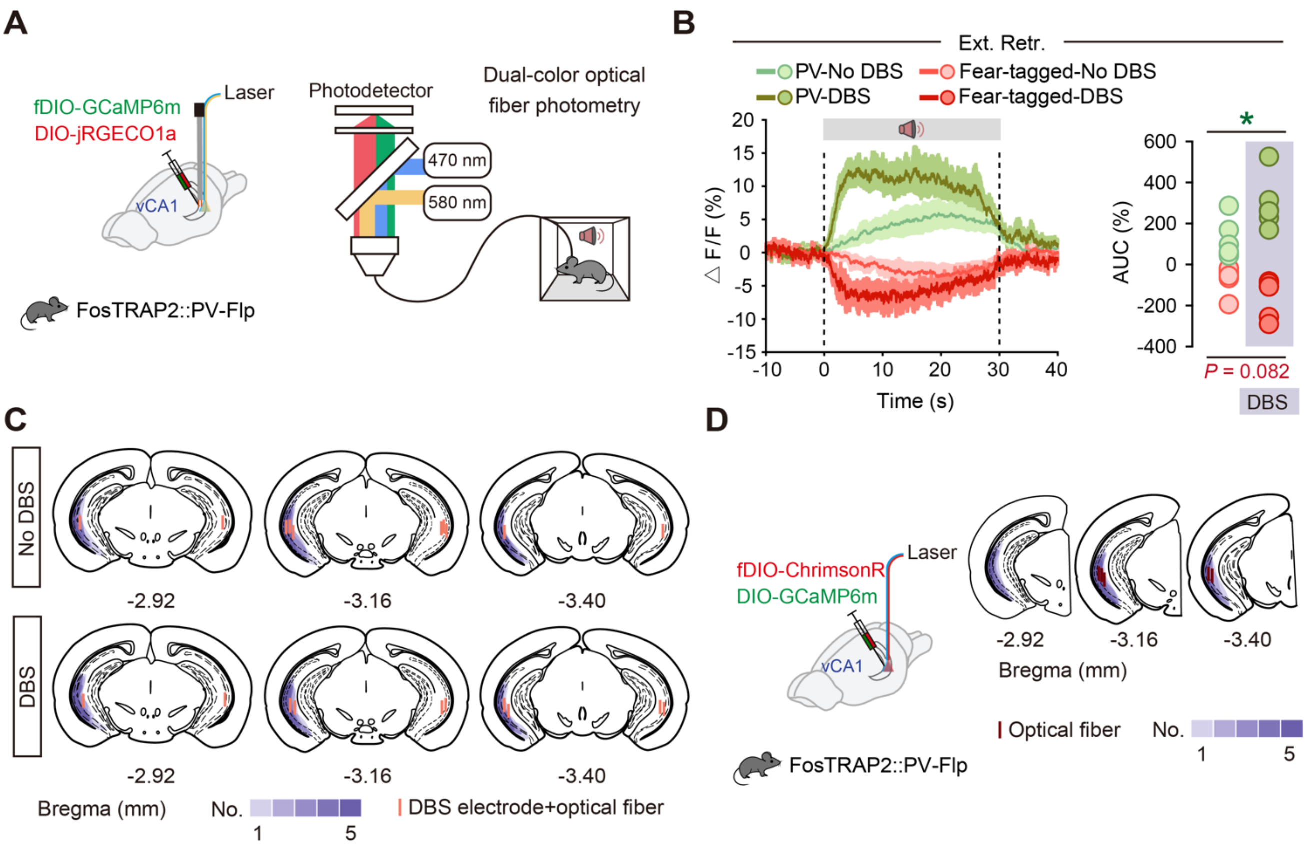

Supplemental Figure 24. Low-gamma DBS paired extinction training engages vCA1 PV-INs to suppress fear-tagged neurons during extinction retrieval. (**A**) Schematics of AAV injection and experimental design. (**B**) Average Ca^{2+} signals in PV-INs and fear-tagged neurons during extinction retrieval. Data are mean ± SEM. *$P < 0.05$. $n = 6$ mice per group. (**C**) Location of center for DBS electrode, optical fiber lesions and virus expression for all mice, related to Figure 7, A–D. (**D**) Location of center for optical fiber lesions and virus expression for all mice, related to Figure 7, E and F. Unpaired Student's *t* test in (**B**).

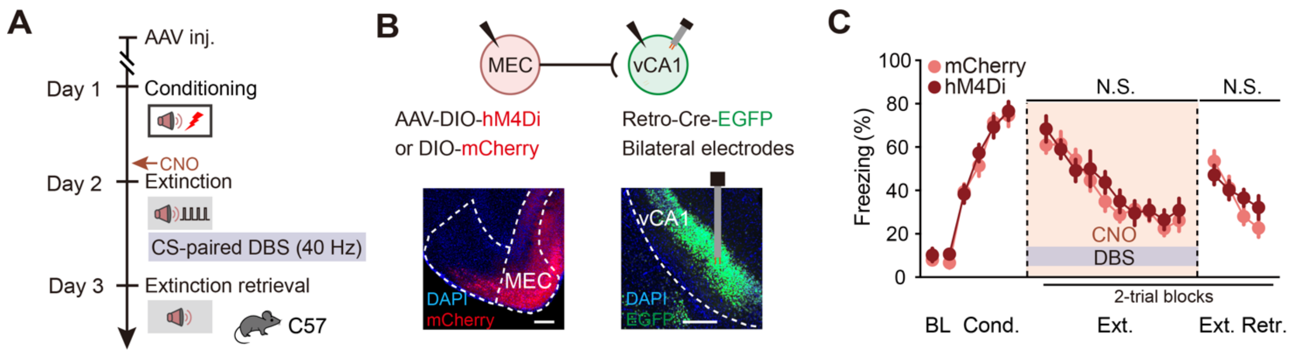

Supplemental Figure 25. Inhibition of MEC-vCA1 had no effect on DBS-induced extinction promotion. (**A**) Schematics of experimental design. CS is paired with 40 Hz DBS during extinction training and CNO was administrated 30 min (i.p.) before extinction training. (**B**) Schematics of AAV injections (top) and representative images of virus expression (bottom). Scale bar, 200 µm. (**C**) Effect of inhibiting MEC-vCA1 projectors on DBS-induced extinction promotion. Time courses of freezing responses to the CS during fear conditioning, extinction training and extinction retrieval sessions. Statistics are as follows: main effect of AAV, conditioning, $F_{1,14} = 0.4088$, $P = 0.5329$; extinction training, $F_{1,14} = 0.4952$, $P = 0.4932$; extinction retrieval, $F_{1,14} = 0.2920$, $P = 0.5974$. mCherry group, $n = 8$ mice; hM4Di group, $n = 8$ mice. Data are mean ± SEM. N.S., no significant difference. Repeated measures two-way ANOVA in (**C**).

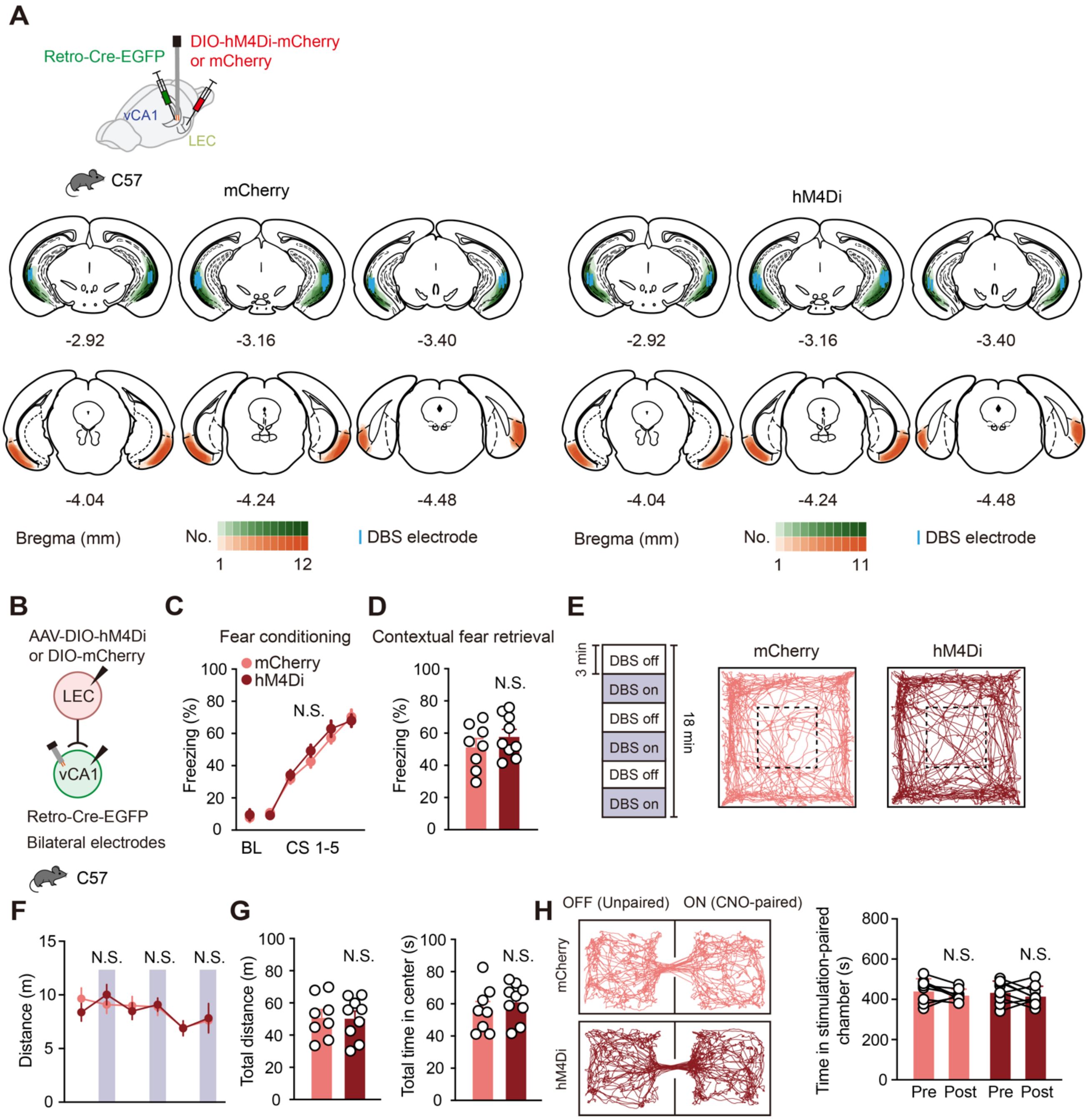

Supplemental Figure 26. Effects of low-gamma stimulation of the LEC→vCA1 circuit on fear conditioning, contextual fear retrieval, and non-task related place preference assays. (**A**) Location of center for DBS electrode and virus expression for all mice. (**B**) Schematics of AAV injections and experimental design. (**C**) Time courses of freezing responses during baseline and fear conditioning. Statistics are as follows: main effect of AAV, conditioning, $F_{1,15} = 0.5789$, $P = 0.4585$. N.S., no significant difference. (**D**) Freezing responses during contextual fear retrieval. Data are mean ± SEM. N.S., no significant difference. (**E–G**) Effect of low-gamma vCA1 DBS with LEC-vCA1 inhibition on locomotor activity in OFT. (**E**) Example traces of open field test. (**F** and **G**) Total distances travelled and total time in center in the open field. Data are mean ± SEM. N.S., no significant difference. (**H**) Effect of low-gamma vCA1 DBS with LEC-vCA1 inhibition on CPA or CPP. Representative traces of post conditioning (left) and individual data of the durations of time spent in the stimulation-paired

chamber (right). Data are mean ± SEM. N.S., no significant difference. mCherry group, $n = 8$ mice, hM4Di group, $n = 9$ mice. Repeated measures two-way ANOVA with Sidak's multiple comparisons test in (**C** and **F**), unpaired Student's *t* test in (**D** and **G**) and paired Student's *t* test in (**H**).

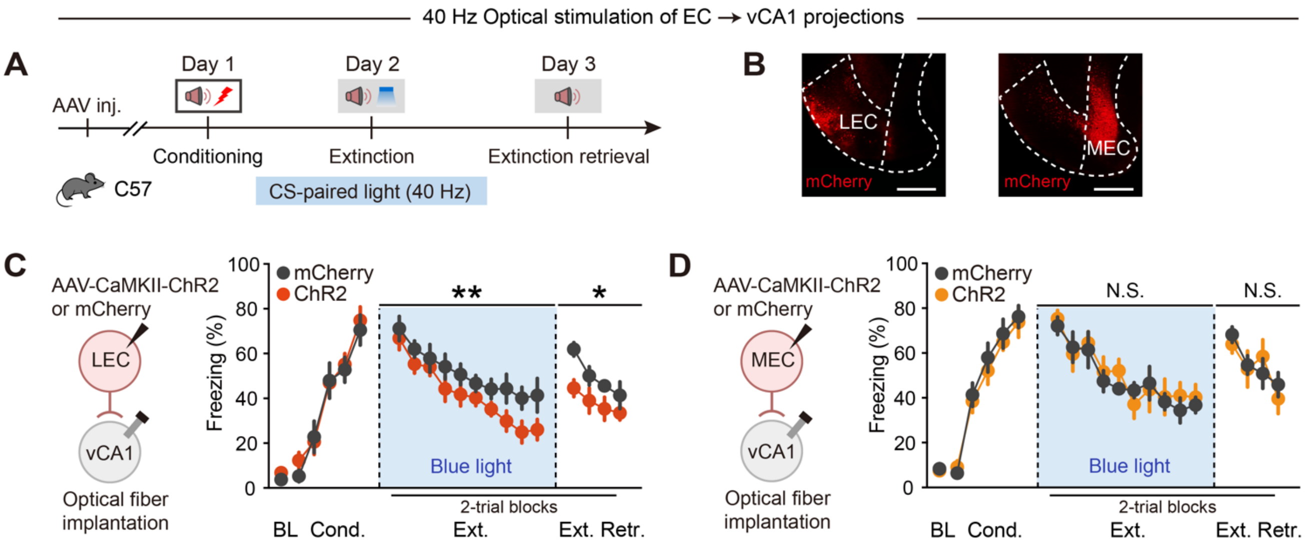

Supplemental Figure 27. Effects of 40 Hz optical stimulation of LEC-vCA1 or MEC-vCA1 on extinction training and retrieval. (**A**) Experimental design of 40 Hz optical stimulation of EC→vCA1 projections. CS is paired with 40 Hz blue light during extinction training. (**B**) Representative images of virus expression. Scale bar, 500 µm. (**C** and **D**) Effects on optical activating LEC→vCA1 projection (**C**) and MEC→vCA1 projection (**D**) on extinction training and extinction retrieval. Schematics of AAV injections (left). Time courses of freezing responses to the CS during fear conditioning, extinction training and extinction retrieval sessions (right). Statistics are as follows: main effect of AAV, (**C**) conditioning, $F_{1,13} = 0.1852$, $P = 0.6740$; extinction training, $F_{1,13} = 14.39$, $P = 0.0022$; extinction retrieval, $F_{1,13} = 7.201$, $P = 0.0188$. mCherry group, $n = 7$ mice, ChR2 group, $n = 8$ mice. (**D**) conditioning, $F_{1,12} = 0.2787$, $P = 0.6072$; extinction training, $F_{1,12} = 0.3980$, $P = 0.5399$; extinction retrieval, $F_{1,12} = 0.03806$, $P = 0.8486$. mCherry group, $n = 7$ mice, ChR2 group, $n = 7$ mice. Data are mean ± SEM. N.S., no significant difference, *$P < 0.05$, **$P < 0.01$. Repeated measures two-way ANOVA in (**C** and **D**).

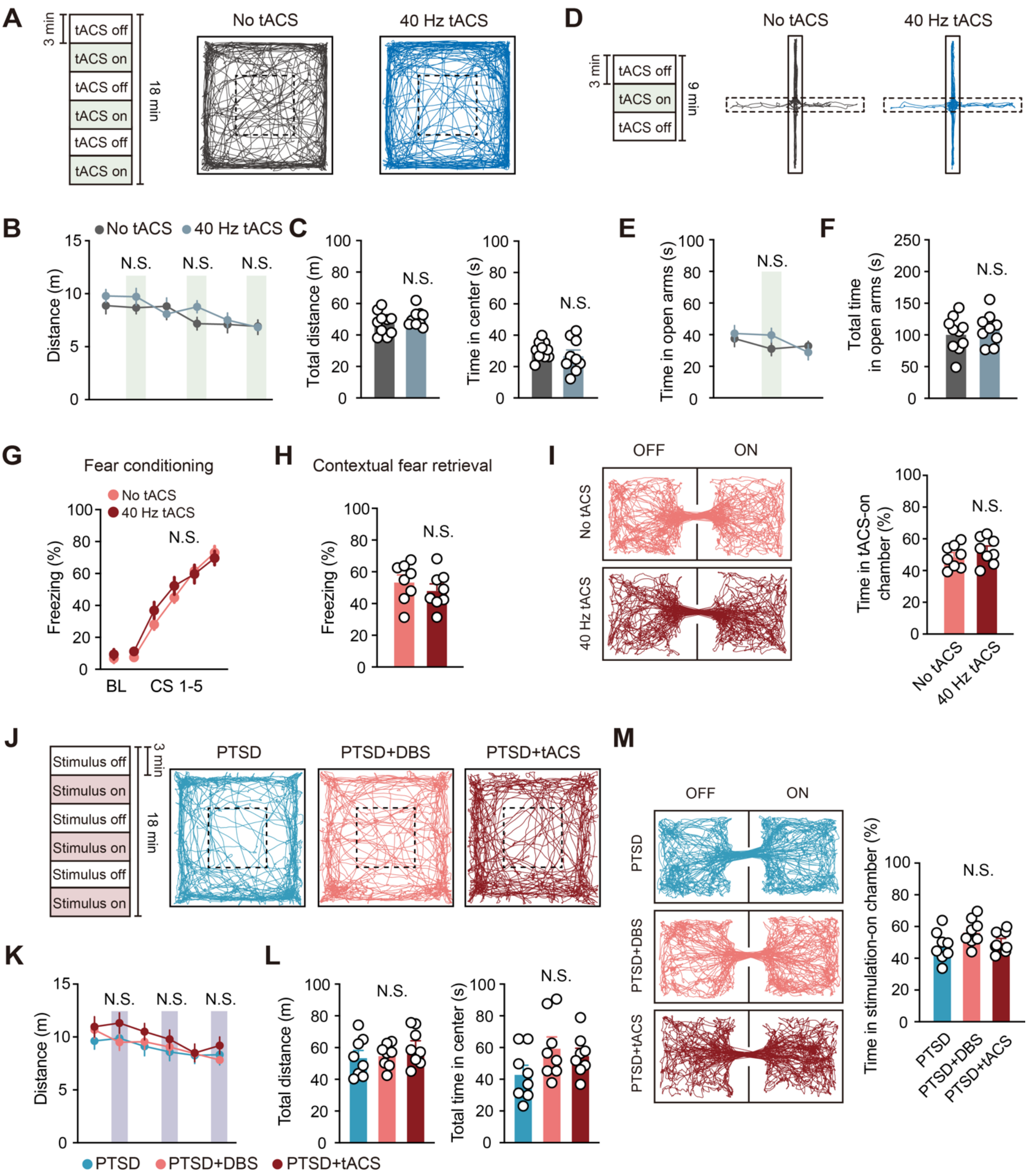

Supplemental Figure 28. Effects of low-gamma stimulation of the LEC→vCA1 circuit on fear conditioning, contextual fear retrieval, and non-task related place preference assays. (**A** and **D**) Schematics of experimental design of the locomotor and anxiety-related activity for low-gamma LEC tACS. (**B**) Effect of LEC tACS on locomotor activity in OFT. The distance traveled in OFT in a 3-min tACS-on (purple box) and tACS-off period. (**C**) Total distances (left) and total time in center (right) in OFT. No tACS group, n = 10 mice; tACS group, n = 9 mice. (**E**) Effect of LEC tACS on anxiety-related activity in EPM. The time spent exploring open arms in a 3-min tACS-on (purple box) and tACS-off period. (**F**) Total time spent exploring open arms. No tACS group, n = 10 mice; tACS group,

n = 9 mice. (**G**) Time courses of freezing responses. Statistics are as follows: main effect of AAV, conditioning, $F_{1,14} = 0.6735$, $P = 0.4256$. (**H**) Freezing responses during contextual fear retrieval. (**I**) Effect of LEC tACS on RTPA or RTPP. Representative traces (left) and the durations of time spent in the tACS-on chamber (right). *n* = 8 mice per group. (**J–L**) The same as (**A–C**) for PTSD mice, PTSD+DBS (vCA1, 40 Hz) mice and PTSD+tACS (LEC, 40 Hz) mice. *n* = 8 mice per group. (**M**) Effect of vCA1 DBS or LEC tACS on RTPA or RTPP in PTSD mice. Representative traces (left) and the durations of time spent in the stimulation-on chamber (right). *n* = 8 mice per group. Repeated measures two-way ANOVA with Sidak's multiple comparisons test in (**B**, **E**, **G**, and **K**), unpaired Student's *t* test in (**C**, **F**, **H**, and **I**) and one-way ANOVA with Tukey's multiple comparisons test in (**L** and **M**). Data are mean ± SEM. N.S., no significant difference.

Supplemental References

1. Stamatakis AM, and Stuber GD. Activation of lateral habenula inputs to the ventral midbrain promotes behavioral avoidance. *Nat Neurosci.* 2012;15(8):1105-7.
2. Zhu Y, Wienecke CF, Nachtrab G, and Chen X. A thalamic input to the nucleus accumbens mediates opiate dependence. *Nature.* 2016;530(7589):219-22.
3. Xiao C, Wei J, Zhang GW, Tao C, Huang JJ, Shen L, et al. Glutamatergic and GABAergic neurons in pontine central gray mediate opposing valence-specific behaviors through a global network. *Neuron.* 2023;111(9):1486-503 e7.
4. Lee B, Pothula S, Wu M, Kang H, Girgenti MJ, Picciotto MR, et al. Positive modulation of N-methyl-D-aspartate receptors in the mPFC reduces the spontaneous recovery of fear. *Mol Psychiatry.* 2022;27(5):2580-9.
5. Vinck M, Oostenveld R, van Wingerden M, Battaglia F, and Pennartz CM. An improved index of phase-synchronization for electrophysiological data in the presence of volume-conduction, noise and sample-size bias. *Neuroimage.* 2011;55(4):1548-65.
6. Lowet E, Kondabolu K, Zhou S, Mount RA, Wang Y, Ravasio CR, et al. Deep brain stimulation creates informational lesion through membrane depolarization in mouse hippocampus. *Nat Commun.* 2022;13(1):7709.
7. Dogdas B, Stout D, Chatziioannou AF, and Leahy RM. Digimouse: a 3D whole body mouse atlas from CT and cryosection data. *Phys Med Biol.* 2007;52(3):577-87.
8. Alekseichuk I, Mantell K, Shirinpour S, and Opitz A. Comparative modeling of transcranial magnetic and electric stimulation in mouse, monkey, and human. *Neuroimage.* 2019;194:136-48.
9. Yushkevich PA, Yang G, and Gerig G. ITK-SNAP: An interactive tool for semi-automatic

segmentation of multi-modality biomedical images. *Annu Int Conf IEEE Eng Med Biol Soc.* 2016;2016:3342-5.

10. Tran AP, Yan S, and Fang Q. Improving model-based functional near-infrared spectroscopy analysis using mesh-based anatomical and light-transport models. *Neurophotonics.* 2020;7(1):015008.

11. Geuzaine C, and Remacle J-F. Gmsh: A 3-D finite element mesh generator with built-in pre- and post-processing facilities. *Int J Numer Methods Eng.* 2009;79(11):1309-31.

12. Tao C, Zhang GW, Huang JJ, Li Z, Tao HW, and Zhang LI. The medial preoptic area mediates depressive-like behaviors induced by ovarian hormone withdrawal through distinct GABAergic projections. *Nat Neurosci.* 2023;26(9):1529-40.
